# Supplementary material for: Identification of Genomic Signatures for Colorectal Cancer Survival Using Exploratory Data Mining
Source: Int J Mol Sci. 2024 Mar 12;25(6):3220. doi: 10.3390/ijms25063220 (PMC10970183; doi:10.3390/ijms25063220)
Supplement: Supplementary file 1 [file ijms-25-03220-s001.zip › Supplementary_MM_revised_changes-accepted.pdf]

Supplementary Material

Identification of genomic signatures for colorectal cancer survival using an exploratory data mining.

Hummel et al.

CONTENTS:

- **Supplementary Materials and Methods:** Detailed Material and Methods.
- **Supplementary Table S1:** F1CDx marker panel.
- **Supplementary Table S2:** Patient Characteristics.
- **Supplementary Table S3:** Top Mutation Genomic Signatures collected from CRC Population Subgroups.
- **Supplementary Table S4:** Marker lists for each genomic signature.
- **Supplementary Table S5:** Genomic Signatures from CRC Subgroups for Survival Analysis.
- **Supplementary Table S6.** Hazard Ratios from Survival Analysis.
- **Supplementary Table S7:** Mutation frequency in studied samples.
- **Supplementary Table S8.** Feature descriptions for variables used in exploratory analysis.

DETAILED MATERIALS AND METHODS

Patient population and data set. CRC patient data for the training cohort was accessed using the cBioPortal for Cancer Genomics (Colorectal Adenocarcinoma (TCGA, Firehose Legacy), https://www.cbioportal.org/study/summary?id=coadread_tcga [29,30]) to collect the appropriate molecular and clinical attributes for our study based upon data generated by the TCGA Research Network under Broad Institute GDAC TCGA Analysis Pipeline License (https://gdc.cancer.gov/)[31]. Clinical attributes used to generate our collection of observed population subgroups is included in Supplementary Table S6. The molecular profiles of 321/324 of the F1CDx target genes (Supplementary Table S1) were extracted as copy-number alterations (CNA) represented as putative copy-number calls for 378 cases using GISTIC 2.0 (Genomic Identification of Significant Targets in Cancer, version 2.0.22)[28] available from URL: (https://doi.org/10.1186/gb-2011-12-4-r41). The analysis of our CRC cohort was performed using the clinical aspects detailed in Supplementary Table S6 combined with these discrete data types, whereby: (a.) -2 = homozygous deletion; (b.) -1 = hemizygous deletion; (c.) 0 = normal/neutral; (d.) 1 = gain; and (e.) 2 = high level amplification. The rationale behind this selection is predicted on the ability of these data to reveal the regions of a patient's genome amplified, or deleted, in a significant manner. The nature of these data type aligns with both our experimental objectives as well as XAI model implementation. Supplementary Table S2 describes the patient baseline characteristics of the cohort organized by CRC Stage 2. The AJCC 7th edition [2,3] along with recommended option within Clinical Practice Guidelines in Oncology NCCN guidelines for Stage 2 and/or Stage 3 Colorectal Cancer [4,5] are both observed in both collection and interpretation in the context of investigating recurrence for each of the three CRC groups. The mean disease-free survival time in months for CRC Stage 2 was determined to be ~29.10 months, and ~23.56 months for CRC Stage 3. The F1CDx assay (Foundation Medicine, Roche, Cambridge, MA) utilizes Comprehensive Genomic Profiling (CGP) as its method of testing tumors, using Next-Generation Sequencing (NGS) technologies to detect four main classes of alterations implicated in cancer growth: (a.) base substitutions; (b.) insertions and deletions; (c.) copy number alterations (CNAs); and (c.) rearrangements or fusions. F1CDx is a single tissue-based test that analyzes 324 genes in DNA of solid tumors (Supplementary Table S1). Its operational components are predicated on diagnostic indications such as microsatellite instability (MSI), tumor-mutational burden (TMB), FDA-approved targeted therapies, and genomic signatures to inform patient benefit from multiple targeted therapies, for various cancers. Implementing our stratification model with this companion diagnostic as our genomic framework aligns with the rationale of our aims as well as the experimental design. The strategy of formulating a recurrence probability inference model using data types that mirror those collected from FFPE tissue samples of patients is logical and novel.

Data Preprocessing and Implementation. Data preprocessing was carried out using the R-package *tidyverse* (version 1.3.2)[32]. First, we collected the genes consistent with the F1CDx companion diagnostic to create the genomic template. Second, we combined this genomic template with clinical variables according to patient ID. Third, we categorized variables that are continuous, (e.g., disease-free survival times), or discrete, (e.g., race) to reduce their cardinality. Cardinality refers to the increased complexity that occurs in the number of possible patterns between variables based on their levels, or values. Constraining variable levels (i.e., commonly referred to as 'One-Hot Encoding') was applied for the proper function of our artificial intelligence algorithm (XAI). Imputation of missing values was carried out using the R-package *mice*, (version 3.15.0)[33]. Variables with missing values exceeding 40% were not included in this study. Top-ranking subgroup results were identified via a unique provision collected upon implementation of our algorithm called the 'J-index'[18]. The J-Index, or J-value, enabled ranking the subgroups by scoring the most relevant pattern indication(s) defining them, as a numerical value. Identifying a subgroup requires population variables, (clinical variables) and designated measurement variables (genomic variables) to produce patterns, (i.e., genomic signature mutations)[18].

Furthermore, the J-Index reflected the strength of the contrast patterns of the measurement variables in relation to the population variables used to define, or identify each subgroup, as mathematical representation. Patients absent from a particular subgroup were classified as a separate cohort, (i.e., 'outside the group'), for purposes of analysis. Upon completion of data preprocessing, multiple hypothesis testing was conducted with the Benjamini-Hochberg procedure using a false discovery rate (Q $\leq$ 10%)[2,4,20,21,34]. The genomic signature(s) for each subgroup population were identified by matching the patterns of the observed result to the specific clinical variables and their values. This resulted in a list of distinct gene mutations for each of the two classes in the population subgroup, respective to the clinical variables that define it. Each of the two genomic signatures were sorted by ascending adjusted p-value to determine the order of collecting the identified mutations among the patient groups. This was carried out using the R-package *sqldf* (version 0.4.11)[35] to accurately identify the presence or absence of a given genomic signature.

Disease-Free Survival Analysis. Kaplan-Meier survival analysis was performed using the corresponding feature vectors for both clinical indications and identified genomic signatures from the top subgroup collections to assess "disease-free survival" (DFS), or commonly referred to as recurrence, using the R-packages *survival* (version 3.5.0)[34,36], *survminer* (version 0.4.9)[37], and *ggsurvfit* (version 0.2.1)[38]. This approach allows one to quantitate the effectiveness for each prospective clinical phenotype against their respective genomic signatures. Survival probabilities were estimated using DFS as the event measured and a p-value $\leq$ 0.05 was considered significant.

ROC-AUC Analytical Validation. The performance of the identified genomic signatures was evaluated using the Receiving-Operating Characteristic (ROC) analysis implemented using R-package *ROCit*, (version 2.1.1)[39] in the R environment (version 4.2.2). Empirical, binormal, and non-parametric test functions were calculated for every ROC analysis performed to determine which genomic signature(s) best discriminates recurrence. We reported AUCs for the three ROC functions to mitigate bias during their comparison [39,40]. The probability of inferring a likelihood of recurrence was assessed among patients given the top performing genomic signatures individually (Figure 2), the combinations of genomic signatures, in conjunction with the clinical indications of consensus molecular subtypes (Figure 4), and validation dataset AACR GENIE BPC CRC 2.0-PUBLIC (http://cbioportal.org/genie/)[22] using the identified 32-gene marker panel (Figure 5).

Marker panel summarization and validation in an independent cohort. The gene composition of the three top performing genomic signatures in CRC Stage 2 subjects (i.e., MG02PS03, MG02PS04 and MG02PS05) was compared by UpSet plots using the R-package *VennDetail* (version 1.14.0)[41]. A marker panel consisting of 32-genes (Supplementary Table S5) was selected and further validated in an independent dataset (AACR GENIE BPC CRC 2.0-PUBLIC, http://cbioportal.org/genie/)[41]. ROC-AUC analysis for the validation of the 32-gene genomic signature was conducted as detailed above.

Enrichment Analysis. R-package *randomForestSRC* (version 3.2.0)[42–44] was used to assess competing risks and variable importance in the downstream analysis of validation dataset (AACR GENIE BPC CRC 2.0-PUBLIC, http://cbioportal.org/genie/)[22]. R-package *randomForestSRC* was selected for its disease-free survival forest algorithm (ntree = 2500) to evaluate these 32 prognostic genes and evaluate the contributions, or importance, of each of the variables towards these recurrence predictions, thereby acting to better assist us in our biological interpretation of these events [22,40,42–44].

SUPPLEMENTARY TABLES

Table S1. F1CDx marker panel.

Genes	Name	Description
TNFRSF14	TNF receptor superfamily member 14	This gene encodes a member of the TNF (tumor necrosis factor) receptor superfamily. The encoded protein functions in signal transduction pathways that activate inflammatory and inhibitory T-cell immune response. It binds herpes simplex virus (HSV) viral envelope glycoprotein D (gD), mediating its entry into cells. Alternative splicing results in multiple transcript variants. [provided by RefSeq, Jul 2014]
ERRFI1	ERBB receptor feedback inhibitor 1	ERRFI1 is a cytoplasmic protein whose expression is upregulated with cell growth (Wick et al., 1995 [PubMed 7641805]). It shares significant homology with the protein product of rat gene-33, which is induced during cell stress and mediates cell signaling (Makkinje et al., 2000 [PubMed 10749885]; Fiorentino et al., 2000 [PubMed 11003669]).[supplied by OMIM, Mar 2008]
MTOR	mechanistic target of rapamycin kinase	The protein encoded by this gene belongs to a family of phosphatidylinositol kinase-related kinases. These kinases mediate cellular responses to stresses such as DNA damage and nutrient deprivation. This kinase is a component of two distinct complexes, mTORC1, which controls protein synthesis, cell growth and proliferation, and mTORC2, which is a regulator of the actin cytoskeleton, and promotes cell survival and cell cycle progression. This protein acts as the target for the cell-cycle arrest and immunosuppressive effects of the FKBP12-rapamycin complex. Inhibitors of mTOR are used in organ transplants as immunosuppressants and are being evaluated for their therapeutic potential in SARS-CoV-2 infections. Mutations in this gene are associated with Smith-Kingsmore syndrome and somatic focal cortical dysplasia type II. The ANGPTL7 gene is in an intron of this gene. [provided by RefSeq, Aug 2020]
SPEN	spen family transcriptional repressor	This gene encodes a hormone inducible transcriptional repressor. Repression of transcription by this gene product can occur through interactions with other repressors, by the recruitment of proteins involved in histone deacetylation, or through sequestration of transcriptional activators. The product of this gene contains a carboxy-terminal domain that permits binding to other corepressor proteins. This domain also permits interaction with members of the NuRD complex, a nucleosome remodeling protein complex that contains deacetylase activity. In addition, this repressor contains several RNA recognition motifs that confer binding to a steroid receptor RNA coactivator; this binding can modulate the activity of both liganded and nonligand steroid receptors. [provided by RefSeq, Jul 2008]
SDHB	succinate dehydrogenase complex iron sulfur subunit B	Complex II of the respiratory chain, which is specifically involved in the oxidation of succinate, carries electrons from FADH to CoQ. The complex is composed of four nuclear-encoded subunits and is localized in the mitochondrial inner membrane. The iron-sulfur subunit is highly conserved and contains three cysteine-rich clusters which may comprise the iron-sulfur centers of the enzyme. Sporadic and familial mutations in this gene result in paragangliomas and pheochromocytoma and support a link between mitochondrial dysfunction and tumorigenesis. [provided by RefSeq, Jul 2008]
ID3	inhibitor of DNA binding 3, HLH protein	The protein encoded by this gene is a helix-loop-helix (HLH) protein that can form heterodimers with other HLH proteins. However, the encoded protein lacks a basic DNA-binding domain and therefore inhibits the DNA binding of any HLH protein with which it interacts. [provided by RefSeq, Aug 2011]
ARID1A	AT-rich interaction domain 1A	This gene encodes a member of the SWI/SNF family, whose members have helicase and ATPase activities and are thought to regulate transcription of certain genes by altering the chromatin structure around those genes. The encoded protein is part of the large ATP-dependent chromatin remodeling complex SNF/SWI, which is required for transcriptional activation of genes normally repressed by chromatin. It possesses at least two conserved domains that could be important for its function. First, it has a DNA-binding domain that can specifically bind an AT-rich DNA sequence known to be recognized by a SNF/SWI complex at the beta-globin locus. Second, the C-terminus of the protein can stimulate glucocorticoid receptor-dependent transcriptional activation. It is thought that the protein encoded by this gene confers specificity to the SNF/SWI complex and may recruit the complex to its targets through either protein-DNA or protein-protein interactions. Two transcript variants encoding different isoforms have been found for this gene. [provided by RefSeq, Jul 2008]
HDAC1	histone deacetylase 1	Histone acetylation and deacetylation, catalyzed by multisubunit complexes, play a key role in the regulation of eukaryotic gene expression. The protein encoded by this gene belongs to the histone deacetylase/acuc/apha family and is a component of the histone deacetylase complex. It also interacts with retinoblastoma tumor-suppressor protein and this complex is a key element in the control of cell proliferation and differentiation. Together with metastasis-associated protein-2, it deacetylates p53 and modulates its effect on cell growth and apoptosis. [provided by RefSeq, Jul 2008]
CSF3R	colony stimulating factor 3 receptor	The protein encoded by this gene is the receptor for colony stimulating factor 3, a cytokine that controls the production, differentiation, and function of granulocytes. The encoded protein, which is a member of the family of cytokine receptors, may also function in some cell surface adhesion or recognition processes. Alternatively, spliced transcript variants have been described. Mutations in this gene are a cause of Kostmann syndrome, also known as severe congenital neutropenia. [provided by RefSeq, Aug 2010]
MPL	MPL proto-oncogene, thrombopoietin receptor	In 1990 an oncogene, v-mpl, was identified from the murine myeloproliferative leukemia virus that was capable of immortalizing bone marrow hematopoietic cells from different lineages. In 1992 the human homologue, named, c-mpl, was cloned. Sequence data revealed that c-mpl encoded a protein that was homologous with members of the hematopoietic receptor superfamily. Presence of anti-sense oligodeoxynucleotides of c-mpl inhibited megakaryocyte colony formation. The ligand for c-mpl, thrombopoietin, was cloned in 1994. Thrombopoietin was shown to be the major regulator of megakaryocytopoiesis and platelet formation. The protein encoded by the c-mpl gene, CD110, is a 635 amino acid transmembrane domain, with two extracellular cytokine receptor domains and two intracellular cytokine receptor box motifs. TPO-R deficient mice were severely thrombocytopenic, emphasizing the important role of CD110 and thrombopoietin in megakaryocyte and platelet formation. Upon binding of thrombopoietin CD110 is dimerized and the JAK family of non-receptor tyrosine kinases, as well as the STAT family, the MAPK

		family, the adaptor protein Shc and the receptors themselves become tyrosine phosphorylated. [provided by RefSeq, Jul 2008]
MUTYH	mutY DNA glycosylase	This gene encodes a DNA glycosylase involved in oxidative DNA damage repair. The enzyme excises adenine bases from the DNA backbone at sites where adenine is inappropriately paired with guanine, cytosine, or 8-oxo-7,8-dihydroguanine, a major oxidatively damaged DNA lesion. The protein is localized to the nucleus and mitochondria. This gene product is thought to play a role in signaling apoptosis by the introduction of single-strand breaks following oxidative damage. Mutations in this gene result in heritable predisposition to colorectal cancer, termed MUTYH-associated polyposis (MAP). Multiple transcript variants encoding different isoforms have been found for this gene. [provided by RefSeq, Apr 2017]
RAD54L	RAD54 like	The protein encoded by this gene belongs to the DEAD-like helicase superfamily, and shares similarity with Saccharomyces cerevisiae Rad54, a protein known to be involved in the homologous recombination and repair of DNA. This protein has been shown to play a role in homologous recombination related repair of DNA double-strand breaks. The binding of this protein to double-strand DNA induces a DNA topological change, which is thought to facilitate homologous DNA paring, and stimulate DNA recombination. Alternative splicing results in multiple transcript variants encoding the same protein. [provided by RefSeq, Dec 2008]
MKNK1	MAPK interacting serine/threonine kinase 1	This gene encodes a Ser/Thr protein kinase that interacts with and is activated by ERK1 and p38 mitogen-activated protein kinases, and thus may play a role in the response to environmental stress and cytokines. This kinase may also regulate transcription by phosphorylating eIF4E via interaction with the C-terminal region of eIF4G. Alternatively spliced transcript variants have been noted for this gene. [provided by RefSeq, Jan 2012]
CDKN2C	cyclin dependent kinase inhibitor 2C	The protein encoded by this gene is a member of the INK4 family of cyclin-dependent kinase inhibitors. This protein has been shown to interact with CDK4 or CDK6, and prevent the activation of the CDK kinases, thus function as a cell growth regulator that controls cell cycle G1 progression. Ectopic expression of this gene was shown to suppress the growth of human cells in a manner that appears to correlate with the presence of a wild-type RB1 function. Studies in the knockout mice suggested the roles of this gene in regulating spermatogenesis, as well as in suppressing tumorigenesis. Two alternatively spliced transcript variants of this gene, which encode an identical protein, have been reported. [provided by RefSeq, Jul 2008]
JUN	Jun proto-oncogene, AP-1 transcription factor subunit	This gene is the putative transforming gene of avian sarcoma virus 17. It encodes a protein which is highly similar to the viral protein, and which interacts directly with specific target DNA sequences to regulate gene expression. This gene is intron less and is mapped to 1p32-p31, a chromosomal region involved in both translocations and deletions in human malignancies. [provided by RefSeq, Jul 2008]
JAK1	Janus kinase 1	This gene encodes a membrane protein that is a member of a class of protein-tyrosine kinases (PTK) characterized by the presence of a second phosphotransferase-related domain immediately N-terminal to the PTK domain. The encoded kinase phosphorylates STAT proteins (signal transducers and activators of transcription) and plays a key role in interferon-alpha/beta, interferon-gamma, and cytokine signal transduction. This gene plays a crucial role in effecting the expression of genes that mediate inflammation, epithelial remodeling, and metastatic cancer progression. This gene is a key component of the interleukin-6 (IL-6)/JAK1/STAT3 immune and inflammation response and is a therapeutic target for alleviating cytokine storms. The kinase activity of this gene is directly inhibited by the suppressor of cytokine signaling 1 (SOCS1) protein. Alternative splicing results in multiple transcript variants. [provided by RefSeq, Jul 2020]
FUBP1	far upstream element binding protein 1	The protein encoded by this gene is a single stranded DNA-binding protein that binds to multiple DNA elements, including the far upstream element (FUSE) located upstream of c-myc. Binding to FUSE occurs on the non-coding strand, and is important to the regulation of c-myc in undifferentiated cells. This protein contains three domains, an amphipathic helix N-terminal domain, a DNA-binding central domain, and a C-terminal transactivation domain that contains three tyrosine-rich motifs. The N-terminal domain is thought to repress the activity of the C-terminal domain. This protein is also thought to bind RNA and contains 3'-5' helicase activity with in vitro activity on both DNA-DNA and RNA-RNA duplexes. Aberrant expression of this gene has been found in malignant tissues, and this gene is important to neural system and lung development. Binding of this protein to viral RNA is thought to play a role in several viral diseases, including hepatitis C and hand, foot and mouth disease. Alternative splicing results in multiple transcript variants. [provided by RefSeq, Dec 2014]
NRAS	NRAS proto-oncogene, GTPase	This is an N-ras oncogene encoding a membrane protein that shuttles between the Golgi apparatus and the plasma membrane. This shuttling is regulated through palmitoylation and depalmitoylation by the ZDHHC9-GOLGA7 complex. The encoded protein, which has intrinsic GTPase activity, is activated by a guanine nucleotide-exchange factor and inactivated by a GTPase activating protein. Mutations in this gene have been associated with somatic rectal cancer, follicular thyroid cancer, autoimmune lymphoproliferative syndrome, Noonan syndrome, and juvenile myelomonocytic leukemia. [provided by RefSeq, Jun 2011]
FAM46C	TENT5C terminal nucleotidyltransferase 5C	Nucleotidyltransferase that act as a non-canonical poly(A) RNA polymerase which enhances mRNA stability and gene expression. Mainly targets mRNAs encoding endoplasmic reticulum-targeted protein and may be involved in induction of cell death.
HSD3B1	hydroxy-delta-5-steroid dehydrogenase, 3 beta- and steroid delta-isomerase 1	The protein encoded by this gene is an enzyme that catalyzes the oxidative conversion of delta-5-3-beta-hydroxysteroid precursors into delta-4-ketosteroids, which leads to the production of all classes of steroid hormones. The encoded protein also catalyzes the interconversion of 3-beta-hydroxy- and 3-keto-5-alpha-androstane steroids. [provided by RefSeq, Jun 2016]
NOTCH2	notch receptor 2	This gene encodes a member of the Notch family. Members of this Type 1 transmembrane protein family share structural characteristics including an extracellular domain consisting of multiple epidermal growth factor-like (EGF) repeats, and an intracellular domain consisting of multiple, different domain types. Notch family members play a role in a variety of developmental processes by controlling cell fate decisions. The Notch signaling network is an evolutionarily conserved intercellular signaling pathway which regulates interactions between physically adjacent cells. In Drosophila, notch interaction with its cell-bound ligands (delta, serrate) establishes an intercellular signaling pathway that plays a key role in development. Homologues of the notch-ligands have also been identified in human, but precise interactions between these ligands and the human notch homologues remain to be determined. This protein is cleaved in the trans-Golgi network, and presented on the cell surface as a heterodimer. This protein functions as a receptor for membrane bound ligands, and may play a

		role in vascular, renal and hepatic development. Two transcript variants encoding different isoforms have been found for this gene. [provided by RefSeq, Jan 2011]
MCL1	MCL1 apoptosis regulator, BCL2 family member	This gene encodes an anti-apoptotic protein, which is a member of the Bcl-2 family. Alternative splicing results in multiple transcript variants. The longest gene product (isoform 1) enhances cell survival by inhibiting apoptosis while the alternatively spliced shorter gene products (isoform 2 and isoform 3) promote apoptosis and are death-inducing. [provided by RefSeq, Oct 2010]
NTRK1	neurotrophic receptor tyrosine kinase 1	This gene encodes a member of the neurotrophic tyrosine kinase receptor (NTKR) family. This kinase is a membrane-bound receptor that, upon neurotrophin binding, phosphorylates itself and members of the MAPK pathway. The presence of this kinase leads to cell differentiation and may play a role in specifying sensory neuron subtypes. Mutations in this gene have been associated with congenital insensitivity to pain, anhidrosis, self-mutilating behavior, cognitive disability and cancer. Alternate transcriptional splice variants of this gene have been found, but only three have been characterized to date. [provided by RefSeq, Jul 2008]
SDHC	succinate dehydrogenase complex subunit C	This gene encodes one of four nuclear-encoded subunits that comprise succinate dehydrogenase, also known as mitochondrial complex II, a key enzyme complex of the tricarboxylic acid cycle and aerobic respiratory chains of mitochondria. The encoded protein is one of two integral membrane proteins that anchor other subunits of the complex, which form the catalytic core, to the inner mitochondrial membrane. There are several related pseudogenes for this gene on different chromosomes. Mutations in this gene have been associated with paragangliomas. Alternatively, spliced transcript variants have been described. [provided by RefSeq, May 2013]
DDR2	discoidin domain receptor tyrosine kinase 2	This gene encodes a member of the discoidin domain receptor subclass of the receptor tyrosine kinase (RTKs) protein family. RTKs play a key role in the communication of cells with their microenvironment. The encoded protein is a collagen-induced receptor that activates signal transduction pathways involved in cell adhesion, proliferation, and extracellular matrix remodeling. This protein is expressed in numerous cell types and may alos be involved in wound repair and regulate tumor growth and invasiveness. Mutations in this gene are the cause of short limb-hand type spondylometaepiphyseal dysplasia. [provided by RefSeq, Aug 2017]
CDC73	cell division cycle 73	This gene encodes a tumor suppressor that is involved in transcriptional and post-transcriptional control pathways. The protein is a component of the the PAF protein complex, which associates with the RNA polymerase II subunit POLR2A and with a histone methyltransferase complex. This protein appears to facilitate the association of 3' mRNA processing factors with actively transcribed chromatin. Mutations in this gene have been linked to hyperparathyroidism-jaw tumor syndrome, familial isolated hyperparathyroidism, and parathyroid carcinoma. [provided by RefSeq, Jul 2009]
BTG2	BTG anti-proliferation factor 2	The protein encoded by this gene is a member of the BTG/Tob family. This family has structurally related proteins that appear to have antiproliferative properties. This encoded protein is involved in the regulation of the G1/S transition of the cell cycle. [provided by RefSeq, Jul 2008]
PIK3C2B	phosphatidylinositol-4-phosphate 3-kinase catalytic subunit type 2 beta	The protein encoded by this gene belongs to the phosphoinositide 3-kinase (PI3K) family. PI3-kinases play roles in signaling pathways involved in cell proliferation, oncogenic transformation, cell survival, cell migration, and intracellular protein trafficking. This protein contains a lipid kinase catalytic domain as well as a C-terminal C2 domain, a characteristic of class II PI3-kinases. C2 domains act as calcium-dependent phospholipid binding motifs that mediate translocation of proteins to membranes and may also mediate protein-protein interactions. The PI3-kinase activity of this protein is sensitive to low nanomolar levels of the inhibitor wortmannin. The C2 domain of this protein was shown to bind phospholipids but not Ca2+, which suggests that this enzyme may function in a calcium-independent manner. [provided by RefSeq, Jul 2008]
MDM4	MDM4 regulator of p53	This gene encodes a nuclear protein that contains a p53 binding domain at the N-terminus and a RING finger domain at the C-terminus and shows structural similarity to p53-binding protein MDM2. Both proteins bind the p53 tumor suppressor protein and inhibit its activity and have been shown to be overexpressed in a variety of human cancers. However, unlike MDM2 which degrades p53, this protein inhibits p53 by binding its transcriptional activation domain. This protein also interacts with MDM2 protein via the RING finger domain and inhibits the latter's degradation. So this protein can reverse MDM2-targeted degradation of p53, while maintaining suppression of p53 transactivation and apoptotic functions. Alternatively, spliced transcript variants encoding different isoforms have been noted for this gene. [provided by RefSeq, Feb 2011]
IKBKE	inhibitor of nuclear factor kappa B kinase subunit epsilon	IKBKE is a noncanonical I-kappa-B (see MIM 164008) kinase (IKK) that is essential for regulating antiviral signaling pathways. IKBKE has also been identified as a breast cancer (MIM 114480) oncogene and is amplified and overexpressed in over 30% of breast carcinomas and breast cancer cell lines (Hutti et al., 2009 [PubMed 19481526]).[supplied by OMIM, Oct 2009]
H3F3A	H3.3 histone A	Histones are basic nuclear proteins that are responsible for the nucleosome structure of the chromosomal fiber in eukaryotes. Two molecules of each of the four core histones (H2A, H2B, H3, and H4) form an octamer, around which approximately 146 bp of DNA is wrapped in repeating units, called nucleosomes. The linker histone, H1, interacts with linker DNA between nucleosomes and functions in the compaction of chromatin into higher order structures. This gene contains introns, and its mRNA is polyadenylated, unlike most histone genes. The protein encoded is a replication-independent member of the histone H3 family. [provided by RefSeq, Jul 2008]
PARP1	poly(ADP-ribose) polymerase 1	This gene encodes a chromatin-associated enzyme, poly (ADP-ribosyl) transferase, which modifies various nuclear proteins by poly(ADP-ribosyl)ation. The modification is dependent on DNA and is involved in the regulation of various important cellular processes such as differentiation, proliferation, and tumor transformation and also in the regulation of the molecular events involved in the recovery of cell from DNA damage. In addition, this enzyme may be the site of mutation in Fanconi anemia and may participate in the pathophysiology of type I diabetes. [provided by RefSeq, Jul 2008]
FH	fumarate hydratase	The protein encoded by this gene is an enzymatic component of the tricarboxylic acid (TCA) cycle, or Krebs cycle, and catalyzes the formation of L-malate from fumarate. It exists in both a cytosolic form and an N-terminal extended form, differing only in the translation start site used. The N-terminal extended form is targeted to the mitochondrion, where the removal of the extension generates the same form as in the cytoplasm. It is similar to some thermostable class II fumarases and functions as a homotetramer. Mutations in this gene can cause fumarase deficiency and lead to progressive encephalopathy. [provided by RefSeq, Jul 2008]

AKT3	AKT serine/threonine kinase 3	The protein encoded by this gene is a member of the AKT, also called PKB, serine/threonine protein kinase family. AKT kinases are known to be regulators of cell signaling in response to insulin and growth factors. They are involved in a wide variety of biological processes including cell proliferation, differentiation, apoptosis, tumorigenesis, as well as glycogen synthesis and glucose uptake. This kinase has been shown to be stimulated by platelet-derived growth factor (PDGF), insulin, and insulin-like growth factor 1 (IGF1). Alternatively splice transcript variants encoding distinct isoforms have been described. [provided by RefSeq, Jul 2008]
MYCN	MYCN proto-oncogene, bHLH transcription factor	This gene is a member of the MYC family and encodes a protein with a basic helix-loop-helix (bHLH) domain. This protein is located in the nucleus and must dimerize with another bHLH protein in order to bind DNA. Amplification of this gene is associated with a variety of tumors, most notably neuroblastomas. Multiple alternatively spliced transcript variants encoding different isoforms have been found for this gene. [provided by RefSeq, Jun 2014]
DNMT3A	DNA methyltransferase 3 alpha	CpG methylation is an epigenetic modification that is important for embryonic development, imprinting, and X-chromosome inactivation. Studies in mice have demonstrated that DNA methylation is required for mammalian development. This gene encodes a DNA methyltransferase that is thought to function in de novo methylation, rather than maintenance methylation. The protein localizes to the cytoplasm and nucleus and its expression is developmentally regulated. [provided by RefSeq, Mar 2016]
ALK	ALK receptor tyrosine kinase	This gene encodes a receptor tyrosine kinase, which belongs to the insulin receptor superfamily. This protein comprises an extracellular domain, an hydrophobic stretch corresponding to a single pass transmembrane region, and an intracellular kinase domain. It plays an important role in the development of the brain and exerts its effects on specific neurons in the nervous system. This gene has been found to be rearranged, mutated, or amplified in a series of tumors including anaplastic large cell lymphomas, neuroblastoma, and non-small cell lung cancer. The chromosomal rearrangements are the most common genetic alterations in this gene, which result in creation of multiple fusion genes in tumorigenesis, including ALK (chromosome 2)/EML4 (chromosome 2), ALK/RANBP2 (chromosome 2), ALK/ATIC (chromosome 2), ALK/TFG (chromosome 3), ALK/NPM1 (chromosome 5), ALK/SQSTM1 (chromosome 5), ALK/KIF5B (chromosome 10), ALK/CLTC (chromosome 17), ALK/TPM4 (chromosome 19), and ALK/MSN (chromosome X).[provided by RefSeq, Jan 2011]
MSH2	mutS homolog 2	This locus is frequently mutated in hereditary nonpolyposis colon cancer (HNPCC). When cloned, it was discovered to be a human homolog of the E. coli mismatch repair gene mutS, consistent with the characteristic alterations in microsatellite sequences (RER+ phenotype) found in HNPCC. Two transcript variants encoding different isoforms have been found for this gene. [provided by RefSeq, Apr 2012]
MSH6	mutS homolog 6	This gene encodes a member of the DNA mismatch repair MutS family. In E. coli, the MutS protein helps in the recognition of mismatched nucleotides prior to their repair. A highly conserved region of approximately 150 aa, called the Walker-A adenine nucleotide binding motif, exists in MutS homologs. The encoded protein heterodimerizes with MSH2 to form a mismatch recognition complex that functions as a bidirectional molecular switch that exchanges ADP and ATP as DNA mismatches are bound and dissociated. Mutations in this gene may be associated with hereditary nonpolyposis colon cancer, colorectal cancer, and endometrial cancer. Transcripts variants encoding different isoforms have been described. [provided by RefSeq, Jul 2013]
FANCL	FA complementation group L	This gene encodes a ubiquitin ligase that is a member of the Fanconi anemia complementation group (FANC). Members of this group are related by their assembly into a common nuclear protein complex rather than by sequence similarity. This gene encodes the protein for complementation group L that mediates monoubiquitination of FANCD2 as well as FANCI. Fanconi anemia is a genetically heterogeneous recessive disorder characterized by cytogenetic instability, hypersensitivity to DNA crosslinking agents, increased chromosomal breakage, and defective DNA repair. Alternative splicing results in multiple transcript variants. [provided by RefSeq, May 2018]
REL	REL proto-oncogene, NF-kB subunit	This gene encodes a protein that belongs to the Rel homology domain/immunoglobulin-like fold, plexin, transcription factor (RHD/IPT) family. Members of this family regulate genes involved in apoptosis, inflammation, the immune response, and oncogenic processes. This proto-oncogene plays a role in the survival and proliferation of B lymphocytes. Mutation or amplification of this gene is associated with B-cell lymphomas, including Hodgkin's lymphoma. Single nucleotide polymorphisms in this gene are associated with susceptibility to ulcerative colitis and rheumatoid arthritis. Alternative splicing results in multiple transcript variants encoding different isoforms. [provided by RefSeq, Apr 2014]
XPO1	exportin 1	This cell-cycle-regulated gene encodes a protein that mediates leucine-rich nuclear export signal (NES)-dependent protein transport. The protein specifically inhibits the nuclear export of Rev and U snRNAs. It is involved in the control of several cellular processes by controlling the localization of cyclin B, MPAK, and MAPKAP kinase 2. This protein also regulates NFAT and AP-1. [provided by RefSeq, Jan 2015]
MERTK	MER proto-oncogene, tyrosine kinase	This gene is a member of the MER/AXL/TYRO3 receptor kinase family and encodes a transmembrane protein with two fibronectin type-III domains, two Ig-like C2-type (immunoglobulin-like) domains, and one tyrosine kinase domain. Mutations in this gene have been associated with disruption of the retinal pigment epithelium (RPE) phagocytosis pathway and onset of autosomal recessive retinitis pigmentosa (RP). [provided by RefSeq, Jul 2008]
CXCR4	C-X-C motif chemokine receptor 4	This gene encodes a CXC chemokine receptor specific for stromal cell-derived factor-1. The protein has 7 transmembrane regions and is located on the cell surface. It acts with the CD4 protein to support HIV entry into cells and is also highly expressed in breast cancer cells. Mutations in this gene have been associated with WHIM (warts, hypogammaglobulinemia, infections, and myelokathexis) syndrome. Alternate transcriptional splice variants, encoding different isoforms, have been characterized. [provided by RefSeq, Jul 2008]
PDK1	pyruvate dehydrogenase kinase 1	Pyruvate dehydrogenase (PDH) is a mitochondrial multienzyme complex that catalyzes the oxidative decarboxylation of pyruvate and is one of the major enzymes responsible for the regulation of homeostasis of carbohydrate fuels in mammals. The enzymatic activity is regulated by a phosphorylation/dephosphorylation cycle. Phosphorylation of PDH by a specific pyruvate dehydrogenase kinase (PDK) results in inactivation. Multiple alternatively spliced transcript variants have been found for this gene. [provided by RefSeq, Jun 2013]

NFE2L2	nuclear factor, erythroid 2 like 2	This gene encodes a transcription factor which is a member of a small family of basic leucine zipper (bZIP) proteins. The encoded transcription factor regulates genes which contain antioxidant response elements (ARE) in their promoters; many of these genes encode proteins involved in response to injury and inflammation which includes the production of free radicals. Multiple transcript variants encoding different isoforms have been characterized for this gene. [provided by RefSeq, Sep 2015]
SF3B1	splicing factor 3b subunit 1	This gene encodes subunit 1 of the splicing factor 3b protein complex. Splicing factor 3b, together with splicing factor 3a and a 12S RNA unit, forms the U2 small nuclear ribonucleoproteins complex (U2 snRNP). The splicing factor 3b/3a complex binds pre-mRNA upstream of the intron's branch site in a sequence independent manner and may anchor the U2 snRNP to the pre-mRNA. Splicing factor 3b is also a component of the minor U12-type spliceosome. The carboxy-terminal two-thirds of subunit 1 have 22 non-identical, tandem HEAT repeats that form rod-like, helical structures. Alternative splicing results in multiple transcript variants encoding different isoforms. [provided by RefSeq, Jul 2008]
CASP8	caspase 8	This gene encodes a member of the cysteine-aspartic acid protease (caspase) family. Sequential activation of caspases plays a central role in the execution-phase of cell apoptosis. Caspases exist as inactive proenzymes composed of a prodomain, a large protease subunit, and a small protease subunit. Activation of caspases requires proteolytic processing at conserved internal aspartic residues to generate a heterodimeric enzyme consisting of the large and small subunits. This protein is involved in the programmed cell death induced by Fas and various apoptotic stimuli. The N-terminal FADD-like death effector domain of this protein suggests that it may interact with Fas-interacting protein FADD. This protein was detected in the insoluble fraction of the affected brain region from Huntington disease patients but not in those from normal controls, which implicated the role in neurodegenerative diseases. Many alternatively spliced transcript variants encoding different isoforms have been described, although not all variants have had their full-length sequences determined. [provided by RefSeq, Jul 2008]
IDH1	isocitrate dehydrogenase (NADP(+)) 1	Isocitrate dehydrogenases catalyze the oxidative decarboxylation of isocitrate to 2-oxoglutarate. These enzymes belong to two distinct subclasses, one of which utilizes NAD (+) as the electron acceptor and the other NADP(+). Five isocitrate dehydrogenases have been reported: three NAD (+)-dependent isocitrate dehydrogenases, which localize to the mitochondrial matrix, and two NADP(+)-dependent isocitrate dehydrogenases, one of which is mitochondrial and the other predominantly cytosolic. Each NADP (+)-dependent isozyme is a homodimer. The protein encoded by this gene is the NADP (+)-dependent isocitrate dehydrogenase found in the cytoplasm and peroxisomes. It contains the PTS-1 peroxisomal targeting signal sequence. The presence of this enzyme in peroxisomes suggests roles in the regeneration of NADPH for intraperoxisomal reductions, such as the conversion of 2, 4-dienoyl-CoAs to 3-enoyl-CoAs, as well as in peroxisomal reactions that consume 2-oxoglutarate, namely the alpha-hydroxylation of phytanic acid. The cytoplasmic enzyme serves a significant role in cytoplasmic NADPH production. Alternatively, spliced transcript variants encoding the same protein have been found for this gene. [provided by RefSeq, Sep 2013]
ERBB4	erb-b2 receptor tyrosine kinase 4	This gene is a member of the Tyr protein kinase family and the epidermal growth factor receptor subfamily. It encodes a single-pass type I membrane protein with multiple cysteine rich domains, a transmembrane domain, a tyrosine kinase domain, a phosphotidylinositol-3 kinase binding site and a PDZ domain binding motif. The protein binds to and is activated by neuregulin and other factors and induces a variety of cellular responses including mitogenesis and differentiation. Multiple proteolytic events allow for the release of a cytoplasmic fragment and an extracellular fragment. Mutations in this gene have been associated with cancer. Alternatively, spliced variants which encode different protein isoforms have been described; however, not all variants have been fully characterized. [provided by RefSeq, Jul 2008]
BARD1	BRCA1 associated RING domain 1	This gene encodes a protein which interacts with the N-terminal region of BRCA1. In addition to its ability to bind BRCA1 in vivo and in vitro, it shares homology with the 2 most conserved regions of BRCA1: the N-terminal RING motif and the C-terminal BRCT domain. The RING motif is a cysteine-rich sequence found in a variety of proteins that regulate cell growth, including the products of tumor suppressor genes and dominant protooncogenes. This protein also contains 3 tandem ankyrin repeats. The BARD1/BRCA1 interaction is disrupted by tumorigenic amino acid substitutions in BRCA1, implying that the formation of a stable complex between these proteins may be an essential aspect of BRCA1 tumor suppression. This protein may be the target of oncogenic mutations in breast or ovarian cancer. Multiple alternatively spliced transcript variants encoding different isoforms have been found for this gene. [provided by RefSeq, Sep 2013]
CUL3	cullin 3	This gene encodes a member of the cullin protein family. The encoded protein plays a critical role in the polyubiquitination and subsequent degradation of specific protein substrates as the core component and scaffold protein of an E3 ubiquitin ligase complex. Complexes including the encoded protein may also play a role in late endosome maturation. Mutations in this gene are a cause of type 2E pseudo hypoaldosteronism. Alternatively, spliced transcript variants encoding multiple isoforms have been observed for this gene. [provided by RefSeq, Mar 2012]
PDCD1	programmed cell death 1	Programmed cell death protein 1 (PDCD1) is an immune-inhibitory receptor expressed in activated T cells; it is involved in the regulation of T-cell functions, including those of effector CD8+ T cells. In addition, this protein can also promote the differentiation of CD4+ T cells into T regulatory cells. PDCD1 is expressed in many types of tumors including melanomas and has demonstrated to play a role in anti-tumor immunity. Moreover, this protein has been shown to be involved in safeguarding against autoimmunity, however, it can also contribute to the inhibition of effective anti-tumor and anti-microbial immunity. [provided by RefSeq, Aug 2020]
VHL	von Hippel-Lindau tumor suppressor	Von Hippel-Lindau syndrome (VHL) is a dominantly inherited familial cancer syndrome predisposing to a variety of malignant and benign tumors. A germline mutation of this gene is the basis of familial inheritance of VHL syndrome. The protein encoded by this gene is a component of the protein complex that includes elongin B, elongin C, and cullin-2, and possesses ubiquitin ligase E3 activity. This protein is involved in the ubiquitination and degradation of hypoxia-inducible-factor (HIF), which is a transcription factor that plays a central role in the regulation of gene expression by oxygen. RNA polymerase II subunit POLR2G/RPB7 is also reported to be a target of this protein. Alternatively, spliced transcript variants encoding distinct isoforms have been observed. [provided by RefSeq, Jul 2008]

PPARG	peroxisome proliferator activated receptor gamma	This gene encodes a member of the peroxisome proliferator-activated receptor (PPAR) subfamily of nuclear receptors. PPARs form heterodimers with retinoid X receptors (RXRs) and these heterodimers regulate transcription of various genes. Three subtypes of PPARs are known: PPAR-alpha, PPAR-delta, and PPAR-gamma. The protein encoded by this gene is PPAR-gamma and is a regulator of adipocyte differentiation. Additionally, PPAR-gamma has been implicated in the pathology of numerous diseases including obesity, diabetes, atherosclerosis and cancer. Alternatively, spliced transcript variants that encode different isoforms have been described. [provided by RefSeq, Jul 2008]
RAF1	Raf-1 proto-oncogene, serine/threonine kinase	This gene is the cellular homolog of viral raf gene (v-raf). The encoded protein is a MAP kinase kinase kinase (MAP3K), which functions downstream of the Ras family of membrane associated GTPases to which it binds directly. Once activated, the cellular RAF1 protein can phosphorylate to activate the dual specificity protein kinases MEK1 and MEK2, which in turn phosphorylate to activate the serine/threonine specific protein kinases, ERK1 and ERK2. Activated ERKs are pleiotropic effectors of cell physiology and play an important role in the control of gene expression involved in the cell division cycle, apoptosis, cell differentiation and cell migration. Mutations in this gene are associated with Noonan syndrome 5 and LEOPARD syndrome 2. [provided by RefSeq, Jul 2008]
TGFBR2	transforming growth factor beta receptor 2	The protein encoded by this gene is a transmembrane protein that has a protein kinase domain, forms a heterodimeric complex with TGF-beta receptor type-1, and binds TGF-beta. This receptor/ligand complex phosphorylates proteins, which then enter the nucleus and regulate the transcription of genes related to cell proliferation, cell cycle arrest, wound healing, immunosuppression, and tumorigenesis. Mutations in this gene have been associated with Marfan Syndrome, Loeys-Deitz Aortic Aneurysm Syndrome, and the development of various types of tumors. Alternatively, spliced transcript variants encoding different isoforms have been characterized. [provided by RefSeq, Aug 2017]
MLH1	mutL homolog 1	The protein encoded by this gene can heterodimerize with mismatch repair endonuclease PMS2 to form MutL alpha, part of the DNA mismatch repair system. When MutL alpha is bound by MutS beta and some accessory proteins, the PMS2 subunit of MutL alpha introduces a single strand break near DNA mismatches, providing an entry point for exonuclease degradation. The encoded protein is also involved in DNA damage signaling and can heterodimerize with DNA mismatch repair protein MLH3 to form MutL gamma, which is involved in meiosis. This gene was identified as a locus frequently mutated in hereditary nonpolyposis colon cancer (HNPCC). [provided by RefSeq, Aug 2017]
MYD88	MYD88 innate immune signal transduction adaptor	This gene encodes a cytosolic adapter protein that plays a central role in the innate and adaptive immune response. This protein functions as an essential signal transducer in the interleukin-1 and Toll-like receptor signaling pathways. These pathways regulate that activation of numerous proinflammatory genes. The encoded protein consists of an N-terminal death domain and a C-terminal Toll-interleukin1 receptor domain. Patients with defects in this gene have an increased susceptibility to pyogenic bacterial infections. Alternate splicing results in multiple transcript variants. [provided by RefSeq, Feb 2010]
CTNNB1	catenin beta 1	The protein encoded by this gene is part of a complex of proteins that constitute adherens junctions (AJs). AJs are necessary for the creation and maintenance of epithelial cell layers by regulating cell growth and adhesion between cells. The encoded protein also anchors the actin cytoskeleton and may be responsible for transmitting the contact inhibition signal that causes cells to stop dividing once the epithelial sheet is complete. Finally, this protein binds to the product of the APC gene, which is mutated in adenomatous polyposis of the colon. Mutations in this gene are a cause of colorectal cancer (CRC), pilomatrixoma (PTR), medulloblastoma (MDB), and ovarian cancer. Alternative splicing results in multiple transcript variants. [provided by RefSeq, Aug 2016]
SETD2	SET domain containing 2, histone lysine methyltransferase	Huntington's disease (HD), a neurodegenerative disorder characterized by loss of striatal neurons, is caused by an expansion of a polyglutamine tract in the HD protein huntingtin. This gene encodes a protein belonging to a class of huntingtin interacting proteins characterized by WW motifs. This protein is a histone methyltransferase that is specific for lysine-36 of histone H3, and methylation of this residue is associated with active chromatin. This protein also contains a novel transcriptional activation domain and has been found associated with hyperphosphorylated RNA polymerase II. [provided by RefSeq, Aug 2008]
MST1R	macrophage stimulating 1 receptor	This gene encodes a cell surface receptor for macrophage-stimulating protein (MSP) with tyrosine kinase activity. The mature form of this protein is a heterodimer of disulfide-linked alpha and beta subunits, generated by proteolytic cleavage of a single-chain precursor. The beta subunit undergoes tyrosine phosphorylation upon stimulation by MSP. This protein is expressed on the ciliated epithelia of the mucociliary transport apparatus of the lung, and together with MSP, thought to be involved in host defense. Alternative splicing generates multiple transcript variants encoding different isoforms that may undergo similar proteolytic processing. [provided by RefSeq, Jan 2016]
PARP3	poly(ADP-ribose) polymerase family member 3	The protein encoded by this gene belongs to the PARP family. These enzymes modify nuclear proteins by poly-ADP-ribosylation, which is required for DNA repair, regulation of apoptosis, and maintenance of genomic stability. This gene encodes the poly (ADP-ribosyl)transferase 3, which is preferentially localized to the daughter centriole throughout the cell cycle. Alternatively, spliced transcript variants encoding different isoforms have been identified. [provided by RefSeq, Jul 2008]
BAP1	BRCA1 associated protein 1	This gene belongs to the ubiquitin C-terminal hydrolase subfamily of deubiquitinating enzymes that are involved in the removal of ubiquitin from proteins. The encoded enzyme binds to the breast cancer type 1 susceptibility protein (BRCA1) via the RING finger domain of the latter and acts as a tumor suppressor. In addition, the enzyme may be involved in regulation of transcription, regulation of cell cycle and growth, response to DNA damage and chromatin dynamics. Germline mutations in this gene may be associated with tumor predisposition syndrome (TPDS), which involves increased risk of cancers including malignant mesothelioma, uveal melanoma and cutaneous melanoma. [provided by RefSeq, May 2013]
PBRM1	polybromo 1	This locus encodes a subunit of ATP-dependent chromatin-remodeling complexes. The encoded protein has been identified as in integral component of complexes necessary for ligand-dependent transcriptional activation by nuclear hormone receptors. Mutations at this locus have been associated with primary clear cell renal cell carcinoma. [provided by RefSeq, Feb 2012]
MITF	melanocyte inducing transcription factor	The protein encoded by this gene is a transcription factor that contains both basic helix-loop-helix and leucine zipper structural features. The encoded protein regulates melanocyte development and is responsible for pigment cell-specific transcription of the melanogenesis enzyme genes. Heterozygous mutations in this gene

		cause auditory-pigmentary syndromes, such as Waardenburg syndrome type 2 and Tietz syndrome. [provided by RefSeq, Aug 2017]
EPHA3	EPH receptor A3	This gene belongs to the ephrin receptor subfamily of the protein-tyrosine kinase family. EPH and EPH-related receptors have been implicated in mediating developmental events, particularly in the nervous system. Receptors in the EPH subfamily typically have a single kinase domain and an extracellular region containing a Cys-rich domain and 2 fibronectin type III repeats. The ephrin receptors are divided into 2 groups based on the similarity of their extracellular domain sequences and their affinities for binding ephrin-A and ephrin-B ligands. This gene encodes a protein that binds ephrin-A ligands. Two alternatively spliced transcript variants have been described for this gene. [provided by RefSeq, Jul 2008]
GSK3B	glycogen synthase kinase 3 beta	The protein encoded by this gene is a serine-threonine kinase belonging to the glycogen synthase kinase subfamily. It is a negative regulator of glucose homeostasis and is involved in energy metabolism, inflammation, ER-stress, mitochondrial dysfunction, and apoptotic pathways. Defects in this gene have been associated with Parkinson disease and Alzheimer disease. [provided by RefSeq, Aug 2017]
EPHB1	EPH receptor B1	Ephrin receptors and their ligands, the ephrin, mediate numerous developmental processes, particularly in the nervous system. Based on their structures and sequence relationships, ephrin are divided into the ephrin-A (EFNA) class, which are anchored to the membrane by a glycosylphosphatidylinositol linkage, and the ephrin-B (EFNB) class, which are transmembrane proteins. The Eph family of receptors are divided into 2 groups based on the similarity of their extracellular domain sequences and their affinities for binding ephrin-A and ephrin-B ligands. Ephrin receptors make up the largest subgroup of the receptor tyrosine kinase (RTK) family. The protein encoded by this gene is a receptor for ephrin-B family members. [provided by RefSeq, Jul 2008]
PIK3CB	phosphatidylinositol-4,5-bisphosphate 3-kinase catalytic subunit beta	This gene encodes an isoform of the catalytic subunit of phosphoinositide 3-kinase (PI3K). These kinases are important in signaling pathways involving receptors on the outer membrane of eukaryotic cells and are named for their catalytic subunit. The encoded protein is the catalytic subunit for PI3Kbeta (PI3KB). PI3KB has been shown to be part of the activation pathway in neutrophils which have bound immune complexes at sites of injury or infection. Alternative splicing results in multiple transcript variants. [provided by RefSeq, Dec 2011]
FOXL2	forkhead box L2	This gene encodes a fork head transcription factor. The protein contains a fork-head DNA-binding domain and may play a role in ovarian development and function. Expansion of a polyalanine repeat region and other mutations in this gene are a cause of blepharophimosis syndrome and premature ovarian failure 3. [provided by RefSeq, Jul 2016]
ATR	ATR serine/threonine kinase	The protein encoded by this gene is a serine/threonine kinase and DNA damage sensor, activating cell cycle checkpoint signaling upon DNA stress. The encoded protein can phosphorylate and activate several proteins involved in the inhibition of DNA replication and mitosis, and can promote DNA repair, recombination, and apoptosis. This protein is also important for fragile site stability and centrosome duplication. Defects in this gene are a cause of Seckel syndrome 1. [provided by RefSeq, Aug 2017]
TIPARP	TCDD inducible poly(ADP-ribose) polymerase	This gene encodes a member of the poly (ADP-ribose) polymerase superfamily. Studies of the mouse ortholog have shown that the encoded protein catalyzes histone poly (ADP-ribosyl) ation and may be involved in T-cell function. Alternative splicing results in multiple transcript variants. [provided by RefSeq, May 2010]
TERC	telomerase RNA component	Telomerase is a ribonucleoprotein polymerase that maintains telomere ends by addition of the telomere repeat TTAGGG. The enzyme consists of a protein component with reverse transcriptase activity, and an RNA component, encoded by this gene, that serves as a template for the telomere repeat. Telomerase expression plays a role in cellular senescence, as it is normally repressed in postnatal somatic cells resulting in progressive shortening of telomeres. Deregulation of telomerase expression in somatic cells may be involved in oncogenesis. Studies in mouse suggest that telomerase also participates in chromosomal repair, since de novo synthesis of telomere repeats may occur at double-stranded breaks. Mutations in this gene cause autosomal dominant dyskeratosis congenita and may also be associated with some cases of aplastic anemia. [provided by RefSeq, Jul 2008]
PRKCI	protein kinase C iota	This gene encodes a member of the protein kinase C (PKC) family of serine/threonine protein kinases. The PKC family comprises at least eight members, which are differentially expressed and are involved in a wide variety of cellular processes. This protein kinase is calcium-independent and phospholipid-dependent. It is not activated by phorbol esters or diacylglycerol. This kinase can be recruited to vesicle tubular clusters (VTCs) by direct interaction with the small GTPase RAB2, where this kinase phosphorylates glyceraldehyde-3-phosphate dehydrogenase (GAPD/GAPDH) and plays a role in microtubule dynamics in the early secretory pathway. This kinase is found to be necessary for BCL-ABL-mediated resistance to drug-induced apoptosis and therefore protects leukemia cells against drug-induced apoptosis. There is a single exon pseudogene mapped on chromosome X. [provided by RefSeq, Jul 2008]
PIK3CA	phosphatidylinositol-4,5-bisphosphate 3-kinase catalytic subunit alpha	Phosphatidylinositol 3-kinase is composed of an 85 kDa regulatory subunit and a 110 kDa catalytic subunit. The protein encoded by this gene represents the catalytic subunit, which uses ATP to phosphorylate PtdIns, PtdIns4P and PtdIns(4,5)P2. This gene has been found to be oncogenic and has been implicated in cervical cancers. A pseudogene of this gene has been defined on chromosome 22. [provided by RefSeq, Apr 2016]
SOX2	SRY-box transcription factor 2	This intron less gene encodes a member of the SRY-related HMG-box (SOX) family of transcription factors involved in the regulation of embryonic development and in the determination of cell fate. The product of this gene is required for stem-cell maintenance in the central nervous system, and also regulates gene expression in the stomach. Mutations in this gene have been associated with optic nerve hypoplasia and with syndromic microphthalmia, a severe form of structural eye malformation. This gene lies within an intron of another gene called SOX2 overlapping transcript (SOX2OT). [provided by RefSeq, Jul 2008]
KLHL6	kelch like family member 6	This gene encodes a member of the kelch-like (KLHL) family of proteins, which is involved in B-lymphocyte antigen receptor signaling and germinal-center B-cell maturation. The encoded protein contains an N-terminal broad-complex, tram track and bric a brac (BTB) domain that facilitates protein binding and dimerization, a BTB and C-terminal kelch (BACK) domain, and six C-terminal kelch repeat domains. Naturally occurring mutations in this gene are associated with chronic lymphocytic leukemia. Alternative splicing results in multiple transcript variants. [provided by RefSeq, Feb 2017]
MAP3K13	mitogen-activated protein kinase kinase kinase 13	The protein encoded by this gene is a member of serine/threonine protein kinase family. This kinase contains a dual leucine-zipper motif and has been shown to form dimers/oligomers through its leucine-zipper motif. This

		kinase can phosphorylate and activate MAPK8/JNK, MAP2K7/MKK7, which suggests a role in the JNK signaling pathway. [provided by RefSeq, Jul 2008]
ETV5	ETS variant transcription factor 5	Binds to DNA sequences containing the consensus nucleotide core sequence 5'-GGAA.-3'.
BCL6	BCL6 transcription repressor	The protein encoded by this gene is a zinc finger transcription factor and contains an N-terminal POZ domain. This protein acts as a sequence-specific repressor of transcription, and has been shown to modulate the transcription of STAT-dependent IL-4 responses of B cells. This protein can interact with a variety of POZ-containing proteins that function as transcription corepressors. This gene is found to be frequently translocated and hypermutated in diffuse large-cell lymphoma (DLCL), and may be involved in the pathogenesis of DLCL. Alternatively, spliced transcript variants encoding different protein isoforms have been found for this gene. [provided by RefSeq, Aug 2015]
FGF12	fibroblast growth factor 12	The protein encoded by this gene is a member of the fibroblast growth factor (FGF) family. FGF family members possess broad mitogenic and cell survival activities, and are involved in a variety of biological processes, including embryonic development, cell growth, morphogenesis, tissue repair, tumor growth, and invasion. This growth factor lacks the N-terminal signal sequence present in most of the FGF family members, but it contains clusters of basic residues that have been demonstrated to act as a nuclear localization signal. When transfected into mammalian cells, this protein accumulated in the nucleus, but was not secreted. The specific function of this gene has not yet been determined. [provided by RefSeq, Dec 2019]
FGFR3	fibroblast growth factor receptor 3	This gene encodes a member of the fibroblast growth factor receptor (FGFR) family, with its amino acid sequence being highly conserved between members and among divergent species. FGFR family members differ from one another in their ligand affinities and tissue distribution. A full-length representative protein would consist of an extracellular region, composed of three immunoglobulin-like domains, a single hydrophobic membrane-spanning segment and a cytoplasmic tyrosine kinase domain. The extracellular portion of the protein interacts with fibroblast growth factors, setting in motion a cascade of downstream signals, ultimately influencing mitogenesis and differentiation. This family member binds acidic and basic fibroblast growth hormone and plays a role in bone development and maintenance. Mutations in this gene lead to craniosynostosis and multiple types of skeletal dysplasia. [provided by RefSeq, Aug 2017]
WHSC1	nuclear receptor binding SET domain protein 2	This gene encodes a protein that contains four domains present in other developmental proteins: a PWWP domain, an HMG box, a SET domain, and a PHD-type zinc finger. It is expressed ubiquitously in early development. Wolf-Hirschhorn syndrome (WHS) is a malformation syndrome associated with a hemizygous deletion of the distal short arm of chromosome 4. This gene maps to the 165 kb WHS critical region and has also been involved in the chromosomal translocation t(4;14)(p16.3;q32.3) in multiple myelomas. Alternative splicing of this gene results in multiple transcript variants encoding different isoforms. Some transcript variants are nonsense-mediated mRNA (NMD) decay candidates, hence not represented as reference sequences. [provided by RefSeq, Jul 2008]
SLC34A2	solute carrier family 34 member 2	The protein encoded by this gene is a pH-sensitive sodium-dependent phosphate transporter. Phosphate uptake is increased at lower pH. Defects in this gene are a cause of pulmonary alveolar microlithiasis. Three transcript variants encoding two different isoforms have been found for this gene. [provided by RefSeq, May 2010]
PDGFRA	platelet derived growth factor receptor alpha	This gene encodes a cell surface tyrosine kinase receptor for members of the platelet-derived growth factor family. These growth factors are mitogens for cells of mesenchymal origin. The identity of the growth factor bound to a receptor monomer determines whether the functional receptor is a homodimer or a heterodimer, composed of both platelet-derived growth factor receptor alpha and beta polypeptides. Studies suggest that this gene plays a role in organ development, wound healing, and tumor progression. Mutations in this gene have been associated with idiopathic hyper eosinophilic syndrome, somatic and familial gastrointestinal stromal tumors, and a variety of other cancers. [provided by RefSeq, Mar 2012]
KIT	KIT proto-oncogene, receptor tyrosine kinase	This gene encodes a receptor tyrosine kinase. This gene was initially identified as a homolog of the feline sarcoma viral oncogene v-kit and is often referred to as proto-oncogene c-Kit. The canonical form of this glycosylated transmembrane protein has an N-terminal extracellular region with five immunoglobulin-like domains, a transmembrane region, and an intracellular tyrosine kinase domain at the C-terminus. Upon activation by its cytokine ligand, stem cell factor (SCF), this protein phosphorylates multiple intracellular proteins that play a role in in the proliferation, differentiation, migration and apoptosis of many cell types and thereby plays an important role in hematopoiesis, stem cell maintenance, gametogenesis, melanogenesis, and in mast cell development, migration and function. This protein can be a membrane-bound or soluble protein. Mutations in this gene are associated with gastrointestinal stromal tumors, mast cell disease, acute myelogenous leukemia, and piebaldism. Multiple transcript variants encoding different isoforms have been found for this gene. [provided by RefSeq, May 2020]
KDR	kinase insert domain receptor	Vascular endothelial growth factor (VEGF) is a major growth factor for endothelial cells. This gene encodes one of the two receptors of the VEGF. This receptor, known as kinase, insert domain receptor, is a type III receptor tyrosine kinase. It functions as the main mediator of VEGF-induced endothelial proliferation, survival, migration, tubular morphogenesis and sprouting. The signaling and trafficking of this receptor are regulated by multiple factors, including Rab GTPase, P2Y purine nucleotide receptor, integrin alphaVbeta3, T-cell protein tyrosine phosphatase, etc.. Mutations of this gene are implicated in infantile capillary hemangiomas. [provided by RefSeq, May 2009]
TET2	tet methylcytosine dioxygenase 2	The protein encoded by this gene is a methylcytosine dioxygenase that catalyzes the conversion of methylcytosine to 5-hydroxymethylcytosine. The encoded protein is involved in myelopoiesis, and defects in this gene have been associated with several myeloproliferative disorders. Two variants encoding different isoforms have been found for this gene. [provided by RefSeq, Mar 2011]
INPP4B	inositol polyphosphate-4-phosphatase type II B	INPP4B encodes the inositol polyphosphate 4-phosphatase type II, one of the enzymes involved in phosphatidylinositol signaling pathways. This enzyme removes the phosphate group at position 4 of the inositol ring from inositol 3,4-bisphosphate. There is limited data to suggest that the human type II enzyme is subject to alternative splicing, as has been established for the type I enzyme. [provided by RefSeq, Jul 2008]

FBXW7	F-box and WD repeat domain containing 7	This gene encodes a member of the F-box protein family which is characterized by an approximately 40 amino acid motifs, the F-box. The F-box proteins constitute one of the four subunits of ubiquitin protein ligase complex called SCFs (SKP1-cullin-F-box), which function in phosphorylation-dependent ubiquitination. The F-box proteins are divided into 3 classes: Fbws containing WD-40 domains, Fbls containing leucine-rich repeats, and Fbxs containing either different protein-protein interaction modules or no recognizable motifs. The protein encoded by this gene was previously referred to as FBX30 and belongs to the Fbws class; in addition to an F-box, this protein contains 7 tandem WD40 repeats. This protein binds directly to cyclin E and probably targets cyclin E for ubiquitin-mediated degradation. Mutations in this gene are detected in ovarian and breast cancer cell lines, implicating the gene's potential role in the pathogenesis of human cancers. Multiple transcript variants encoding different isoforms have been found for this gene. [provided by RefSeq, Mar 2012]
IRF2	interferon regulatory factor 2	IRF2 encodes interferon regulatory factor 2, a member of the interferon regulatory transcription factor (IRF) family. IRF2 competitively inhibits the IRF1-mediated transcriptional activation of interferons alpha and beta, and presumably other genes that employ IRF1 for transcription activation. However, IRF2 also functions as a transcriptional activator of histone H4. [provided by RefSeq, Jul 2008]
SDHA	succinate dehydrogenase complex flavoprotein subunit A	This gene encodes a major catalytic subunit of succinate-ubiquinone oxidoreductase, a complex of the mitochondrial respiratory chain. The complex is composed of four nuclear-encoded subunits and is localized in the mitochondrial inner membrane. Mutations in this gene have been associated with a form of mitochondrial respiratory chain deficiency known as Leigh Syndrome. A pseudogene has been identified on chromosome 3q29. Alternatively, spliced transcript variants encoding different isoforms have been found for this gene. [provided by RefSeq, Jun 2014]
TERT	telomerase reverse transcriptase	Telomerase is a ribonucleoprotein polymerase that maintains telomere ends by addition of the telomere repeat TTAGGG. The enzyme consists of a protein component with reverse transcriptase activity, encoded by this gene, and an RNA component which serves as a template for the telomere repeat. Telomerase expression plays a role in cellular senescence, as it is normally repressed in postnatal somatic cells resulting in progressive shortening of telomeres. Deregulation of telomerase expression in somatic cells may be involved in oncogenesis. Studies in mouse suggest that telomerase also participates in chromosomal repair, since de novo synthesis of telomere repeats may occur at double-stranded breaks. Alternatively spliced variants encoding different isoforms of telomerase reverse transcriptase have been identified; the full-length sequence of some variants has not been determined. Alternative splicing at this locus is thought to be one mechanism of regulation of telomerase activity. [provided by RefSeq, Jul 2008]
RICTOR	RPTOR independent companion of MTOR complex 2	RICTOR and MTOR (FRAP1; MIM 601231) are components of a protein complex that integrates nutrient- and growth factor-derived signals to regulate cell growth (Sarbassov et al., 2004 [PubMed 15268862]).[supplied by OMIM, Mar 2008]
FGF10	fibroblast growth factor 10	The protein encoded by this gene is a member of the fibroblast growth factor (FGF) family. FGF family members possess broad mitogenic and cell survival activities, and are involved in a variety of biological processes, including embryonic development, cell growth, morphogenesis, tissue repair, tumor growth and invasion. This protein exhibits mitogenic activity for keratinizing epidermal cells, but essentially no activity for fibroblasts, which is similar to the biological activity of FGF7. Studies of the mouse homolog of suggested that this gene is required for embryonic epidermal morphogenesis including brain development, lung morphogenesis, and initiation of lim bud formation. This gene is also implicated to be a primary factor in the process of wound healing. [provided by RefSeq, Jul 2008]
MAP3K1	mitogen-activated protein kinase kinase kinase 1	The protein encoded by this gene is a serine/threonine kinase and is part of some signal transduction cascades, including the ERK and JNK kinase pathways as well as the NF-kappa-B pathway. The encoded protein is activated by autophosphorylation and requires magnesium as a cofactor in phosphorylating other proteins. This protein has E3 ligase activity conferred by a plant homeodomain (PHD) in its N-terminus and phospho-kinase activity conferred by a kinase domain in its C-terminus. [provided by RefSeq, Mar 2012]
PIK3R1	phosphoinositide-3-kinase regulatory subunit 1	Phosphatidylinositol 3-kinase phosphorylates the inositol ring of phosphatidylinositol at the 3-prime position. The enzyme comprises a 110 kD catalytic subunit and a regulatory subunit of either 85, 55, or 50 kD. This gene encodes the 85 kD regulatory subunit. Phosphatidylinositol 3-kinase plays an important role in the metabolic actions of insulin, and a mutation in this gene has been associated with insulin resistance. Alternative splicing of this gene results in four transcript variants encoding different isoforms. [provided by RefSeq, Jun 2011]
MSH3	mutS homolog 3	The protein encoded by this gene forms a heterodimer with MSH2 to form MutS beta, part of the post-replicative DNA mismatch repair system. MutS beta initiates mismatch repair by binding to a mismatch and then forming a complex with MutL alpha heterodimer. This gene contains a polymorphic 9 bp tandem repeat sequence in the first exon. The repeat is present 6 times in the reference genome sequence and 3-7 repeats have been reported. Defects in this gene are a cause of susceptibility to endometrial cancer. [provided by RefSeq, Mar 2011]
APC	APC regulator of WNT signaling pathway	This gene encodes a tumor suppressor protein that acts as an antagonist of the Wnt signaling pathway. It is also involved in other processes including cell migration and adhesion, transcriptional activation, and apoptosis. Defects in this gene cause familial adenomatous polyposis (FAP), an autosomal dominant pre-malignant disease that usually progresses to malignancy. Mutations in the APC gene have been found to occur in most colorectal cancers. Disease-associated mutations tend to be clustered in a small region designated the mutation cluster region (MCR) and result in a truncated protein product. [provided by RefSeq, Dec 2019]
SNCAIP	synuclein alpha interacting protein	This gene encodes a protein containing several protein-protein interaction domains, including ankyrin-like repeats, a coiled-coil domain, and an ATP/GTP-binding motif. The encoded protein interacts with alpha-synuclein in neuronal tissue and may play a role in the formation of cytoplasmic inclusions and neurodegeneration. A mutation in this gene has been associated with Parkinson's disease. Alternative splicing results in multiple transcript variants. [provided by RefSeq, Apr 2015]
CTNNA1	catenin alpha 1	This gene encodes a member of the catenin family of proteins that play an important role in cell adhesion process by connecting cadherins located on the plasma membrane to the actin filaments inside the cell. The encoded mechanosensing protein contains three vinculin homology domains and undergoes conformational changes in response to cytoskeletal tension, resulting in the reconfiguration of cadherin-actin filament

		connections. Certain mutations in this gene cause butterfly-shaped pigment dystrophy. [provided by RefSeq, May 2016]
CSF1R	colony stimulating factor 1 receptor	The protein encoded by this gene is the receptor for colony stimulating factor 1, a cytokine which controls the production, differentiation, and function of macrophages. This receptor mediates most if not all of the biological effects of this cytokine. Ligand binding activates the receptor kinase through a process of oligomerization and transphosphorylation. The encoded protein is a tyrosine kinase transmembrane receptor and member of the CSF1/PDGF receptor family of tyrosine-protein kinases. Mutations in this gene have been associated with a predisposition to myeloid malignancy. The first intron of this gene contains a transcriptionally inactive ribosomal protein L7 processed pseudogene oriented in the opposite direction. Alternative splicing results in multiple transcript variants. Expression of a splice variant from an LTR promoter has been found in Hodgkin lymphoma (HL), HL cell lines and anaplastic large cell lymphoma. [provided by RefSeq, Mar 2017]
CD74	CD74 molecule	The protein encoded by this gene associates with class II major histocompatibility complex (MHC) and is an important chaperone that regulates antigen presentation for immune response. It also serves as cell surface receptor for the cytokine macrophage migration inhibitory factor (MIF) which, when bound to the encoded protein, initiates survival pathways and cell proliferation. This protein also interacts with amyloid precursor protein (APP) and suppresses the production of amyloid beta (Abeta). Multiple alternatively spliced transcript variants encoding different isoforms have been identified. [provided by RefSeq, Aug 2011]
PDGFRB	platelet derived growth factor receptor beta	The protein encoded by this gene is a cell surface tyrosine kinase receptor for members of the platelet-derived growth factor family. These growth factors are mitogens for cells of mesenchymal origin. The identity of the growth factor bound to a receptor monomer determines whether the functional receptor is a homodimer (PDGFB or PDGFD) or a heterodimer (PDGFA and PDGFB). This gene is essential for normal development of the cardiovascular system and aids in rearrangement of the actin cytoskeleton. This gene is flanked on chromosome 5 by the genes for granulocyte-macrophage colony-stimulating factor and macrophage-colony stimulating factor receptor; all three genes may be implicated in the 5-q syndrome. A translocation between chromosomes 5 and 12, that fuses this gene to that of the ETV6 gene, results in chronic myeloproliferative disorder with eosinophilia. [provided by RefSeq, Aug 2017]
GABRA6	gamma-aminobutyric acid type A receptor subunit alpha6	GABA is the major inhibitory neurotransmitter in the mammalian brain where it acts at GABA-A receptors, which are ligand-gated chloride channels. Chloride conductance of these channels can be modulated by agents such as benzodiazepines that bind to the GABA-A receptor. At least 16 distinct subunits of GABA-A receptors have been identified. [provided by RefSeq, Jul 2008]
NPM1	nucleophosmin 1	The protein encoded by this gene is involved in several cellular processes, including centrosome duplication, protein chaperoning, and cell proliferation. The encoded phosphoprotein shuttles between the nucleolus, nucleus, and cytoplasm, chaperoning ribosomal proteins and core histones from the nucleus to the cytoplasm. This protein is also known to sequester the tumor suppressor ARF in the nucleolus, protecting it from degradation until it is needed. Mutations in this gene are associated with acute myeloid leukemia. Dozens of pseudogenes of this gene have been identified. [provided by RefSeq, Aug 2017]
FGFR4	fibroblast growth factor receptor 4	The protein encoded by this gene is a tyrosine kinase and cell surface receptor for fibroblast growth factors. The encoded protein is involved in the regulation of several pathways, including cell proliferation, cell differentiation, cell migration, lipid metabolism, bile acid biosynthesis, vitamin D metabolism, glucose uptake, and phosphate homeostasis. This protein consists of an extracellular region, composed of three immunoglobulin-like domains, a single hydrophobic membrane-spanning segment, and a cytoplasmic tyrosine kinase domain. The extracellular portion interacts with fibroblast growth factors, setting in motion a cascade of downstream signals, ultimately influencing mitogenesis and differentiation. [provided by RefSeq, Aug 2017]
IRF4	interferon regulatory factor 4	The protein encoded by this gene belongs to the IRF (interferon regulatory factor) family of transcription factors, characterized by an unique tryptophan pentad repeat DNA-binding domain. The IRFs are important in the regulation of interferons in response to infection by virus, and in the regulation of interferon-inducible genes. This family member is lymphocyte specific and negatively regulates Toll-like-receptor (TLR) signaling that is central to the activation of innate and adaptive immune systems. A chromosomal translocation involving this gene and the IgH locus, t(6;14)(p25;q32), may be a cause of multiple myeloma. Alternatively spliced transcript variants have been found for this gene. [provided by RefSeq, Aug 2010]
DDR1	discoidin domain receptor tyrosine kinase 1	Receptor tyrosine kinases play a key role in the communication of cells with their microenvironment. These kinases are involved in the regulation of cell growth, differentiation and metabolism. The protein encoded by this gene belongs to a subfamily of tyrosine kinase receptors with homology to Dictyostelium discoideum protein discoidin I in their extracellular domain, and that are activated by various types of collagen. Expression of this protein is restricted to epithelial cells, particularly in the kidney, lung, gastrointestinal tract, and brain. In addition, it has been shown to be significantly overexpressed in several human tumors. Alternatively spliced transcript variants encoding different isoforms have been described for this gene. [provided by RefSeq, Feb 2011]
DAXX	death domain associated protein	This gene encodes a multifunctional protein that resides in multiple locations in the nucleus and in the cytoplasm. It interacts with a wide variety of proteins, such as apoptosis antigen Fas, centromere protein C, and transcription factor erythroblastosis virus E26 oncogene homolog 1. In the nucleus, the encoded protein functions as a potent transcription repressor that binds to sumoylated transcription factors. Its repression can be relieved by the sequestration of this protein into promyelocytic leukemia nuclear bodies or nucleoli. This protein also associates with centromeres in G2 phase. In the cytoplasm, the encoded protein may function to regulate apoptosis. The subcellular localization and function of this protein are modulated by post-translational modifications, including sumoylation, phosphorylation and polyubiquitination. Alternative splicing results in multiple transcript variants. [provided by RefSeq, Nov 2008]
CDKN1A	cyclin dependent kinase inhibitor 1A	This gene encodes a potent cyclin-dependent kinase inhibitor. The encoded protein binds to and inhibits the activity of cyclin-cyclin-dependent kinase2 or -cyclin-dependent kinase4 complexes, and thus functions as a regulator of cell cycle progression at G1. The expression of this gene is tightly controlled by the tumor suppressor protein p53, through which this protein mediates the p53-dependent cell cycle G1 phase arrest in response to a variety of stress stimuli. This protein can interact with proliferating cell nuclear antigen, a DNA polymerase accessory factor, and plays a regulatory role in S phase DNA replication and DNA damage repair.

		This protein was reported to be specifically cleaved by CASP3-like caspases, which thus leads to a dramatic activation of cyclin-dependent kinase2, and may be instrumental in the execution of apoptosis following caspase activation. Mice that lack this gene have the ability to regenerate damaged or missing tissue. Multiple alternatively spliced variants have been found for this gene. [provided by RefSeq, Sep 2015]
PIM1	Pim-1 proto-oncogene, serine/threonine kinase	The protein encoded by this gene belongs to the Ser/Thr protein kinase family, and PIM subfamily. This gene is expressed primarily in B-lymphoid and myeloid cell lines, and is overexpressed in hematopoietic malignancies and in prostate cancer. It plays a role in signal transduction in blood cells, contributing to both cell proliferation and survival, and thus provides a selective advantage in tumorigenesis. Both the human and orthologous mouse genes have been reported to encode two isoforms (with preferential cellular localization) resulting from the use of alternative in-frame translation initiation codons, the upstream non-AUG (CUG) and downstream AUG codons (PMIDs:16186805, 1825810).[provided by RefSeq, Aug 2011]
CCND3	cyclin D3	The protein encoded by this gene belongs to the highly conserved cyclin family, whose members are characterized by a dramatic periodicity in protein abundance through the cell cycle. Cyclins function as regulators of CDK kinases. Different cyclins exhibit distinct expression and degradation patterns which contribute to the temporal coordination of each mitotic event. This cyclin forms a complex with and functions as a regulatory subunit of CDK4 or CDK6, whose activtiy is required for cell cycle G1/S transition. This protein has been shown to interact with and be involved in the phosphorylation of tumor suppressor protein Rb. The CDK4 activity associated with this cyclin was reported to be necessary for cell cycle progression through G2 phase into mitosis after UV radiation. Several transcript variants encoding different isoforms have been found for this gene. [provided by RefSeq, Oct 2008]
VEGFA	vascular endothelial growth factor A	This gene is a member of the PDGF/VEGF growth factor family. It encodes a heparin-binding protein, which exists as a disulfide-linked homodimer. This growth factor induces proliferation and migration of vascular endothelial cells, and is essential for both physiological and pathological angiogenesis. Disruption of this gene in mice resulted in abnormal embryonic blood vessel formation. This gene is upregulated in many known tumors and its expression is correlated with tumor stage and progression. Elevated levels of this protein are found in patients with POEMS syndrome, also known as Crow-Fukase syndrome. Allelic variants of this gene have been associated with microvascular complications of diabetes 1 (MVCD1) and atherosclerosis. Alternatively spliced transcript variants encoding different isoforms have been described. There is also evidence for alternative translation initiation from upstream non-AUG (CUG) codons resulting in additional isoforms. A recent study showed that a C-terminally extended isoform is produced by use of an alternative in-frame translation termination codon via a stop codon readthrough mechanism, and that this isoform is antiangiogenic. Expression of some isoforms derived from the AUG start codon is regulated by a small upstream open reading frame, which is located within an internal ribosome entry site. The levels of VEGF are increased during infection with severe acute respiratory syndrome coronavirus 2 (SARS-CoV-2), thus promoting inflammation by facilitating recruitment of inflammatory cells, and by increasing the level of angiopoietin II (Ang II), one of two products of the SARS-CoV-2 binding target, angiotensin-converting enzyme 2 (ACE2). In turn, Ang II facilitates the elevation of VEGF, thus forming a vicious cycle in the release of inflammatory cytokines. [provided by RefSeq, Jun 2020]
PRDM1	PR/SET domain 1	This gene encodes a protein that acts as a repressor of beta-interferon gene expression. The protein binds specifically to the PRDI (positive regulatory domain I element) of the beta-IFN gene promoter. Transcription of this gene increases upon virus induction. Two alternatively spliced transcript variants that encode different isoforms have been reported. [provided by RefSeq, Jul 2008]
ROS1	ROS proto-oncogene 1, receptor tyrosine kinase	This proto-oncogene, highly-expressed in a variety of tumor cell lines, belongs to the sevenless subfamily of tyrosine kinase insulin receptor genes. The protein encoded by this gene is a type I integral membrane protein with tyrosine kinase activity. The protein may function as a growth or differentiation factor receptor. [provided by RefSeq, Jul 2008]
SGK1	serum/glucocorticoid regulated kinase 1	This gene encodes a serine/threonine protein kinase that plays an important role in cellular stress response. This kinase activates certain potassium, sodium, and chloride channels, suggesting an involvement in the regulation of processes such as cell survival, neuronal excitability, and renal sodium excretion. High levels of expression of this gene may contribute to conditions such as hypertension and diabetic nephropathy. Several alternatively spliced transcript variants encoding different isoforms have been noted for this gene. [provided by RefSeq, Jan 2009]
MYB	MYB proto-oncogene, transcription factor	This gene encodes a protein with three HTH DNA-binding domains that functions as a transcription regulator. This protein plays an essential role in the regulation of hematopoiesis. This gene may be aberrently expressed or rearranged or undergo translocation in leukemias and lymphomas, and is considered to be an oncogene. Alternative splicing results in multiple transcript variants. [provided by RefSeq, Jan 2016]
TNFAIP3	TNF alpha induced protein 3	This gene was identified as a gene whose expression is rapidly induced by the tumor necrosis factor (TNF). The protein encoded by this gene is a zinc finger protein and ubiqitin-editing enzyme, and has been shown to inhibit NF-kappa B activation as well as TNF-mediated apoptosis. The encoded protein, which has both ubiquitin ligase and deubiquitinase activities, is involved in the cytokine-mediated immune and inflammatory responses. Several transcript variants encoding the same protein have been found for this gene. [provided by RefSeq, Jul 2012]
ESR1	estrogen receptor 1	This gene encodes an estrogen receptor and ligand-activated transcription factor. The canonical protein contains an N-terminal ligand-independent transactivation domain, a central DNA binding domain, a hinge domain, and a C-terminal ligand-dependent transactivation domain. The protein localizes to the nucleus where it may form either a homodimer or a heterodimer with estrogen receptor 2. The protein encoded by this gene regulates the transcription of many estrogen-inducible genes that play a role in growth, metabolism, sexual development, gestation, and other reproductive functions and is expressed in many non-reproductive tissues. The receptor encoded by this gene plays a key role in breast cancer, endometrial cancer, and osteoporosis. This gene is reported to have dozens of transcript variants due to the use of alternate promoters and alternative splicing, however, the full-length nature of many of these variants remain uncertain. [provided by RefSeq, Jul 2020]
EZR	ezrin	The cytoplasmic peripheral membrane protein encoded by this gene functions as a protein-tyrosine kinase substrate in microvilli. As a member of the ERM protein family, this protein serves as an intermediate between

		the plasma membrane and the actin cytoskeleton. This protein plays a key role in cell surface structure adhesion, migration and organization, and it has been implicated in various human cancers. A pseudogene located on chromosome 3 has been identified for this gene. Alternatively spliced variants have also been described for this gene. [provided by RefSeq, Jul 2008]
PARK2	parkin RBR E3 ubiquitin protein ligase	The precise function of this gene is unknown; however, the encoded protein is a component of a multiprotein E3 ubiquitin ligase complex that mediates the targeting of substrate proteins for proteasomal degradation. Mutations in this gene are known to cause Parkinson disease and autosomal recessive juvenile Parkinson disease. Alternative splicing of this gene produces multiple transcript variants encoding distinct isoforms. Additional splice variants of this gene have been described but currently lack transcript support. [provided by RefSeq, Jul 2008]
QKI	QKI, KH domain containing RNA binding	The protein encoded by this gene is an RNA-binding protein that regulates pre-mRNA splicing, export of mRNAs from the nucleus, protein translation, and mRNA stability. The encoded protein is involved in myelinization and oligodendrocyte differentiation and may play a role in schizophrenia. Multiple transcript variants encoding different isoforms have been found for this gene. [provided by RefSeq, Jul 2014]
CARD11	caspase recruitment domain family member 11	The protein encoded by this gene belongs to the membrane-associated guanylate kinase (MAGUK) family, a class of proteins that functions as molecular scaffolds for the assembly of multiprotein complexes at specialized regions of the plasma membrane. This protein is also a member of the CARD protein family, which is defined by carrying a characteristic caspase-associated recruitment domain (CARD). This protein has a domain structure similar to that of CARD14 protein. The CARD domains of both proteins have been shown to specifically interact with BCL10, a protein known to function as a positive regulator of cell apoptosis and NF-kappaB activation. When expressed in cells, this protein activated NF-kappaB and induced the phosphorylation of BCL10. [provided by RefSeq, Jul 2008]
PMS2	PMS1 homolog 2, mismatch repair system component	The protein encoded by this gene is a key component of the mismatch repair system that functions to correct DNA mismatches and small insertions and deletions that can occur during DNA replication and homologous recombination. This protein forms heterodimers with the gene product of the mutL homolog 1 (MLH1) gene to form the MutL-alpha heterodimer. The MutL-alpha heterodimer possesses an endonucleolytic activity that is activated following recognition of mismatches and insertion/deletion loops by the MutS-alpha and MutS-beta heterodimers, and is necessary for removal of the mismatched DNA. There is a DQHA(X)2E(X)4E motif found at the C-terminus of the protein encoded by this gene that forms part of the active site of the nuclease. Mutations in this gene have been associated with hereditary nonpolyposis colorectal cancer (HNPCC; also known as Lynch syndrome) and Turcot syndrome. [provided by RefSeq, Apr 2016]
RAC1	Rac family small GTPase 1	The protein encoded by this gene is a GTPase which belongs to the RAS superfamily of small GTP-binding proteins. Members of this superfamily appear to regulate a diverse array of cellular events, including the control of cell growth, cytoskeletal reorganization, and the activation of protein kinases. Two transcript variants encoding different isoforms have been found for this gene. [provided by RefSeq, Mar 2009]
IKZF1	IKAROS family zinc finger 1	This gene encodes a transcription factor that belongs to the family of zinc-finger DNA-binding proteins associated with chromatin remodeling. The expression of this protein is restricted to the fetal and adult hemo-lymphopoietic system, and it functions as a regulator of lymphocyte differentiation. Several alternatively spliced transcript variants encoding different isoforms have been described for this gene. Most isoforms share a common C-terminal domain, which contains two zinc finger motifs that are required for hetero- or homo-dimerization, and for interactions with other proteins. The isoforms, however, differ in the number of N-terminal zinc finger motifs that bind DNA and in nuclear localization signal presence, resulting in members with and without DNA-binding properties. Only a few isoforms contain the requisite three or more N-terminal zinc motifs that confer high affinity binding to a specific core DNA sequence element in the promoters of target genes. The non-DNA-binding isoforms are largely found in the cytoplasm, and are thought to function as dominant-negative factors. Overexpression of some dominant-negative isoforms have been associated with B-cell malignancies, such as acute lymphoblastic leukemia (ALL). [provided by RefSeq, May 2014]
EGFR	epidermal growth factor receptor	The protein encoded by this gene is a transmembrane glycoprotein that is a member of the protein kinase superfamily. This protein is a receptor for members of the epidermal growth factor family. EGFR is a cell surface protein that binds to epidermal growth factor, thus inducing receptor dimerization and tyrosine autophosphorylation leading to cell proliferation. Mutations in this gene are associated with lung cancer. EGFR is a component of the cytokine storm which contributes to a severe form of Coronavirus Disease 2019 (COVID-19) resulting from infection with severe acute respiratory syndrome coronavirus-2 (SARS-CoV-2). [provided by RefSeq, Jul 2020]
HGF	hepatocyte growth factor	This gene encodes a protein that binds to the hepatocyte growth factor receptor to regulate cell growth, cell motility and morphogenesis in numerous cell and tissue types. Alternative splicing results in multiple transcript variants, at least one of which encodes a preproprotein that is proteolytically processed to generate alpha and beta chains, which form the mature heterodimer. This protein is secreted by mesenchymal cells and acts as a multi-functional cytokine on cells of mainly epithelial origin. This protein also plays a role in angiogenesis, tumorogenesis, and tissue regeneration. Although the encoded protein is a member of the peptidase S1 family of serine proteases, it lacks peptidase activity. Mutations in this gene are associated with nonsyndromic hearing loss. [provided by RefSeq, Nov 2015]
GRM3	glutamate metabotropic receptor 3	L-glutamate is the major excitatory neurotransmitter in the central nervous system and activates both ionotropic and metabotropic glutamate receptors. Glutamatergic neurotransmission is involved in most aspects of normal brain function and can be perturbed in many neuropathologic conditions. The metabotropic glutamate receptors are a family of G protein-coupled receptors, that have been divided into 3 groups on the basis of sequence homology, putative signal transduction mechanisms, and pharmacologic properties. Group I includes GRM1 and GRM5 and these receptors have been shown to activate phospholipase C. Group II includes GRM2 and GRM3 while Group III includes GRM4, GRM6, GRM7 and GRM8. Group II and III receptors are linked to the inhibition of the cyclic AMP cascade but differ in their agonist selectivities. [provided by RefSeq, Jul 2008]
CDK6	cyclin dependent kinase 6	The protein encoded by this gene is a member of the CMGC family of serine/threonine protein kinases. This kinase is a catalytic subunit of the protein kinase complex that is important for cell cycle G1 phase progression and G1/S transition. The activity of this kinase first appears in mid-G1 phase, which is controlled by the regulatory subunits including D-type cyclins and members of INK4 family of CDK inhibitors. This kinase, as

		well as CDK4, has been shown to phosphorylate, and thus regulate the activity of, tumor suppressor protein Rb. Altered expression of this gene has been observed in multiple human cancers. A mutation in this gene resulting in reduced cell proliferation, and impaired cell motility and polarity, and has been identified in patients with primary microcephaly. [provided by RefSeq, Aug 2017]
EPHB4	EPH receptor B4	Ephrin receptors and their ligands, the ephrins, mediate numerous developmental processes, particularly in the nervous system. Based on their structures and sequence relationships, ephrins are divided into the ephrin-A (EFNA) class, which are anchored to the membrane by a glycosylphosphatidylinositol linkage, and the ephrin-B (EFNB) class, which are transmembrane proteins. The Eph family of receptors are divided into 2 groups based on the similarity of their extracellular domain sequences and their affinities for binding ephrin-A and ephrin-B ligands. Ephrin receptors make up the largest subgroup of the receptor tyrosine kinase (RTK) family. The protein encoded by this gene binds to ephrin-B2 and plays an essential role in vascular development. [provided by RefSeq, Jul 2008]
MET	MET proto-oncogene, receptor tyrosine kinase	This gene encodes a member of the receptor tyrosine kinase family of proteins and the product of the proto-oncogene MET. The encoded preproprotein is proteolytically processed to generate alpha and beta subunits that are linked via disulfide bonds to form the mature receptor. Further processing of the beta subunit results in the formation of the M10 peptide, which has been shown to reduce lung fibrosis. Binding of its ligand, hepatocyte growth factor, induces dimerization and activation of the receptor, which plays a role in cellular survival, embryogenesis, and cellular migration and invasion. Mutations in this gene are associated with papillary renal cell carcinoma, hepatocellular carcinoma, and various head and neck cancers. Amplification and overexpression of this gene are also associated with multiple human cancers. [provided by RefSeq, May 2016]
SMO	smoothened, frizzled class receptor	The protein encoded by this gene is a G protein-coupled receptor that interacts with the patched protein, a receptor for hedgehog proteins. The encoded protein tranduces signals to other proteins after activation by a hedgehog protein/patched protein complex. [provided by RefSeq, Jul 2010]
BRAF	B-Raf proto-oncogene, serine/threonine kinase	This gene encodes a protein belonging to the RAF family of serine/threonine protein kinases. This protein plays a role in regulating the MAP kinase/ERK signaling pathway, which affects cell division, differentiation, and secretion. Mutations in this gene, most commonly the V600E mutation, are the most frequently identified cancer-causing mutations in melanoma, and have been identified in various other cancers as well, including non-Hodgkin lymphoma, colorectal cancer, thyroid carcinoma, non-small cell lung carcinoma, hairy cell leukemia and adenocarcinoma of lung. Mutations in this gene are also associated with cardiofaciocutaneous, Noonan, and Costello syndromes, which exhibit overlapping phenotypes. A pseudogene of this gene has been identified on the X chromosome. [provided by RefSeq, Aug 2017]
KEL	Kell metallo-endopeptidase (Kell blood group)	This gene encodes a type II transmembrane glycoprotein that is the highly polymorphic Kell blood group antigen. The Kell glycoprotein links via a single disulfide bond to the XK membrane protein that carries the Kx antigen. The encoded protein contains sequence and structural similarity to members of the neprilysin (M13) family of zinc endopeptidases. [provided by RefSeq, Jul 2008]
EZH2	enhancer of zeste 2 polycomb repressive complex 2 subunit	This gene encodes a member of the Polycomb-group (PcG) family. PcG family members form multimeric protein complexes, which are involved in maintaining the transcriptional repressive state of genes over successive cell generations. This protein associates with the embryonic ectoderm development protein, the VAV1 oncoprotein, and the X-linked nuclear protein. This protein may play a role in the hematopoietic and central nervous systems. Multiple alternatively splcied transcript variants encoding distinct isoforms have been identified for this gene. [provided by RefSeq, Feb 2011]
XRCC2	X-ray repair cross complementing 2	This gene encodes a member of the RecA/Rad51-related protein family that participates in homologous recombination to maintain chromosome stability and repair DNA damage. This gene is involved in the repair of DNA double-strand breaks by homologous recombination and it functionally complements Chinese hamster irs1, a repair-deficient mutant that exhibits hypersensitivity to a number of different DNA-damaging agents. [provided by RefSeq, Jul 2008]
GATA4	GATA binding protein 4	This gene encodes a member of the GATA family of zinc-finger transcription factors. Members of this family recognize the GATA motif which is present in the promoters of many genes. This protein is thought to regulate genes involved in embryogenesis and in myocardial differentiation and function, and is necessary for normal testicular development. Mutations in this gene have been associated with cardiac septal defects. Additionally, alterations in gene expression have been associated with several cancer types. Alternative splicing results in multiple transcript variants. [provided by RefSeq, Apr 2015]
PPP2R2A	protein phosphatase 2 regulatory subunit Balpha	The product of this gene belongs to the phosphatase 2 regulatory subunit B family. Protein phosphatase 2 is one of the four major Ser/Thr phosphatases, and it is implicated in the negative control of cell growth and division. It consists of a common heteromeric core enzyme, which is composed of a catalytic subunit and a constant regulatory subunit, that associates with a variety of regulatory subunits. The B regulatory subunit might modulate substrate selectivity and catalytic activity. This gene encodes an alpha isoform of the regulatory subunit B55 subfamily. Alternatively spliced transcript variants have been described. [provided by RefSeq, Apr 2010]
ZNF703	zinc finger protein 703	Transcriptional corepressor which does not bind directly to DNA and may regulate transcription through recruitment of histone deacetylases to gene promoters. Regulates cell adhesion, migration and proliferation. May be required for segmental gene expressi
WHSC1L1	nuclear receptor binding SET domain protein 3	This gene is related to the Wolf-Hirschhorn syndrome candidate-1 gene and encodes a protein with PWWP (proline-tryptophan-tryptophan-proline) domains. This protein methylates histone H3 at lysine residues 4 and 27, which represses gene transcription. Two alternatively spliced variants have been described. [provided by RefSeq, May 2015]
FGFR1	fibroblast growth factor receptor 1	The protein encoded by this gene is a member of the fibroblast growth factor receptor (FGFR) family, where amino acid sequence is highly conserved between members and throughout evolution. FGFR family members differ from one another in their ligand affinities and tissue distribution. A full-length representative protein consists of an extracellular region, composed of three immunoglobulin-like domains, a single hydrophobic membrane-spanning segment and a cytoplasmic tyrosine kinase domain. The extracellular portion of the protein interacts with fibroblast growth factors, setting in motion a cascade of downstream signals, ultimately influencing mitogenesis and differentiation. This particular family member binds both acidic and basic fibroblast growth factors and is involved in limb induction. Mutations in this gene have been associated with

		Pfeiffer syndrome, Jackson-Weiss syndrome, Antley-Bixler syndrome, osteoglophonic dysplasia, and autosomal dominant Kallmann syndrome 2. Chromosomal aberrations involving this gene are associated with stem cell myeloproliferative disorder and stem cell leukemia lymphoma syndrome. Alternatively spliced variants which encode different protein isoforms have been described; however, not all variants have been fully characterized. [provided by RefSeq, Jul 2008]
LYN	LYN proto-oncogene, Src family tyrosine kinase	This gene encodes a tyrosine protein kinase, which maybe involved in the regulation of mast cell degranulation, and erythroid differentiation. Alternatively spliced transcript variants encoding different isoforms have been found for this gene. [provided by RefSeq, Jul 2011]
NBN	nibrin	Mutations in this gene are associated with Nijmegen breakage syndrome, an autosomal recessive chromosomal instability syndrome characterized by microcephaly, growth retardation, immunodeficiency, and cancer predisposition. The encoded protein is a member of the MRE11/RAD50 double-strand break repair complex which consists of 5 proteins. This gene product is thought to be involved in DNA double-strand break repair and DNA damage-induced checkpoint activation. [provided by RefSeq, Jul 2008]
RSPO2	R-spondin 2	This gene encodes a member of the R-spondin family of proteins. These proteins are secreted ligands of leucine-rich repeat containing G protein-coupled receptors that enhance Wnt signaling through the inhibition of ubiquitin E3 ligases. A chromosomal translocation including this locus that results in the formation of a gene fusion has been identified in multiple human cancers. Alternative splicing results in multiple transcript variants. [provided by RefSeq, Dec 2015]
RAD21	RAD21 cohesin complex component	The protein encoded by this gene is highly similar to the gene product of Schizosaccharomyces pombe rad21, a gene involved in the repair of DNA double-strand breaks, as well as in chromatid cohesion during mitosis. This protein is a nuclear phospho-protein, which becomes hyperphosphorylated in cell cycle M phase. The highly regulated association of this protein with mitotic chromatin specifically at the centromere region suggests its role in sister chromatid cohesion in mitotic cells. [provided by RefSeq, Jul 2008]
MYC	MYC proto-oncogene, bHLH transcription factor	This gene is a proto-oncogene and encodes a nuclear phosphoprotein that plays a role in cell cycle progression, apoptosis and cellular transformation. The encoded protein forms a heterodimer with the related transcription factor MAX. This complex binds to the E box DNA consensus sequence and regulates the transcription of specific target genes. Amplification of this gene is frequently observed in numerous human cancers. Translocations involving this gene are associated with Burkitt lymphoma and multiple myeloma in human patients. There is evidence to show that translation initiates both from an upstream, in-frame non-AUG (CUG) and a downstream AUG start site, resulting in the production of two isoforms with distinct N-termini. [provided by RefSeq, Aug 2017]
JAK2	Janus kinase 2	This gene encodes a non-receptor tyrosine kinase that plays a central role in cytokine and growth factor signalling. The primary isoform of this protein has an N-terminal FERM domain that is required for erythropoietin receptor association, an SH2 domain that binds STAT transcription factors, a pseudokinase domain and a C-terminal tyrosine kinase domain. Cytokine binding induces autophosphorylation and activation of this kinase. This kinase then recruits and phosphorylates signal transducer and activator of transcription (STAT) proteins. Growth factors like TGF-beta 1 also induce phosphorylation and activation of this kinase and translocation of downstream STAT proteins to the nucleus where they influence gene transcription. Mutations in this gene are associated with numerous inflammatory diseases and malignancies. This gene is a downstream target of the pleiotropic cytokine IL6 that is produced by B cells, T cells, dendritic cells and macrophages to produce an immune response or inflammation. Disregulation of the IL6/JAK2/STAT3 signalling pathways produces increased cellular proliferation and myeloproliferative neoplasms of hematopoietic stem cells. A nonsynonymous mutation in the pseudokinase domain of this gene disrupts the domains inhibitory effect and results in constitutive tyrosine phosphorylation activity and hypersensitivity to cytokine signalling. This gene and the IL6/JAK2/STAT3 signalling pathway is a therapeutic target for the treatment of excessive inflammatory responses to viral infections. Alternative splicing results in multiple transcript variants encoding distinct isoforms. [provided by RefSeq, Jul 2020]
CD274	CD274 molecule	This gene encodes an immune inhibitory receptor ligand that is expressed by hematopoietic and non-hematopoietic cells, such as T cells and B cells and various types of tumor cells. The encoded protein is a type I transmembrane protein that has immunoglobulin V-like and C-like domains. Interaction of this ligand with its receptor inhibits T-cell activation and cytokine production. During infection or inflammation of normal tissue, this interaction is important for preventing autoimmunity by maintaining homeostasis of the immune response. In tumor microenvironments, this interaction provides an immune escape for tumor cells through cytotoxic T-cell inactivation. Expression of this gene in tumor cells is considered to be prognostic in many types of human malignancies, including colon cancer and renal cell carcinoma. Alternative splicing results in multiple transcript variants. [provided by RefSeq, Sep 2015]
PDCD1LG2	programmed cell death 1 ligand 2	Involved in the costimulatory signal, essential for T-cell proliferation and IFNG production in a PDCD1-independent manner. Interaction with PDCD1 inhibits T-cell proliferation by blocking cell cycle progression and cytokine production (By similarity).
MTAP	methylthioadenosine phosphorylase	This gene encodes an enzyme that plays a major role in polyamine metabolism and is important for the salvage of both adenine and methionine. The encoded enzyme is deficient in many cancers because this gene and the tumor suppressor p16 gene are co-deleted. Multiple alternatively spliced transcript variants have been described for this gene, but their full-length natures remain unknown. [provided by RefSeq, Jul 2008]
CDKN2A	cyclin dependent kinase inhibitor 2A	This gene generates several transcript variants which differ in their first exons. At least three alternatively spliced variants encoding distinct proteins have been reported, two of which encode structurally related isoforms known to function as inhibitors of CDK4 kinase. The remaining transcript includes an alternate first exon located 20 Kb upstream of the remainder of the gene; this transcript contains an alternate open reading frame (ARF) that specifies a protein which is structurally unrelated to the products of the other variants. This ARF product functions as a stabilizer of the tumor suppressor protein p53 as it can interact with, and sequester, the E3 ubiquitin-protein ligase MDM2, a protein responsible for the degradation of p53. In spite of the structural and functional differences, the CDK inhibitor isoforms and the ARF product encoded by this gene, through the regulatory roles of CDK4 and p53 in cell cycle G1 progression, share a common functionality in cell cycle G1 control. This gene is frequently mutated or deleted in a wide variety of tumors, and is known to be an important tumor suppressor gene. [provided by RefSeq, Sep 2012]

CDKN2B	cyclin dependent kinase inhibitor 2B	This gene lies adjacent to the tumor suppressor gene CDKN2A in a region that is frequently mutated and deleted in a wide variety of tumors. This gene encodes a cyclin-dependent kinase inhibitor, which forms a complex with CDK4 or CDK6, and prevents the activation of the CDK kinases, thus the encoded protein functions as a cell growth regulator that controls cell cycle G1 progression. The expression of this gene was found to be dramatically induced by TGF beta, which suggested its role in the TGF beta induced growth inhibition. Two alternatively spliced transcript variants of this gene, which encode distinct proteins, have been reported. [provided by RefSeq, Jul 2008]
TEK	TEK receptor tyrosine kinase	This gene encodes a receptor that belongs to the protein tyrosine kinase Tie2 family. The encoded protein possesses a unique extracellular region that contains two immunoglobulin-like domains, three epidermal growth factor (EGF)-like domains and three fibronectin type III repeats. The ligand angiopoietin-1 binds to this receptor and mediates a signaling pathway that functions in embryonic vascular development. Mutations in this gene are associated with inherited venous malformations of the skin and mucous membranes. Alternative splicing results in multiple transcript variants. Additional alternatively spliced transcript variants of this gene have been described, but their full-length nature is not known. [provided by RefSeq, Feb 2014]
FANCG	FA complementation group G	The Fanconi anemia complementation group (FANC) currently includes FANCA, FANCB, FANCC, FANCD1 (also called BRCA2), FANCD2, FANCE, FANCF, FANCG, FANCI, FANCJ (also called BRIP1), FANCL, FANCM and FANCN (also called PALB2). The previously defined group FANCH is the same as FANCA. Fanconi anemia is a genetically heterogeneous recessive disorder characterized by cytogenetic instability, hypersensitivity to DNA crosslinking agents, increased chromosomal breakage, and defective DNA repair. The members of the Fanconi anemia complementation group do not share sequence similarity; they are related by their assembly into a common nuclear protein complex. This gene encodes the protein for complementation group G. [provided by RefSeq, Jul 2008]
PAX5	paired box 5	This gene encodes a member of the paired box (PAX) family of transcription factors. The central feature of this gene family is a novel, highly conserved DNA-binding motif, known as the paired box. Paired box transcription factors are important regulators in early development, and alterations in the expression of their genes are thought to contribute to neoplastic transformation. This gene encodes the B-cell lineage specific activator protein that is expressed at early, but not late stages of B-cell differentiation. Its expression has also been detected in developing CNS and testis and so the encoded protein may also play a role in neural development and spermatogenesis. This gene is located at 9p13, which is involved in t(9;14)(p13;q32) translocations recurring in small lymphocytic lymphomas of the plasmacytoid subtype, and in derived large-cell lymphomas. This translocation brings the potent E-mu enhancer of the IgH gene into close proximity of the PAX5 promoter, suggesting that the deregulation of transcription of this gene contributes to the pathogenesis of these lymphomas. Alternative splicing results in multiple transcript variants encoding different isoforms. [provided by RefSeq, Jul 2013]
GNAQ	G protein subunit alpha q	This locus encodes a guanine nucleotide-binding protein. The encoded protein, an alpha subunit in the Gq class, couples a seven-transmembrane domain receptor to activation of phospolipase C-beta. Mutations at this locus have been associated with problems in platelet activation and aggregation. A related pseudogene exists on chromosome 2.[provided by RefSeq, Nov 2010]
NTRK2	neurotrophic receptor tyrosine kinase 2	This gene encodes a member of the neurotrophic tyrosine receptor kinase (NTRK) family. This kinase is a membrane-bound receptor that, upon neurotrophin binding, phosphorylates itself and members of the MAPK pathway. Signalling through this kinase leads to cell differentiation. Mutations in this gene have been associated with obesity and mood disorders. Alternative splicing results in multiple transcript variants. [provided by RefSeq, May 2014]
SYK	spleen associated tyrosine kinase	This gene encodes a member of the family of non-receptor type Tyr protein kinases. This protein is widely expressed in hematopoietic cells and is involved in coupling activated immunoreceptors to downstream signaling events that mediate diverse cellular responses, including proliferation, differentiation, and phagocytosis. It is thought to be a modulator of epithelial cell growth and a potential tumour suppressor in human breast carcinomas. Alternatively spliced transcript variants encoding different isoforms have been found for this gene. [provided by RefSeq, Mar 2010]
FANCC	FA complementation group C	The Fanconi anemia complementation group (FANC) currently includes FANCA, FANCB, FANCC, FANCD1 (also called BRCA2), FANCD2, FANCE, FANCF, FANCG, FANCI, FANCJ (also called BRIP1), FANCL, FANCM and FANCN (also called PALB2). The previously defined group FANCH is the same as FANCA. Fanconi anemia is a genetically heterogeneous recessive disorder characterized by cytogenetic instability, hypersensitivity to DNA crosslinking agents, increased chromosomal breakage, and defective DNA repair. The members of the Fanconi anemia complementation group do not share sequence similarity; they are related by their assembly into a common nuclear protein complex. This gene encodes the protein for complementation group C. [provided by RefSeq, Jul 2008]
PTCH1	patched 1	This gene encodes a member of the patched family of proteins and a component of the hedgehog signaling pathway. Hedgehog signaling is important in embryonic development and tumorigenesis. The encoded protein is the receptor for the secreted hedgehog ligands, which include sonic hedgehog, indian hedgehog and desert hedgehog. Following binding by one of the hedgehog ligands, the encoded protein is trafficked away from the primary cilium, relieving inhibition of the G-protein-coupled receptor smoothened, which results in activation of downstream signaling. Mutations of this gene have been associated with basal cell nevus syndrome and holoprosencephaly. [provided by RefSeq, Aug 2017]
ABL1	ABL proto-oncogene 1, non-receptor tyrosine kinase	This gene is a protooncogene that encodes a protein tyrosine kinase involved in a variety of cellular processes, including cell division, adhesion, differentiation, and response to stress. The activity of the protein is negatively regulated by its SH3 domain, whereby deletion of the region encoding this domain results in an oncogene. The ubiquitously expressed protein has DNA-binding activity that is regulated by CDC2-mediated phosphorylation, suggesting a cell cycle function. This gene has been found fused to a variety of translocation partner genes in various leukemias, most notably the t(9;22) translocation that results in a fusion with the 5' end of the breakpoint cluster region gene (BCR; MIM:151410). Alternative splicing of this gene results in two transcript variants, which contain alternative first exons that are spliced to the remaining common exons. [provided by RefSeq, Aug 2014]

TSC1	TSC complex subunit 1	This gene is a tumor suppressor gene that encodes the growth inhibitory protein hamartin. The encoded protein interacts with and stabilizes the GTPase activating protein tuberin. This hamartin-tuberin complex negatively regulates mammalian target of rapamycin complex 1 (mTORC1) signalling which is a major regulator of anabolic cell growth. This protein also functions as a co-chaperone for Hsp90 that inhibits its ATPase activity. This protein functions as a facilitator of Hsp90-mediated folding of kinase and non-kinase clients, including Tsc2 and thereby preventing their ubiquitination and proteasomal degradation. Mutations in this gene have been associated with tuberous sclerosis. [provided by RefSeq, Apr 2018]
NOTCH1	notch receptor 1	This gene encodes a member of the NOTCH family of proteins. Members of this Type I transmembrane protein family share structural characteristics including an extracellular domain consisting of multiple epidermal growth factor-like (EGF) repeats, and an intracellular domain consisting of multiple different domain types. Notch signaling is an evolutionarily conserved intercellular signaling pathway that regulates interactions between physically adjacent cells through binding of Notch family receptors to their cognate ligands. The encoded preproprotein is proteolytically processed in the trans-Golgi network to generate two polypeptide chains that heterodimerize to form the mature cell-surface receptor. This receptor plays a role in the development of numerous cell and tissue types. Mutations in this gene are associated with aortic valve disease, Adams-Oliver syndrome, T-cell acute lymphoblastic leukemia, chronic lymphocytic leukemia, and head and neck squamous cell carcinoma. [provided by RefSeq, Jan 2016]
GATA3	GATA binding protein 3	This gene encodes a protein which belongs to the GATA family of transcription factors. The protein contains two GATA-type zinc fingers and is an important regulator of T-cell development and plays an important role in endothelial cell biology. Defects in this gene are the cause of hypoparathyroidism with sensorineural deafness and renal dysplasia. [provided by RefSeq, Nov 2009]
RET	ret proto-oncogene	This gene encodes a transmembrane receptor and member of the tyrosine protein kinase family of proteins. Binding of ligands such as GDNF (glial cell-line derived neurotrophic factor) and other related proteins to the encoded receptor stimulates receptor dimerization and activation of downstream signaling pathways that play a role in cell differentiation, growth, migration and survival. The encoded receptor is important in development of the nervous system, and the development of organs and tissues derived from the neural crest. This proto-oncogene can undergo oncogenic activation through both cytogenetic rearrangement and activating point mutations. Mutations in this gene are associated with Hirschsprung disease and central hypoventilation syndrome and have been identified in patients with renal agenesis. [provided by RefSeq, Sep 2017]
PTEN	phosphatase and tensin homolog	This gene was identified as a tumor suppressor that is mutated in a large number of cancers at high frequency. The protein encoded by this gene is a phosphatidylinositol-3,4,5-trisphosphate 3-phosphatase. It contains a tensin like domain as well as a catalytic domain similar to that of the dual specificity protein tyrosine phosphatases. Unlike most of the protein tyrosine phosphatases, this protein preferentially dephosphorylates phosphoinositide substrates. It negatively regulates intracellular levels of phosphatidylinositol-3,4,5-trisphosphate in cells and functions as a tumor suppressor by negatively regulating AKT/PKB signaling pathway. The use of a non-canonical (CUG) upstream initiation site produces a longer isoform that initiates translation with a leucine, and is thought to be preferentially associated with the mitochondrial inner membrane. This longer isoform may help regulate energy metabolism in the mitochondria. A pseudogene of this gene is found on chromosome 9. Alternative splicing and the use of multiple translation start codons results in multiple transcript variants encoding different isoforms. [provided by RefSeq, Feb 2015]
FAS	Fas cell surface death receptor	The protein encoded by this gene is a member of the TNF-receptor superfamily. This receptor contains a death domain. It has been shown to play a central role in the physiological regulation of programmed cell death, and has been implicated in the pathogenesis of various malignancies and diseases of the immune system. The interaction of this receptor with its ligand allows the formation of a death-inducing signaling complex that includes Fas-associated death domain protein (FADD), caspase 8, and caspase 10. The autoproteolytic processing of the caspases in the complex triggers a downstream caspase cascade, and leads to apoptosis. This receptor has been also shown to activate NF-kappaB, MAPK3/ERK1, and MAPK8/JNK, and is found to be involved in transducing the proliferating signals in normal diploid fibroblast and T cells. Several alternatively spliced transcript variants have been described, some of which are candidates for nonsense-mediated mRNA decay (NMD). The isoforms lacking the transmembrane domain may negatively regulate the apoptosis mediated by the full length isoform. [provided by RefSeq, Mar 2011]
SUFU	SUFU negative regulator of hedgehog signaling	The Hedgehog signaling pathway plays an important role in early human development. The pathway is a signaling cascade that plays a role in pattern formation and cellular proliferation during development. This gene encodes a negative regulator of the hedgehog signaling pathway. Defects in this gene are a cause of medulloblastoma. Alternative splicing results in multiple transcript variants.[provided by RefSeq, May 2010]
CYP17A1	cytochrome P450 family 17 subfamily A member 1	This gene encodes a member of the cytochrome P450 superfamily of enzymes. The cytochrome P450 proteins are monooxygenases which catalyze many reactions involved in drug metabolism and synthesis of cholesterol, steroids and other lipids. This protein localizes to the endoplasmic reticulum. It has both 17alpha-hydroxylase and 17,20-lyase activities and is a key enzyme in the steroidogenic pathway that produces progestins, mineralocorticoids, glucocorticoids, androgens, and estrogens. Mutations in this gene are associated with isolated steroid-17 alpha-hydroxylase deficiency, 17-alpha-hydroxylase/17,20-lyase deficiency, pseudohermaphroditism, and adrenal hyperplasia. [provided by RefSeq, Jul 2008]
NT5C2	5'-nucleotidase, cytosolic II	This gene encodes a hydrolase that serves as an important role in cellular purine metabolism by acting primarily on inosine 5'-monophosphate and other purine nucleotides. [provided by RefSeq, Oct 2011]
FGFR2	fibroblast growth factor receptor 2	The protein encoded by this gene is a member of the fibroblast growth factor receptor family, where amino acid sequence is highly conserved between members and throughout evolution. FGFR family members differ from one another in their ligand affinities and tissue distribution. A full-length representative protein consists of an extracellular region, composed of three immunoglobulin-like domains, a single hydrophobic membrane-spanning segment and a cytoplasmic tyrosine kinase domain. The extracellular portion of the protein interacts with fibroblast growth factors, setting in motion a cascade of downstream signals, ultimately influencing mitogenesis and differentiation. This particular family member is a high-affinity receptor for acidic, basic and/or keratinocyte growth factor, depending on the isoform. Mutations in this gene are associated with Crouzon syndrome, Pfeiffer syndrome, Craniosynostosis, Apert syndrome, Jackson-Weiss syndrome, Beare-Stevenson cutis gyrata syndrome, Saethre-Chotzen syndrome, and syndromic craniosynostosis. Multiple

		alternatively spliced transcript variants encoding different isoforms have been noted for this gene. [provided by RefSeq, Jan 2009]
HRAS	HRas proto-oncogene, GTPase	This gene belongs to the Ras oncogene family, whose members are related to the transforming genes of mammalian sarcoma retroviruses. The products encoded by these genes function in signal transduction pathways. These proteins can bind GTP and GDP, and they have intrinsic GTPase activity. This protein undergoes a continuous cycle of de- and re-palmitoylation, which regulates its rapid exchange between the plasma membrane and the Golgi apparatus. Mutations in this gene cause Costello syndrome, a disease characterized by increased growth at the prenatal stage, growth deficiency at the postnatal stage, predisposition to tumor formation, cognitive disability, skin and musculoskeletal abnormalities, distinctive facial appearance and cardiovascular abnormalities. Defects in this gene are implicated in a variety of cancers, including bladder cancer, follicular thyroid cancer, and oral squamous cell carcinoma. Multiple transcript variants, which encode different isoforms, have been identified for this gene. [provided by RefSeq, Jul 2008]
WT1	WT1 transcription factor	This gene encodes a transcription factor that contains four zinc-finger motifs at the C-terminus and a proline/glutamine-rich DNA-binding domain at the N-terminus. It has an essential role in the normal development of the urogenital system, and it is mutated in a small subset of patients with Wilms tumor. This gene exhibits complex tissue-specific and polymorphic imprinting pattern, with biallelic, and monoallelic expression from the maternal and paternal alleles in different tissues. Multiple transcript variants have been described. In several variants, there is evidence for the use of a non-AUG (CUG) translation initiation codon upstream of, and in-frame with the first AUG. Authors of PMID:7926762 also provide evidence that WT1 mRNA undergoes RNA editing in human and rat, and that this process is tissue-restricted and developmentally regulated. [provided by RefSeq, Mar 2015]
MEN1	menin 1	This gene encodes menin, a tumor suppressor associated with a syndrome known as multiple endocrine neoplasia type 1. Menin is a scaffold protein that functions in histone modification and epigenetic gene regulation. It is thought to regulate several pathways and processes by altering chromatin structure through the modification of histones. [provided by RefSeq, May 2019]
CCND1	cyclin D1	The protein encoded by this gene belongs to the highly conserved cyclin family, whose members are characterized by a dramatic periodicity in protein abundance throughout the cell cycle. Cyclins function as regulators of CDK kinases. Different cyclins exhibit distinct expression and degradation patterns which contribute to the temporal coordination of each mitotic event. This cyclin forms a complex with and functions as a regulatory subunit of CDK4 or CDK6, whose activity is required for cell cycle G1/S transition. This protein has been shown to interact with tumor suppressor protein Rb and the expression of this gene is regulated positively by Rb. Mutations, amplification and overexpression of this gene, which alters cell cycle progression, are observed frequently in a variety of human cancers. [provided by RefSeq, Dec 2019]
FGF19	fibroblast growth factor 19	The protein encoded by this gene is a member of the fibroblast growth factor (FGF) family. FGF family members possess broad mitogenic and cell survival activities, and are involved in a variety of biological processes including embryonic development cell growth, morphogenesis, tissue repair, tumor growth and invasion. This growth factor is a high affinity, heparin dependent ligand for FGFR4. Expression of this gene was detected only in fetal but not adult brain tissue. Synergistic interaction of the chick homolog and Wnt-8c has been shown to be required for initiation of inner ear development. [provided by RefSeq, Jul 2008]
FGF4	fibroblast growth factor 4	The protein encoded by this gene is a member of the fibroblast growth factor (FGF) family. FGF family members possess broad mitogenic and cell survival activities and are involved in a variety of biological processes including embryonic development, cell growth, morphogenesis, tissue repair, tumor growth and invasion. This gene was identified by its oncogenic transforming activity. This gene and FGF3, another oncogenic growth factor, are located closely on chromosome 11. Co-amplification of both genes was found in various kinds of human tumors. Studies on the mouse homolog suggested a function in bone morphogenesis and limb development through the sonic hedgehog (SHH) signaling pathway. [provided by RefSeq, Jul 2008]
FGF3	fibroblast growth factor 3	The protein encoded by this gene is a member of the fibroblast growth factor (FGF) family. FGF family members possess broad mitogenic and cell survival activities and are involved in a variety of biological processes including embryonic development, cell growth, morphogenesis, tissue repair, tumor growth and invasion. This gene was identified by its similarity with mouse fgf3/int-2, a proto-oncogene activated in virally induced mammary tumors in the mouse. Frequent amplification of this gene has been found in human tumors, which may be important for neoplastic transformation and tumor progression. Studies of the similar genes in mouse and chicken suggested the role in inner ear formation. [provided by RefSeq, Jul 2008]
C11orf30	EMSY transcriptional repressor, BRCA2 interacting	Regulator which is able to repress transcription, possibly via its interaction with a multiprotein chromatin remodeling complex that modifies the chromatin (PubMed:14651845). Its interaction with BRCA2 suggests that it may play a central role in the DNA repair function of BRCA2 (PubMed:14651845). Mediates ligand-dependent transcriptional activation by nuclear hormone receptors (PubMed:19131338). Defects in EMSY may be a cause of sporadic breast cancer and higher-grade ovarian cancers. Overexpressed through amplification almost exclusively in sporadic breast cancer (13%) and higher-grade ovarian cancer (17%). Amplification is associated with worse survival, particularly in node-negative breast cancer, suggesting that it may be of prognostic value. Was named EMSY by PubMed:14651845 because the protein sequence contains the word 'SISTER', after the first author's sister, who is a breast cancer nurse. https://www.uniprot.org/uniprot/Q7Z589
EED	embryonic ectoderm development	This gene encodes a member of the Polycomb-group (PcG) family. PcG family members form multimeric protein complexes, which are involved in maintaining the transcriptional repressive state of genes over successive cell generations. This protein interacts with enhancer of zeste 2, the cytoplasmic tail of integrin beta7, immunodeficiency virus type 1 (HIV-1) MA protein, and histone deacetylase proteins. This protein mediates repression of gene activity through histone deacetylation, and may act as a specific regulator of integrin function. Two transcript variants encoding distinct isoforms have been identified for this gene. [provided by RefSeq, Jul 2008]
MRE11A	MRE11 homolog, double strand break repair nuclease	This gene encodes a nuclear protein involved in homologous recombination, telomere length maintenance, and DNA double-strand break repair. By itself, the protein has 3' to 5' exonuclease activity and endonuclease activity. The protein forms a complex with the RAD50 homolog; this complex is required for nonhomologous joining of DNA ends and possesses increased single-stranded DNA endonuclease and 3' to 5' exonuclease activities. In conjunction with a DNA ligase, this protein promotes the joining of noncomplementary ends in

		vitro using short homologies near the ends of the DNA fragments. This gene has a pseudogene on chromosome 3. Alternative splicing of this gene results in two transcript variants encoding different isoforms. [provided by RefSeq, Jul 2008]
ATM	ATM serine/threonine kinase	The protein encoded by this gene belongs to the PI3/PI4-kinase family. This protein is an important cell cycle checkpoint kinase that phosphorylates; thus, it functions as a regulator of a wide variety of downstream proteins, including tumor suppressor proteins p53 and BRCA1, checkpoint kinase CHK2, checkpoint proteins RAD17 and RAD9, and DNA repair protein NBS1. This protein and the closely related kinase ATR are thought to be master controllers of cell cycle checkpoint signaling pathways that are required for cell response to DNA damage and for genome stability. Mutations in this gene are associated with ataxia telangiectasia, an autosomal recessive disorder. [provided by RefSeq, Aug 2010]
SDHD	succinate dehydrogenase complex subunit D	This gene encodes a member of complex II of the respiratory chain, which is responsible for the oxidation of succinate. The encoded protein is one of two integral membrane proteins anchoring the complex to the matrix side of the mitochondrial inner membrane. Mutations in this gene are associated with the formation of tumors, including hereditary paraganglioma. Transmission of disease occurs almost exclusively through the paternal allele, suggesting that this locus may be maternally imprinted. There are pseudogenes for this gene on chromosomes 1, 2, 3, 7, and 18. Alternative splicing results in multiple transcript variants. [provided by RefSeq, Feb 2013]
CBL	Cbl proto-oncogene	This gene is a proto-oncogene that encodes a RING finger E3 ubiquitin ligase. The encoded protein is one of the enzymes required for targeting substrates for degradation by the proteasome. This protein mediates the transfer of ubiquitin from ubiquitin conjugating enzymes (E2) to specific substrates. This protein also contains an N-terminal phosphotyrosine binding domain that allows it to interact with numerous tyrosine-phosphorylated substrates and target them for proteasome degradation. As such it functions as a negative regulator of many signal transduction pathways. This gene has been found to be mutated or translocated in many cancers including acute myeloid leukaemia, and expansion of CGG repeats in the 5' UTR has been associated with Jacobsen syndrome. Mutations in this gene are also the cause of Noonan syndrome-like disorder. [provided by RefSeq, Jul 2016]
CHEK1	checkpoint kinase 1	The protein encoded by this gene belongs to the Ser/Thr protein kinase family. It is required for checkpoint mediated cell cycle arrest in response to DNA damage or the presence of unreplicated DNA. This protein acts to integrate signals from ATM and ATR, two cell cycle proteins involved in DNA damage responses, that also associate with chromatin in meiotic prophase I. Phosphorylation of CDC25A protein phosphatase by this protein is required for cells to delay cell cycle progression in response to double-strand DNA breaks. Several alternatively spliced transcript variants have been found for this gene. [provided by RefSeq, Oct 2011]
KDM5A	lysine demethylase 5A	This gene encodes a member of the Jumonji, AT-rich interactive domain 1 (JARID1) histone demethylase protein family. The encoded protein plays a role in gene regulation through the histone code by specifically demethylating lysine 4 of histone H3. The encoded protein interacts with many other proteins, including retinoblastoma protein, and is implicated in the transcriptional regulation of Hox genes and cytokines. This gene may play a role in tumor progression. [provided by RefSeq, Aug 2013]
RAD52	RAD52 homolog, DNA repair protein	The protein encoded by this gene shares similarity with Saccharomyces cerevisiae Rad52, a protein important for DNA double-strand break repair and homologous recombination. This gene product was shown to bind single-stranded DNA ends, and mediate the DNA-DNA interaction necessary for the annealing of complementary DNA strands. It was also found to interact with DNA recombination protein RAD51, which suggested its role in RAD51 related DNA recombination and repair. A pseudogene of this gene is present on chromosome 2. Alternative splicing results in multiple transcript variants. Additional alternatively spliced transcript variants of this gene have been described, but their full-length nature is not known. [provided by RefSeq, Jul 2014]
CCND2	cyclin D2	The protein encoded by this gene belongs to the highly conserved cyclin family, whose members are characterized by a dramatic periodicity in protein abundance through the cell cycle. Cyclins function as regulators of CDK kinases. Different cyclins exhibit distinct expression and degradation patterns which contribute to the temporal coordination of each mitotic event. This cyclin forms a complex with CDK4 or CDK6 and functions as a regulatory subunit of the complex, whose activity is required for cell cycle G1/S transition. This protein has been shown to interact with and be involved in the phosphorylation of tumor suppressor protein Rb. Knockout studies of the homologous gene in mouse suggest the essential roles of this gene in ovarian granulosa and germ cell proliferation. High level expression of this gene was observed in ovarian and testicular tumors. Mutations in this gene are associated with megalencephaly-polymicrogyria-polydactyly-hydrocephalus syndrome 3 (MPPH3). [provided by RefSeq, Sep 2014]
FGF23	fibroblast growth factor 23	This gene encodes a member of the fibroblast growth factor family of proteins, which possess broad mitogenic and cell survival activities and are involved in a variety of biological processes. The product of this gene regulates phosphate homeostasis and transport in the kidney. The full-length, functional protein may be deactivated via cleavage into N-terminal and C-terminal chains. Mutation of this cleavage site causes autosomal dominant hypophosphatemic rickets (ADHR). Mutations in this gene are also associated with hyperphosphatemic familial tumoral calcinosis (HFTC). [provided by RefSeq, Feb 2013]
FGF6	fibroblast growth factor 6	The protein encoded by this gene is a member of the fibroblast growth factor (FGF) family. FGF family members possess broad mitogenic and cell survival activities, and are involved in a variety of biological processes, including embryonic development, cell growth, morphogenesis, tissue repair, tumor growth and invasion. This gene displayed oncogenic transforming activity when transfected into mammalian cells. The mouse homolog of this gene exhibits a restricted expression profile predominantly in the myogenic lineage, which suggested a role in muscle regeneration or differentiation. [provided by RefSeq, Jul 2008]
ETV6	ETS variant transcription factor 6	This gene encodes an ETS family transcription factor. The product of this gene contains two functional domains: a N-terminal pointed (PNT) domain that is involved in protein-protein interactions with itself and other proteins, and a C-terminal DNA-binding domain. Gene knockout studies in mice suggest that it is required for hematopoiesis and maintenance of the developing vascular network. This gene is known to be involved in a large number of chromosomal rearrangements associated with leukemia and congenital fibrosarcoma. [provided by RefSeq, Sep 2008]

CDKN1B	cyclin dependent kinase inhibitor 1B	This gene encodes a cyclin-dependent kinase inhibitor, which shares a limited similarity with CDK inhibitor CDKN1A/p21. The encoded protein binds to and prevents the activation of cyclin E-CDK2 or cyclin D-CDK4 complexes, and thus controls the cell cycle progression at G1. The degradation of this protein, which is triggered by its CDK dependent phosphorylation and subsequent ubiquitination by SCF complexes, is required for the cellular transition from quiescence to the proliferative state. Mutations in this gene are associated with multiple endocrine neoplasia type IV (MEN4). [provided by RefSeq, Apr 2014]
PTPRO	protein tyrosine phosphatase receptor type O	This gene encodes a member of the R3 subtype family of receptor-type protein tyrosine phosphatases. These proteins are localized to the apical surface of polarized cells and may have tissue-specific functions through activation of Src family kinases. This gene contains two distinct promoters, and alternatively spliced transcript variants encoding multiple isoforms have been observed. The encoded proteins may have multiple isoform-specific and tissue-specific functions, including the regulation of osteoclast production and activity, inhibition of cell proliferation and facilitation of apoptosis. This gene is a candidate tumor suppressor, and decreased expression of this gene has been observed in several types of cancer. [provided by RefSeq, May 2011]
PIK3C2G	phosphatidylinositol-4-phosphate 3-kinase catalytic subunit type 2 gamma	The protein encoded by this gene belongs to the phosphoinositide 3-kinase (PI3K) family. PI3-kinases play roles in signaling pathways involved in cell proliferation, oncogenic transformation, cell survival, cell migration, and intracellular protein trafficking. This protein contains a lipid kinase catalytic domain as well as a C-terminal C2 domain, a characteristic of class II PI3-kinases. C2 domains act as calcium-dependent phospholipid binding motifs that mediate translocation of proteins to membranes and may also mediate protein-protein interactions. This gene may play a role in several diseases, including type II diabetes. Alternative splicing results in multiple transcript variants. [provided by RefSeq, Jan 2014]
KRAS	KRAS proto-oncogene, GTPase	This gene, a Kirsten ras oncogene homolog from the mammalian ras gene family, encodes a protein that is a member of the small GTPase superfamily. A single amino acid substitution is responsible for an activating mutation. The transforming protein that results is implicated in various malignancies, including lung adenocarcinoma, mucinous adenoma, ductal carcinoma of the pancreas and colorectal carcinoma. Alternative splicing leads to variants encoding two isoforms that differ in the C-terminal region. [provided by RefSeq, Jul 2008]
ACVR1B	activin A receptor type 1B	This gene encodes an activin A type IB receptor. Activins are dimeric growth and differentiation factors which belong to the transforming growth factor-beta (TGF-beta) superfamily of structurally related signaling proteins. Activins signal through a heteromeric complex of receptor serine kinases which include at least two type I and two type II receptors. This protein is a type I receptor which is essential for signaling. Mutations in this gene are associated with pituitary tumors. Alternate splicing results in multiple transcript variants.[provided by RefSeq, Jun 2010]
ERBB3	erb-b2 receptor tyrosine kinase 3	This gene encodes a member of the epidermal growth factor receptor (EGFR) family of receptor tyrosine kinases. This membrane-bound protein has a neuregulin binding domain but not an active kinase domain. It therefore can bind this ligand but not convey the signal into the cell through protein phosphorylation. However, it does form heterodimers with other EGF receptor family members which do have kinase activity. Heterodimerization leads to the activation of pathways which lead to cell proliferation or differentiation. Amplification of this gene and/or overexpression of its protein have been reported in numerous cancers, including prostate, bladder, and breast tumors. Alternate transcriptional splice variants encoding different isoforms have been characterized. One isoform lacks the intermembrane region and is secreted outside the cell. This form acts to modulate the activity of the membrane-bound form. Additional splice variants have also been reported, but they have not been thoroughly characterized. [provided by RefSeq, Jul 2008]
CDK4	cyclin dependent kinase 4	The protein encoded by this gene is a member of the Ser/Thr protein kinase family. This protein is highly similar to the gene products of S. cerevisiae cdc28 and S. pombe cdc2. It is a catalytic subunit of the protein kinase complex that is important for cell cycle G1 phase progression. The activity of this kinase is restricted to the G1-S phase, which is controlled by the regulatory subunits D-type cyclins and CDK inhibitor p16(INK4a). This kinase was shown to be responsible for the phosphorylation of retinoblastoma gene product (Rb). Mutations in this gene as well as in its related proteins including D-type cyclins, p16(INK4a) and Rb were all found to be associated with tumorigenesis of a variety of cancers. Multiple polyadenylation sites of this gene have been reported. [provided by RefSeq, Jul 2008]
MDM2	MDM2 proto-oncogene	This gene encodes a nuclear-localized E3 ubiquitin ligase. The encoded protein can promote tumor formation by targeting tumor suppressor proteins, such as p53, for proteasomal degradation. This gene is itself transcriptionally regulated by p53. Overexpression or amplification of this locus is detected in a variety of different cancers. There is a pseudogene for this gene on chromosome 2. Alternative splicing results in a multitude of transcript variants, many of which may be expressed only in tumor cells. [provided by RefSeq, Jun 2013]
BTG1	BTG anti-proliferation factor 1	This gene is a member of an anti-proliferative gene family that regulates cell growth and differentiation. Expression of this gene is highest in the G0/G1 phases of the cell cycle and downregulated when cells progressed through G1. The encoded protein interacts with several nuclear receptors, and functions as a coactivator of cell differentiation. This locus has been shown to be involved in a t(8;12)(q24;q22) chromosomal translocation in a case of B-cell chronic lymphocytic leukemia. [provided by RefSeq, Oct 2008]
PTPN11	protein tyrosine phosphatase non-receptor type 11	The protein encoded by this gene is a member of the protein tyrosine phosphatase (PTP) family. PTPs are known to be signaling molecules that regulate a variety of cellular processes including cell growth, differentiation, mitotic cycle, and oncogenic transformation. This PTP contains two tandem Src homology-2 domains, which function as phospho-tyrosine binding domains and mediate the interaction of this PTP with its substrates. This PTP is widely expressed in most tissues and plays a regulatory role in various cell signaling events that are important for a diversity of cell functions, such as mitogenic activation, metabolic control, transcription regulation, and cell migration. Mutations in this gene are a cause of Noonan syndrome as well as acute myeloid leukemia. [provided by RefSeq, Aug 2016]
TBX3	T-box transcription factor 3	This gene is a member of a phylogenetically conserved family of genes that share a common DNA-binding domain, the T-box. T-box genes encode transcription factors involved in the regulation of developmental processes. This protein is a transcriptional repressor and is thought to play a role in the anterior/posterior axis of the tetrapod forelimb. Mutations in this gene cause ulnar-mammary syndrome, affecting limb, apocrine gland, tooth, hair, and genital development. Alternative splicing of this gene results in three transcript variants

		encoding different isoforms; however, the full length nature of one variant has not been determined. [provided by RefSeq, Jul 2008]
HNF1A	HNF1 homeobox A	The protein encoded by this gene is a transcription factor required for the expression of several liver-specific genes. The encoded protein functions as a homodimer and binds to the inverted palindrome 5'-GTTAATNATTAAC-3'. Defects in this gene are a cause of maturity onset diabetes of the young type 3 (MODY3) and also can result in the appearance of hepatic adenomas. Alternative splicing results in multiple transcript variants encoding different isoforms. [provided by RefSeq, Apr 2015]
POLE	DNA polymerase epsilon, catalytic subunit	This gene encodes the catalytic subunit of DNA polymerase epsilon. The enzyme is involved in DNA repair and chromosomal DNA replication. Mutations in this gene have been associated with colorectal cancer 12 and facial dysmorphism, immunodeficiency, livedo, and short stature. [provided by RefSeq, Sep 2013]
CDK8	cyclin dependent kinase 8	This gene encodes a member of the cyclin-dependent protein kinase (CDK) family. CDK family members are known to be important regulators of cell cycle progression. This kinase and its regulatory subunit, cyclin C, are components of the Mediator transcriptional regulatory complex, involved in both transcriptional activation and repression by phosphorylation of the carboxy-terminal domain of the largest subunit of RNA polymerase II. This kinase regulates transcription by targeting the cyclin-dependent kinase 7 subunits of the general transcription initiation factor IIH, thus providing a link between the Mediator complex and the basal transcription machinery. Multiple pseudogenes of this gene have been identified. Alternative splicing results in multiple transcript variants. [provided by RefSeq, Oct 2016]
FLT3	fms related receptor tyrosine kinase 3	This gene encodes a class III receptor tyrosine kinase that regulates hematopoiesis. This receptor is activated by binding of the fms-related tyrosine kinase 3 ligand to the extracellular domain, which induces homodimer formation in the plasma membrane leading to autophosphorylation of the receptor. The activated receptor kinase subsequently phosphorylates and activates multiple cytoplasmic effector molecules in pathways involved in apoptosis, proliferation, and differentiation of hematopoietic cells in bone marrow. Mutations that result in the constitutive activation of this receptor result in acute myeloid leukemia and acute lymphoblastic leukemia. [provided by RefSeq, Jan 2015]
FLT1	fms related receptor tyrosine kinase 1	This gene encodes a member of the vascular endothelial growth factor receptor (VEGFR) family. VEGFR family members are receptor tyrosine kinases (RTKs) which contain an extracellular ligand-binding region with seven immunoglobulin (Ig)-like domains, a transmembrane segment, and a tyrosine kinase (TK) domain within the cytoplasmic domain. This protein binds to VEGFR-A, VEGFR-B and placental growth factor and plays an important role in angiogenesis and vasculogenesis. Expression of this receptor is found in vascular endothelial cells, placental trophoblast cells and peripheral blood monocytes. Multiple transcript variants encoding different isoforms have been found for this gene. Isoforms include a full-length transmembrane receptor isoform and shortened, soluble isoforms. The soluble isoforms are associated with the onset of pre-eclampsia.[provided by RefSeq, May 2009]
BRCA2	BRCA2 DNA repair associated	Inherited mutations in BRCA1 and this gene, BRCA2, confer increased lifetime risk of developing breast or ovarian cancer. Both BRCA1 and BRCA2 are involved in maintenance of genome stability, specifically the homologous recombination pathway for double-strand DNA repair. The largest exon in both genes is exon 11, which harbors the most important and frequent mutations in breast cancer patients. The BRCA2 gene was found on chromosome 13q12.3 in human. The BRCA2 protein contains several copies of a 70 aa motif called the BRC motif, and these motifs mediate binding to the RAD51 recombinase which functions in DNA repair. BRCA2 is considered a tumor suppressor gene, as tumors with BRCA2 mutations generally exhibit loss of heterozygosity (LOH) of the wild-type allele. [provided by RefSeq, May 2020]
RB1	RB transcriptional corepressor 1	The protein encoded by this gene is a negative regulator of the cell cycle and was the first tumor suppressor gene found. The encoded protein also stabilizes constitutive heterochromatin to maintain the overall chromatin structure. The active, hypophosphorylated form of the protein binds transcription factor E2F1. Defects in this gene are a cause of childhood cancer retinoblastoma (RB), bladder cancer, and osteogenic sarcoma. [provided by RefSeq, Jul 2008]
DIS3	DIS3 homolog, exosome endoribonuclease and 3'-5' exoribonuclease	Putative catalytic component of the RNA exosome complex which has 3'->5' exoribonuclease activity and participates in a multitude of cellular RNA processing and degradation events. In the nucleus, the RNA exosome complex is involved in proper maturation of stable RNA species such as rRNA, snRNA and snoRNA, in the elimination of RNA processing by-products and non-coding 'pervasive' transcripts, such as antisense RNA species and promoter-upstream transcripts (PROMPTs), and of mRNAs with processing defects, thereby limiting or excluding their export to the cytoplasm. The RNA exosome may be involved in Ig class switch recombination (CSR) and/or Ig variable region somatic hypermutation (SHM) by targeting AICDA deamination activity to transcribed dsDNA substrates. In the cytoplasm, the RNA exosome complex is involved in general mRNA turnover and specifically degrades inherently unstable mRNAs containing AU-rich elements (AREs) within their 3' untranslated regions, and in RNA surveillance pathways, preventing translation of aberrant mRNAs. It seems to be involved in degradation of histone mRNA. DIS3 has both 3'-5' exonuclease and endonuclease activities. https://www.uniprot.org/uniprot/Q9Y2L1
FGF14	fibroblast growth factor 14	The protein encoded by this gene is a member of the fibroblast growth factor (FGF) family. FGF family members possess broad mitogenic and cell survival activities, and are involved in a variety of biological processes, including embryonic development, cell growth, morphogenesis, tissue repair, tumor growth and invasion. A mutation in this gene is associated with autosomal dominant cerebral ataxia. Alternatively spliced transcript variants have been found for this gene. [provided by RefSeq, Jul 2008]
IRS2	insulin receptor substrate 2	This gene encodes the insulin receptor substrate 2, a cytoplasmic signaling molecule that mediates effects of insulin, insulin-like growth factor 1, and other cytokines by acting as a molecular adaptor between diverse receptor tyrosine kinases and downstream effectors. The product of this gene is phosphorylated by the insulin receptor tyrosine kinase upon receptor stimulation, as well as by an interleukin 4 receptor-associated kinase in response to IL4 treatment. [provided by RefSeq, Jul 2008]
CUL4A	cullin 4A	CUL4A is the ubiquitin ligase component of a multimeric complex involved in the degradation of DNA damage-response proteins (Liu et al., 2009 [PubMed 19481525]).[supplied by OMIM, Oct 2009]
PARP2	poly(ADP-ribose) polymerase 2	This gene encodes poly(ADP-ribosyl)transferase-like 2 protein, which contains a catalytic domain and is capable of catalyzing a poly(ADP-ribosyl)ation reaction. This protein has a catalytic domain which is homologous to that of poly (ADP-ribosyl) transferase, but lacks an N-terminal DNA binding domain which

		activates the C-terminal catalytic domain of poly (ADP-ribosyl) transferase. The basic residues within the N-terminal region of this protein may bear potential DNA-binding properties, and may be involved in the nuclear and/or nucleolar targeting of the protein. Two alternatively spliced transcript variants encoding distinct isoforms have been found. [provided by RefSeq, Jul 2008]
BCL2L2	BCL2 like 2	This gene encodes a member of the BCL-2 protein family. The proteins of this family form hetero- or homodimers and act as anti- and pro-apoptotic regulators. Expression of this gene in cells has been shown to contribute to reduced cell apoptosis under cytotoxic conditions. Studies of the related gene in mice indicated a role in the survival of NGF- and BDNF-dependent neurons. Mutation and knockout studies of the mouse gene demonstrated an essential role in adult spermatogenesis. Alternative splicing results in multiple transcript variants. Read-through transcription also exists between this gene and the neighboring downstream PABPN1 (poly(A) binding protein, nuclear 1) gene. [provided by RefSeq, Dec 2010]
NFKBIA	NFKB inhibitor alpha	This gene encodes a member of the NF-kappa-B inhibitor family, which contain multiple ankrin repeat domains. The encoded protein interacts with REL dimers to inhibit NF-kappa-B/REL complexes which are involved in inflammatory responses. The encoded protein moves between the cytoplasm and the nucleus via a nuclear localization signal and CRM1-mediated nuclear export. Mutations in this gene have been found in ectodermal dysplasia anhidrotic with T-cell immunodeficiency autosomal dominant disease. [provided by RefSeq, Aug 2011]
NKX21	NK2 homeobox 1	This gene encodes a protein initially identified as a thyroid-specific transcription factor. The encoded protein binds to the thyroglobulin promoter and regulates the expression of thyroid-specific genes but has also been shown to regulate the expression of genes involved in morphogenesis. Mutations and deletions in this gene are associated with benign hereditary chorea, choreoathetosis, congenital hypothyroidism, and neonatal respiratory distress, and may be associated with thyroid cancer. Multiple transcript variants encoding different isoforms have been found for this gene. This gene shares the symbol/alias 'TTF1' with another gene, transcription termination factor 1, which plays a role in ribosomal gene transcription. [provided by RefSeq, Feb 2014]
AKT1	AKT serine/threonine kinase 1	This gene encodes one of the three members of the human AKT serine-threonine protein kinase family which are often referred to as protein kinase B alpha, beta, and gamma. These highly similar AKT proteins all have an N-terminal pleckstrin homology domain, a serine/threonine-specific kinase domain and a C-terminal regulatory domain. These proteins are phosphorylated by phosphoinositide 3-kinase (PI3K). AKT/PI3K forms a key component of many signalling pathways that involve the binding of membrane-bound ligands such as receptor tyrosine kinases, G-protein coupled receptors, and integrin-linked kinase. These AKT proteins therefore regulate a wide variety of cellular functions including cell proliferation, survival, metabolism, and angiogenesis in both normal and malignant cells. AKT proteins are recruited to the cell membrane by phosphatidylinositol 3,4,5-trisphosphate (PIP3) after phosphorylation of phosphatidylinositol 4,5-bisphosphate (PIP2) by PI3K. Subsequent phosphorylation of both threonine residue 308 and serine residue 473 is required for full activation of the AKT1 protein encoded by this gene. Phosphorylation of additional residues also occurs, for example, in response to insulin growth factor-1 and epidermal growth factor. Protein phosphatases act as negative regulators of AKT proteins by dephosphorylating AKT or PIP3. The PI3K/AKT signalling pathway is crucial for tumor cell survival. Survival factors can suppress apoptosis in a transcription-independent manner by activating AKT1 which then phosphorylates and inactivates components of the apoptotic machinery. AKT proteins also participate in the mammalian target of rapamycin (mTOR) signalling pathway which controls the assembly of the eukaryotic translation initiation factor 4F (eIF4E) complex and this pathway, in addition to responding to extracellular signals from growth factors and cytokines, is disregulated in many cancers. Mutations in this gene are associated with multiple types of cancer and excessive tissue growth including Proteus syndrome and Cowden syndrome 6, and breast, colorectal, and ovarian cancers. Multiple alternatively spliced transcript variants have been found for this gene. [provided by RefSeq, Jul 2020]
RAD51	RAD51 recombinase	The protein encoded by this gene is a member of the RAD51 protein family. RAD51 family members are highly similar to bacterial RecA and Saccharomyces cerevisiae Rad51, and are known to be involved in the homologous recombination and repair of DNA. This protein can interact with the ssDNA-binding protein RPA and RAD52, and it is thought to play roles in homologous pairing and strand transfer of DNA. This protein is also found to interact with BRCA1 and BRCA2, which may be important for the cellular response to DNA damage. BRCA2 is shown to regulate both the intracellular localization and DNA-binding ability of this protein. Loss of these controls following BRCA2 inactivation may be a key event leading to genomic instability and tumorigenesis. Multiple transcript variants encoding different isoforms have been found for this gene. [provided by RefSeq, Aug 2009]
LTK	leukocyte receptor tyrosine kinase	The protein encoded by this gene is a member of the ros/insulin receptor family of tyrosine kinases. Tyrosine-specific phosphorylation of proteins is a key to the control of diverse pathways leading to cell growth and differentiation. Multiple transcript variants encoding different isoforms have been found for this gene. [provided by RefSeq, Oct 2008]
TYRO3	TYRO3 protein tyrosine kinase	The gene is part of a 3-member transmembrane receptor kinase receptor family with a processed pseudogene distal on chromosome 15. The encoded protein is activated by the products of the growth arrest-specific gene 6 and protein S genes and is involved in controlling cell survival and proliferation, spermatogenesis, immunoregulation and phagocytosis. The encoded protein has also been identified as a cell entry factor for Ebola and Marburg viruses. [provided by RefSeq, May 2010]
MAP2K1	mitogen-activated protein kinase kinase 1	The protein encoded by this gene is a member of the dual specificity protein kinase family, which acts as a mitogen-activated protein (MAP) kinase kinase. MAP kinases, also known as extracellular signal-regulated kinases (ERKs), act as an integration point for multiple biochemical signals. This protein kinase lies upstream of MAP kinases and stimulates the enzymatic activity of MAP kinases upon wide variety of extra- and intracellular signals. As an essential component of MAP kinase signal transduction pathway, this kinase is involved in many cellular processes such as proliferation, differentiation, transcription regulation and development. [provided by RefSeq, Jul 2008]
NTRK3	neurotrophic receptor tyrosine kinase 3	This gene encodes a member of the neurotrophic tyrosine receptor kinase (NTRK) family. This kinase is a membrane-bound receptor that, upon neurotrophin binding, phosphorylates itself and members of the MAPK pathway. Signalling through this kinase leads to cell differentiation and may play a role in the development of proprioceptive neurons that sense body position. Mutations in this gene have been associated with

		medulloblastomas, secretory breast carcinomas and other cancers. Several transcript variants encoding different isoforms have been found for this gene. [provided by RefSeq, Jul 2011]
IDH2	isocitrate dehydrogenase (NADP(+)) 2	Isocitrate dehydrogenases catalyze the oxidative decarboxylation of isocitrate to 2-oxoglutarate. These enzymes belong to two distinct subclasses, one of which utilizes NAD(+) as the electron acceptor and the other NADP(+). Five isocitrate dehydrogenases have been reported: three NAD(+)-dependent isocitrate dehydrogenases, which localize to the mitochondrial matrix, and two NADP(+)-dependent isocitrate dehydrogenases, one of which is mitochondrial and the other predominantly cytosolic. Each NADP(+)-dependent isozyme is a homodimer. The protein encoded by this gene is the NADP(+)-dependent isocitrate dehydrogenase found in the mitochondria. It plays a role in intermediary metabolism and energy production. This protein may tightly associate or interact with the pyruvate dehydrogenase complex. Alternative splicing results in multiple transcript variants. [provided by RefSeq, Feb 2014]
IGF1R	insulin like growth factor 1 receptor	This receptor binds insulin-like growth factor with a high affinity. It has tyrosine kinase activity. The insulin-like growth factor I receptor plays a critical role in transformation events. Cleavage of the precursor generates alpha and beta subunits. It is highly overexpressed in most malignant tissues where it functions as an anti-apoptotic agent by enhancing cell survival. Alternatively, spliced transcript variants encoding distinct isoforms have been found for this gene. [provided by RefSeq, May 2014]
AXIN1	axin 1	This gene encodes a cytoplasmic protein which contains a regulation of G-protein signaling (RGS) domain and a dishevelled and axin (DIX) domain. The encoded protein interacts with adenomatosis polyposis coli, catenin beta-1, glycogen synthase kinase 3 beta, protein phosphate 2, and itself. This protein functions as a negative regulator of the wingless-type MMTV integration site family, member 1 (WNT) signaling pathway and can induce apoptosis. The crystal structure of a portion of this protein, alone and in a complex with other proteins, has been resolved. Mutations in this gene have been associated with hepatocellular carcinoma, hepatoblastomas, ovarian endometriod adenocarcinomas, and medullablastomas. Alternative splicing results in multiple transcript variants. [provided by RefSeq, Jan 2016]
TSC2	TSC complex subunit 2	Mutations in this gene lead to tuberous sclerosis complex. Its gene product is believed to be a tumor suppressor and is able to stimulate specific GTPases. The protein associates with hamartin in a cytosolic complex, possibly acting as a chaperone for hamartin. Alternative splicing results in multiple transcript variants encoding different isoforms. [provided by RefSeq, Jul 2008]
CREBBP	CREB binding protein	This gene is ubiquitously expressed and is involved in the transcriptional coactivation of many different transcription factors. First isolated as a nuclear protein that binds to cAMP-response element binding protein (CREB), this gene is now known to play critical roles in embryonic development, growth control, and homeostasis by coupling chromatin remodeling to transcription factor recognition. The protein encoded by this gene has intrinsic histone acetyltransferase activity and also acts as a scaffold to stabilize additional protein interactions with the transcription complex. This protein acetylates both histone and non-histone proteins. This protein shares regions of very high sequence similarity with protein p300 in its bromodomain, cysteine-histidine-rich regions, and histone acetyltransferase domain. Mutations in this gene cause Rubinstein-Taybi syndrome (RTS). Chromosomal translocations involving this gene have been associated with acute myeloid leukemia. Alternative splicing results in multiple transcript variants encoding different isoforms. [provided by RefSeq, Feb 2009]
SOCS1	suppressor of cytokine signaling 1	This gene encodes a member of the STAT-induced STAT inhibitor (SSI), also known as suppressor of cytokine signaling (SOCS), family. SSI family members are cytokine-inducible negative regulators of cytokine signaling. The expression of this gene can be induced by a subset of cytokines, including IL2, IL3 erythropoietin (EPO), CSF2/GM-CSF, and interferon (IFN)-gamma. The protein encoded by this gene functions downstream of cytokine receptors, and takes part in a negative feedback loop to attenuate cytokine signaling. Knockout studies in mice suggested the role of this gene as a modulator of IFN-gamma action, which is required for normal postnatal growth and survival. [provided by RefSeq, Jul 2008]
ERCC4	ERCC excision repair 4, endonuclease catalytic subunit	The protein encoded by this gene forms a complex with ERCC1 and is involved in the 5' incision made during nucleotide excision repair. This complex is a structure specific DNA repair endonuclease that interacts with EME1. Defects in this gene are a cause of xeroderma pigmentosum complementation group F (XP-F), or xeroderma pigmentosum VI (XP6).[provided by RefSeq, Mar 2009]
PALB2	partner and localizer of BRCA2	This gene encodes a protein that may function in tumor suppression. This protein binds to and colocalizes with the breast cancer 2 early onset protein (BRCA2) in nuclear foci and likely permits the stable intranuclear localization and accumulation of BRCA2. [provided by RefSeq, Jul 2008]
CBFB	core-binding factor subunit beta	The protein encoded by this gene is the beta subunit of a heterodimeric core-binding transcription factor belonging to the PEBP2/CBF transcription factor family which master-regulates a host of genes specific to hematopoiesis (e.g., RUNX1) and osteogenesis (e.g., RUNX2). The beta subunit is a non-DNA binding regulatory subunit; it allosterically enhances DNA binding by alpha subunit as the complex binds to the core site of various enhancers and promoters, including murine leukemia virus, polyomavirus enhancer, T-cell receptor enhancers and GM-CSF promoters. Alternative splicing generates two mRNA variants, each encoding a distinct carboxyl terminus. In some cases, a pericentric inversion of chromosome 16 [inv(16)(p13q22)] produces a chimeric transcript consisting of the N terminus of core-binding factor beta in a fusion with the C-terminal portion of the smooth muscle myosin heavy chain 11. This chromosomal rearrangement is associated with acute myeloid leukemia of the M4Eo subtype. Two transcript variants encoding different isoforms have been found for this gene. [provided by RefSeq, Jul 2008]
CTCF	CCCTC-binding factor	This gene is a member of the BORIS + CTCF gene family and encodes a transcriptional regulator protein with 11 highly conserved zinc finger (ZF) domains. This nuclear protein is able to use different combinations of the ZF domains to bind different DNA target sequences and proteins. Depending upon the context of the site, the protein can bind a histone acetyltransferase (HAT)-containing complex and function as a transcriptional activator or bind a histone deacetylase (HDAC)-containing complex and function as a transcriptional repressor. If the protein is bound to a transcriptional insulator element, it can block communication between enhancers and upstream promoters, thereby regulating imprinted expression. Mutations in this gene have been associated with invasive breast cancers, prostate cancers, and Wilms' tumors. Alternatively, spliced transcript variants encoding different isoforms have been found for this gene. [provided by RefSeq, Jul 2010]

CDH1	cadherin 1	This gene encodes a classical cadherin of the cadherin superfamily. Alternative splicing results in multiple transcript variants, at least one of which encodes a preproprotein that is proteolytically processed to generate the mature glycoprotein. This calcium-dependent cell-cell adhesion protein is comprised of five extracellular cadherin repeats, a transmembrane region and a highly conserved cytoplasmic tail. Mutations in this gene are correlated with gastric, breast, colorectal, thyroid and ovarian cancer. Loss of function of this gene is thought to contribute to cancer progression by increasing proliferation, invasion, and/or metastasis. The ectodomain of this protein mediates bacterial adhesion to mammalian cells and the cytoplasmic domain is required for internalization. This gene is present in a gene cluster with other members of the cadherin family on chromosome 16. [provided by RefSeq, Nov 2015]
MAF	MAF bZIP transcription factor	The protein encoded by this gene is a DNA-binding, leucine zipper-containing transcription factor that acts as a homodimer or as a heterodimer. Depending on the binding site and binding partner, the encoded protein can be a transcriptional activator or repressor. This protein plays a role in the regulation of several cellular processes, including embryonic lens fiber cell development, increased T-cell susceptibility to apoptosis, and chondrocyte terminal differentiation. Defects in this gene are a cause of juvenile-onset pulverulent cataract as well as congenital cerulean cataract 4 (CCA4). Two transcript variants encoding different isoforms have been found for this gene. [provided by RefSeq, Jan 2010]
FANCA	FA complementation group A	The Fanconi anemia complementation group (FANC) currently includes FANCA, FANCB, FANCC, FANCD1 (also called BRCA2), FANCD2, FANCE, FANCF, FANCG, FANCI, FANCJ (also called BRIP1), FANCL, FANCM and FANCN (also called PALB2). The previously defined group FANCH is the same as FANCA. Fanconi anemia is a genetically heterogeneous recessive disorder characterized by cytogenetic instability, hypersensitivity to DNA crosslinking agents, increased chromosomal breakage, and defective DNA repair. The members of the Fanconi anemia complementation group do not share sequence similarity; they are related by their assembly into a common nuclear protein complex. This gene encodes the protein for complementation group A. Alternative splicing results in multiple transcript variants encoding different isoforms. Mutations in this gene are the most common cause of Fanconi anemia. [provided by RefSeq, Jul 2008]
TP53	tumor protein p53	This gene encodes a tumor suppressor protein containing transcriptional activation, DNA binding, and oligomerization domains. The encoded protein responds to diverse cellular stresses to regulate expression of target genes, thereby inducing cell cycle arrest, apoptosis, senescence, DNA repair, or changes in metabolism. Mutations in this gene are associated with a variety of human cancers, including hereditary cancers such as Li-Fraumeni syndrome. Alternative splicing of this gene and the use of alternate promoters result in multiple transcript variants and isoforms. Additional isoforms have also been shown to result from the use of alternate translation initiation codons from identical transcript variants (PMIDs: 12032546, 20937277). [provided by RefSeq, Dec 2016]
ALOX12B	arachidonate 12-lipoxygenase, 12R type	This gene encodes an enzyme involved in the conversion of arachidonic acid to 12R-hydroxyeicosatetraenoic acid. Mutations in this gene are associated with nonbullous congenital ichthyosiform erythroderma. [provided by RefSeq, Sep 2015]
AURKB	aurora kinase B	This gene encodes a member of the aurora kinase subfamily of serine/threonine kinases. The genes encoding the other two members of this subfamily are located on chromosomes 19 and 20. These kinases participate in the regulation of alignment and segregation of chromosomes during mitosis and meiosis through association with microtubules. A pseudogene of this gene is located on chromosome 8. Alternatively, spliced transcript variants have been found for this gene. [provided by RefSeq, Sep 2015]
MAP2K4	mitogen-activated protein kinase kinase 4	This gene encodes a member of the mitogen-activated protein kinase (MAPK) family. Members of this family act as an integration point for multiple biochemical signals and are involved in a wide variety of cellular processes such as proliferation, differentiation, transcription regulation, and development. They form a three-tiered signaling module composed of MAPKKKs, MAPKKs, and MAPKs. This protein is phosphorylated at serine and threonine residues by MAPKKKs and subsequently phosphorylates downstream MAPK targets at threonine and tyrosine residues. A similar protein in mouse has been reported to play a role in liver organogenesis. A pseudogene of this gene is located on the long arm of chromosome X. Alternative splicing results in multiple transcript variants. [provided by RefSeq, Jul 2013]
FLCN	folliculin	This gene is located within the Smith-Magenis syndrome region on chromosome 17. Mutations in this gene are associated with Birt-Hogg-Dube syndrome, which is characterized by fibrofolliculomas, renal tumors, lung cysts, and pneumothorax. Alternative splicing of this gene results in two transcript variants encoding different isoforms. [provided by RefSeq, Jul 2008]
NF1	neurofibromin 1	This gene product appears to function as a negative regulator of the ras signal transduction pathway. Mutations in this gene have been linked to neurofibromatosis type 1, juvenile myelomonocytic leukemia and Watson syndrome. The mRNA for this gene is subject to RNA editing (CGA>UGA->Arg1306Term) resulting in premature translation termination. Alternatively, spliced transcript variants encoding different isoforms have also been described for this gene. [provided by RefSeq, Jul 2008]
ERBB2	erb-b2 receptor tyrosine kinase 2	This gene encodes a member of the epidermal growth factor (EGF) receptor family of receptor tyrosine kinases. This protein has no ligand binding domain of its own and therefore cannot bind growth factors. However, it does bind tightly to other ligand-bound EGF receptor family members to form a heterodimer, stabilizing ligand binding and enhancing kinase-mediated activation of downstream signalling pathways, such as those involving mitogen-activated protein kinase and phosphatidylinositol-3 kinase. Allelic variations at amino acid positions 654 and 655 of isoform a (positions 624 and 625 of isoform b) have been reported, with the most common allele, Ile654/Ile655, shown here. Amplification and/or overexpression of this gene has been reported in numerous cancers, including breast and ovarian tumors. Alternative splicing results in several additional transcript variants, some encoding different isoforms and others that have not been fully characterized. [provided by RefSeq, Jul 2008]
RARA	retinoic acid receptor alpha	This gene represents a nuclear retinoic acid receptor. The encoded protein, retinoic acid receptor alpha, regulates transcription in a ligand-dependent manner. This gene has been implicated in regulation of development, differentiation, apoptosis, granulopoeisis, and transcription of clock genes. Translocations between this locus and several other loci have been associated with acute promyelocytic leukemia. Alternatively, spliced transcript variants have been found for this locus. [provided by RefSeq, Sep 2010]

STAT3	signal transducer and activator of transcription 3	The protein encoded by this gene is a member of the STAT protein family. In response to cytokines and growth factors, STAT family members are phosphorylated by the receptor associated kinases, and then form homo- or heterodimers that translocate to the cell nucleus where they act as transcription activators. This protein is activated through phosphorylation in response to various cytokines and growth factors including IFNs, EGF, IL5, IL6, HGF, LIF and BMP2. This protein mediates the expression of a variety of genes in response to cell stimuli, and thus plays a key role in many cellular processes such as cell growth and apoptosis. The small GTPase Rac1 has been shown to bind and regulate the activity of this protein. PIAS3 protein is a specific inhibitor of this protein. This gene also plays a role in regulating host response to viral and bacterial infections. Mutations in this gene are associated with infantile-onset multisystem autoimmune disease and hyper-immunoglobulin E syndrome. [provided by RefSeq, Aug 2020]
BRCA1	BRCA1 DNA repair associated	This gene encodes a 190 kD nuclear phosphoprotein that plays a role in maintaining genomic stability, and it also acts as a tumor suppressor. The BRCA1 gene contains 22 exons spanning about 110 kb of DNA. The encoded protein combines with other tumor suppressors, DNA damage sensors, and signal transducers to form a large multi-subunit protein complex known as the BRCA1-associated genome surveillance complex (BASC). This gene product associates with RNA polymerase II, and through the C-terminal domain, also interacts with histone deacetylase complexes. This protein thus plays a role in transcription, DNA repair of double-stranded breaks, and recombination. Mutations in this gene are responsible for approximately 40% of inherited breast cancers and more than 80% of inherited breast and ovarian cancers. Alternative splicing plays a role in modulating the subcellular localization and physiological function of this gene. Many alternatively spliced transcript variants, some of which are disease-associated mutations, have been described for this gene, but the full-length natures of only some of these variants has been described. A related pseudogene, which is also located on chromosome 17, has been identified. [provided by RefSeq, May 2020]
ETV4	ETS variant transcription factor 4	Transcriptional activator (PubMed:19307308, PubMed:31552090). May play a role in keratinocyte differentiation (PubMed:31552090). (Microbial infection) Binds to the enhancer of the adenovirus E1A gene and acts as a transcriptional activator; the core-binding sequence is 5'-[AC]GGA[AT]GT-3'. https://www.uniprot.org/uniprot/P43268
SPOP	speckle type BTB/POZ protein	This gene encodes a protein that may modulate the transcriptional repression activities of death-associated protein 6 (DAXX), which interacts with histone deacetylase, core histones, and other histone-associated proteins. In mouse, the encoded protein binds to the putative leucine zipper domain of macroH2A1.2, a variant H2A histone that is enriched on inactivated X chromosomes. The BTB/POZ domain of this protein has been shown in other proteins to mediate transcriptional repression and to interact with components of histone deacetylase co-repressor complexes. Alternative splicing of this gene results in multiple transcript variants encoding the same protein. [provided by RefSeq, Jul 2008]
RNF43	ring finger protein 43	The protein encoded by this gene is a RING-type E3 ubiquitin ligase and is predicted to contain a transmembrane domain, a protease-associated domain, an ectodomain, and a cytoplasmic RING domain. This protein is thought to negatively regulate Wnt signaling, and expression of this gene results in an increase in ubiquitination of frizzled receptors, an alteration in their subcellular distribution, resulting in reduced surface levels of these receptors. Mutations in this gene have been reported in multiple tumor cells, including colorectal and endometrial cancers. Alternative splicing results in multiple transcript variants encoding different isoforms. [provided by RefSeq, Mar 2015]
RAD51C	RAD51 paralog C	This gene is a member of the RAD51 family. RAD51 family members are highly similar to bacterial RecA and Saccharomyces cerevisiae Rad51 and are known to be involved in the homologous recombination and repair of DNA. This protein can interact with other RAD51 paralogs and is reported to be important for Holliday junction resolution. Mutations in this gene are associated with Fanconi anemia-like syndrome. This gene is one of four localized to a region of chromosome 17q23 where amplification occurs frequently in breast tumors. Overexpression of the four genes during amplification has been observed and suggests a possible role in tumor progression. Alternative splicing results in multiple transcript variants. [provided by RefSeq, Jul 2013]
BRIP1	BRCA1 interacting protein C-terminal helicase 1	The protein encoded by this gene is a member of the RecQ DEAH helicase family and interacts with the BRCT repeats of breast cancer, type 1 (BRCA1). The bound complex is important in the normal double-strand break repair function of breast cancer, type 1 (BRCA1). This gene may be a target of germline cancer-inducing mutations. [provided by RefSeq, Jul 2008]
CD79B	CD79b molecule	The B lymphocyte antigen receptor is a multimeric complex that includes the antigen-specific component, surface immunoglobulin (Ig). Surface Ig non-covalently associates with two other proteins, Ig-alpha and Ig-beta, which are necessary for expression and function of the B-cell antigen receptor. This gene encodes the Ig-beta protein of the B-cell antigen component. Alternatively spliced transcript variants encoding different isoforms have been described. [provided by RefSeq, Jul 2008]
GNA13	G protein subunit alpha 13	Guanine nucleotide-binding proteins (G proteins) are involved as modulators or transducers in various transmembrane signaling systems (PubMed:15240885, PubMed:16787920, PubMed:16705036, PubMed:27084452). Activates effector molecule RhoA by binding and activating RhoGEFs (ARHGEF1/p115RhoGEF, ARHGEF11/PDZ-RhoGEF and ARHGEF12/LARG) (PubMed:15240885, PubMed:12515866). GNA13-dependent Rho signaling subsequently regulates transcription factor AP-1 (activating protein-1) (By similarity). Promotes tumor cell invasion and metastasis by activating RhoA/ROCK signaling pathway (PubMed:16787920, PubMed:16705036, PubMed:27084452). Inhibits CDH1-mediated cell adhesion in process independent from Rho activation (PubMed:11976333). https://www.uniprot.org/uniprot/Q14344
PRKAR1A	protein kinase cAMP-dependent type I regulatory subunit alpha	cAMP is a signaling molecule important for a variety of cellular functions. cAMP exerts its effects by activating the cAMP-dependent protein kinase, which transduces the signal through phosphorylation of different target proteins. The inactive kinase holoenzyme is a tetramer composed of two regulatory and two catalytic subunits. cAMP causes the dissociation of the inactive holoenzyme into a dimer of regulatory subunits bound to four cAMP and two free monomeric catalytic subunits. Four different regulatory subunits and three catalytic subunits have been identified in humans. This gene encodes one of the regulatory subunits. This protein was found to be a tissue-specific extinguisher that down-regulates the expression of seven liver genes in hepatoma x fibroblast hybrids. Mutations in this gene cause Carney complex (CNC). This gene can fuse to the RET protooncogene by gene rearrangement and form the thyroid tumor-specific chimeric oncogene known as

		PTC2. A nonconventional nuclear localization sequence (NLS) has been found for this protein which suggests a role in DNA replication via the protein serving as a nuclear transport protein for the second subunit of the Replication Factor C (RFC40). Several alternatively spliced transcript variants encoding two different isoforms have been observed. [provided by RefSeq, Jan 2013]
SOX9	SRY-box transcription factor 9	The protein encoded by this gene recognizes the sequence CCTTGAG along with other members of the HMG-box class DNA-binding proteins. It acts during chondrocyte differentiation and, with steroidogenic factor 1, regulates transcription of the anti-Muellerian hormone (AMH) gene. Deficiencies lead to the skeletal malformation syndrome campomelic dysplasia, frequently with sex reversal. [provided by RefSeq, Jul 2008]
RPTOR	regulatory associated protein of MTOR complex 1	This gene encodes a component of a signaling pathway that regulates cell growth in response to nutrient and insulin levels. The encoded protein forms a stoichiometric complex with the mTOR kinase, and also associates with eukaryotic initiation factor 4E-binding protein-1 and ribosomal protein S6 kinase. The protein positively regulates the downstream effector ribosomal protein S6 kinase, and negatively regulates the mTOR kinase. Multiple transcript variants encoding different isoforms have been found for this gene. [provided by RefSeq, Sep 2009]
GATA6	GATA binding protein 6	This gene is a member of a small family of zinc finger transcription factors that play an important role in the regulation of cellular differentiation and organogenesis during vertebrate development. This gene is expressed during early embryogenesis and localizes to endo- and mesodermally derived cells during later embryogenesis and thereby plays an important role in gut, lung, and heart development. Mutations in this gene are associated with several congenital defects. [provided by RefSeq, Mar 2012]
SMAD2	SMAD family member 2	The protein encoded by this gene belongs to the SMAD, a family of proteins similar to the gene products of the Drosophila gene 'mothers against decapentaplegic' (Mad) and the C. elegans gene Sma. SMAD proteins are signal transducers and transcriptional modulators that mediate multiple signaling pathways. This protein mediates the signal of the transforming growth factor (TGF)-beta, and thus regulates multiple cellular processes, such as cell proliferation, apoptosis, and differentiation. This protein is recruited to the TGF-beta receptors through its interaction with the SMAD anchor for receptor activation (SARA) protein. In response to TGF-beta signal, this protein is phosphorylated by the TGF-beta receptors. The phosphorylation induces the dissociation of this protein with SARA and the association with the family member SMAD4. The association with SMAD4 is important for the translocation of this protein into the nucleus, where it binds to target promoters and forms a transcription repressor complex with other cofactors. This protein can also be phosphorylated by activin type 1 receptor kinase and mediates the signal from the activin. Alternatively, spliced transcript variants have been observed for this gene. [provided by RefSeq, May 2012]
SMAD4	SMAD family member 4	This gene encodes a member of the Smad family of signal transduction proteins. Smad proteins are phosphorylated and activated by transmembrane serine-threonine receptor kinases in response to transforming growth factor (TGF)-beta signaling. The product of this gene forms homomeric complexes and heteromeric complexes with other activated Smad proteins, which then accumulate in the nucleus and regulate the transcription of target genes. This protein binds to DNA and recognizes an 8-bp palindromic sequence (GTCTAGAC) called the Smad-binding element (SBE). The protein acts as a tumor suppressor and inhibits epithelial cell proliferation. It may also have an inhibitory effect on tumors by reducing angiogenesis and increasng blood vessel hyperpermeability. The encoded protein is a crucial component of the bone morphogenetic protein signaling pathway. The Smad proteins are subject to complex regulation by post-translational modifications. Mutations or deletions in this gene have been shown to result in pancreatic cancer, juvenile polyposis syndrome, and hereditary hemorrhagic telangiectasia syndrome. [provided by RefSeq, Aug 2017]
BCL2	BCL2 apoptosis regulator	This gene encodes an integral outer mitochondrial membrane protein that blocks the apoptotic death of some cells such as lymphocytes. Constitutive expression of BCL2, such as in the case of translocation of BCL2 to Ig heavy chain locus, is thought to be the cause of follicular lymphoma. Alternative splicing results in multiple transcript variants. [provided by RefSeq, Feb 2016]
DOT1L	DOT1 like histone lysine methyltransferase	The protein encoded by this gene is a histone methyltransferase that methylates lysine-79 of histone H3. It is inactive against free core histones but shows significant histone methyltransferase activity against nucleosomes. [provided by RefSeq, Aug 2011]
GNA11	G protein subunit alpha 11	The protein encoded by this gene belongs to the family of guanine nucleotide-binding proteins (G proteins), which function as modulators or transducers in various transmembrane signaling systems. G proteins are composed of 3 units: alpha, beta and gamma. This gene encodes one of the alpha subunits (subunit alpha-11). Mutations in this gene have been associated with hypocalciuric hypercalcemia type II (HHC2) and hypocalcemia dominant 2 (HYPOC2). Patients with HHC2 and HYPOC2 exhibit decreased or increased sensitivity, respectively, to changes in extracellular calcium concentrations. [provided by RefSeq, Dec 2013]
STK11	serine/threonine kinase 11	This gene, which encodes a member of the serine/threonine kinase family, regulates cell polarity and functions as a tumor suppressor. Mutations in this gene have been associated with Peutz-Jeghers syndrome, an autosomal dominant disorder characterized by the growth of polyps in the gastrointestinal tract, pigmented macules on the skin and mouth, and other neoplasms. Alternate transcriptional splice variants of this gene have been observed but have not been thoroughly characterized. [provided by RefSeq, Jul 2008]
MAP2K2	mitogen-activated protein kinase kinase 2	The protein encoded by this gene is a dual specificity protein kinase that belongs to the MAP kinase kinase family. This kinase is known to play a critical role in mitogen growth factor signal transduction. It phosphorylates and thus activates MAPK1/ERK2 and MAPK2/ERK3. The activation of this kinase itself is dependent on the Ser/Thr phosphorylation by MAP kinase kinase kinases. Mutations in this gene cause cardiofaciocutaneous syndrome (CFC syndrome), a disease characterized by heart defects, cognitive disability, and distinctive facial features similar to those found in Noonan syndrome. The inhibition or degradation of this kinase is also found to be involved in the pathogenesis of Yersinia and anthrax. A pseudogene, which is located on chromosome 7, has been identified for this gene. [provided by RefSeq, Jul 2008]
CD70	CD70 molecule	The protein encoded by this gene is a cytokine that belongs to the tumor necrosis factor (TNF) ligand family. This cytokine is a ligand for TNFRSF27/CD27. It is a surface antigen on activated, but not on resting, T and B lymphocytes. It induces proliferation of costimulated T cells, enhances the generation of cytolytic T cells, and contributes to T cell activation. This cytokine is also reported to play a role in regulating B-cell activation, cytotoxic function of natural killer cells, and immunoglobulin sythesis. [provided by RefSeq, Jul 2008]

KEAP1	kelch like ECH associated protein 1	This gene encodes a protein containing KELCH-1 like domains, as well as a BTB/POZ domain. Kelch-like ECH-associated protein 1 interacts with NF-E2-related factor 2 in a redox-sensitive manner and the dissociation of the proteins in the cytoplasm is followed by transportation of NF-E2-related factor 2 to the nucleus. This interaction results in the expression of the catalytic subunit of gamma-glutamyl cysteine synthetase. Two alternatively spliced transcript variants encoding the same isoform have been found for this gene. [provided by RefSeq, Jul 2008]
SMARCA4	SWI/SNF related, matrix associated, actin dependent regulator of chromatin, subfamily a, member 4	The protein encoded by this gene is a member of the SWI/SNF family of proteins and is similar to the brahma protein of Drosophila. Members of this family have helicase and ATPase activities and are thought to regulate transcription of certain genes by altering the chromatin structure around those genes. The encoded protein is part of the large ATP-dependent chromatin remodeling complex SNF/SWI, which is required for transcriptional activation of genes normally repressed by chromatin. In addition, this protein can bind BRCA1, as well as regulate the expression of the tumorigenic protein CD44. Mutations in this gene cause rhabdoid tumor predisposition syndrome type 2. Multiple transcript variants encoding different isoforms have been found for this gene. [provided by RefSeq, May 2012]
CALR	calreticulin	Calreticulin is a highly conserved chaperone protein which resides primarily in the endoplasmic reticulum, and is involved in a variety of cellular processes, among them, cell adhesion. Additionally, it functions in protein folding quality control and calcium homeostasis. Calreticulin is also found in the nucleus, suggesting that it may have a role in transcription regulation. Systemic lupus erythematosus is associated with increased autoantibody titers against calreticulin. Recurrent mutations in calreticulin have been linked to various neoplasms, including the myeloproliferative type. [provided by RefSeq, May 2020]
NOTCH3	notch receptor 3	This gene encodes the third discovered human homologue of the Drosophilia melanogaster type I membrane protein notch. In Drosophilia, notch interaction with its cell-bound ligands (delta, serrate) establishes an intercellular signalling pathway that plays a key role in neural development. Homologues of the notch-ligands have also been identified in human, but precise interactions between these ligands and the human notch homologues remains to be determined. Mutations in NOTCH3 have been identified as the underlying cause of cerebral autosomal dominant arteriopathy with subcortical infarcts and leukoencephalopathy (CADASIL). [provided by RefSeq, Jul 2008]
BRD4	bromodomain containing 4	The protein encoded by this gene is homologous to the murine protein MCAP, which associates with chromosomes during mitosis, and to the human RING3 protein, a serine/threonine kinase. Each of these proteins contains two bromodomains, a conserved sequence motif which may be involved in chromatin targeting. This gene has been implicated as the chromosome 19 target of translocation t(15;19)(q13;p13.1), which defines an upper respiratory tract carcinoma in young people. Two alternatively spliced transcript variants have been described. [provided by RefSeq, Jul 2008]
JAK3	Janus kinase 3	The protein encoded by this gene is a member of the Janus kinase (JAK) family of tyrosine kinases involved in cytokine receptor-mediated intracellular signal transduction. It is predominantly expressed in immune cells and transduces a signal in response to its activation via tyrosine phosphorylation by interleukin receptors. Mutations in this gene are associated with autosomal SCID (severe combined immunodeficiency disease). [provided by RefSeq, Jul 2008]
MEF2B	myocyte enhancer factor 2B	The product of this gene is a member of the MADS/MEF2 family of DNA binding proteins. The protein is thought to regulate gene expression, including expression of the smooth muscle myosin heavy chain gene. This region undergoes considerable alternative splicing, with transcripts supporting two non-overlapping loci (GeneID 729991 and 100271849) as well as numerous read-through transcripts that span both loci (annotated as GeneID 4207). Several isoforms of this protein are expressed from either this locus or from some of the read-through transcripts annotated on GeneID 4207. [provided by RefSeq, Jan 2014]
CCNE1	cyclin E1	The protein encoded by this gene belongs to the highly conserved cyclin family, whose members are characterized by a dramatic periodicity in protein abundance through the cell cycle. Cyclins function as regulators of CDK kinases. Different cyclins exhibit distinct expression and degradation patterns which contribute to the temporal coordination of each mitotic event. This cyclin forms a complex with and functions as a regulatory subunit of CDK2, whose activity is required for cell cycle G1/S transition. This protein accumulates at the G1-S phase boundary and is degraded as cells progress through S phase. Overexpression of this gene has been observed in many tumors, which results in chromosome instability, and thus may contribute to tumorigenesis. This protein was found to associate with, and be involved in, the phosphorylation of NPAT protein (nuclear protein mapped to the ATM locus), which participates in cell-cycle regulated histone gene expression and plays a critical role in promoting cell-cycle progression in the absence of pRB. [provided by RefSeq, Apr 2016]
CEBPA	CCAAT enhancer binding protein alpha	This intronless gene encodes a transcription factor that contains a basic leucine zipper (bZIP) domain and recognizes the CCAAT motif in the promoters of target genes. The encoded protein functions in homodimers and also heterodimers with CCAAT/enhancer-binding proteins beta and gamma. Activity of this protein can modulate the expression of genes involved in cell cycle regulation as well as in body weight homeostasis. Mutation of this gene is associated with acute myeloid leukemia. The use of alternative in-frame non-AUG (GUG) and AUG start codons results in protein isoforms with different lengths. Differential translation initiation is mediated by an out-of-frame, upstream open reading frame which is located between the GUG and the first AUG start codons. [provided by RefSeq, Dec 2013]
CD22	CD22 molecule	Mediates B-cell B-cell interactions. May be involved in the localization of B-cells in lymphoid tissues. Binds sialylated glycoproteins; one of which is CD45. Preferentially binds to alpha-2,6-linked sialic acid. The sialic acid recognition site can be masked by cis interactions with sialic acids on the same cell surface. Upon ligand induced tyrosine phosphorylation in the immune response seems to be involved in regulation of B-cell antigen receptor signaling. Plays a role in positive regulation through interaction with Src family tyrosine kinases and may also act as an inhibitory receptor by recruiting cytoplasmic phosphatases via their SH2 domains that block signal transduction through dephosphorylation of signaling molecules. https://www.uniprot.org/uniprot/P20273
AKT2	AKT serine/threonine kinase 2	This gene is a putative oncogene encoding a protein belonging to a subfamily of serine/threonine kinases containing SH2-like (Src homology 2-like) domains, which is involved in signaling pathways. The gene serves as an oncogene in the tumorigenesis of cancer cells For example, its overexpression contributes to the malignant phenotype of a subset of human ductal pancreatic cancers. The encoded protein is a general protein

		kinase capable of phophorylating several known proteins and has also been implicated in insulin signaling. [provided by RefSeq, Nov 2019]
AXL	AXL receptor tyrosine kinase	The protein encoded by this gene is a member of the Tyro3-Axl-Mer (TAM) receptor tyrosine kinase subfamily. The encoded protein possesses an extracellular domain which is composed of two immunoglobulin-like motifs at the N-terminal, followed by two fibronectin type-III motifs. It transduces signals from the extracellular matrix into the cytoplasm by binding to the vitamin K-dependent protein growth arrest-specific 6 (Gas6). This gene may be involved in several cellular functions including growth, migration, aggregation and anti-inflammation in multiple cell types. Alternative splicing results in multiple transcript variants of this gene. [provided by RefSeq, Jul 2013]
CD79A	CD79a molecule	The B lymphocyte antigen receptor is a multimeric complex that includes the antigen-specific component, surface immunoglobulin (Ig). Surface Ig non-covalently associates with two other proteins, Ig-alpha and Ig-beta, which are necessary for expression and function of the B-cell antigen receptor. This gene encodes the Ig-alpha protein of the B-cell antigen component. Alternatively, spliced transcript variants encoding different isoforms have been described. [provided by RefSeq, Jul 2008]
CIC	capicua transcriptional repressor	The protein encoded by this gene is an ortholog of the Drosophila melanogaster capicua gene and is a member of the high mobility group (HMG)-box superfamily of transcriptional repressors. This protein contains a conserved HMG domain that is involved in DNA binding and nuclear localization, and a conserved C-terminus. Studies suggest that the N-terminal region of this protein interacts with Atxn1 (GeneID:6310), to form a transcription repressor complex, and in vitro studies suggest that polyglutamine-expansion of ATXN1 may alter the repressor activity of this complex. Mutations in this gene have been associated with olidogdendrogliomas (PMID:21817013). In addition, translocation events resulting in gene fusions of this gene with both DUX4 (GeneID:100288687) and FOXO4 (GeneID:4303) have been associated with round cell sarcomas. There are multiple pseudogenes of this gene found on chromosomes 1, 4, 6, 7, 16, 20, and the Y chromosome. Alternative splicing results in multiple transcript variants encoding different isoforms. [provided by RefSeq, Feb 2015]
POLD1	DNA polymerase delta 1, catalytic subunit	This gene encodes the 125-kDa catalytic subunit of DNA polymerase delta. DNA polymerase delta possesses both polymerase and 3' to 5' exonuclease activity and plays a critical role in DNA replication and repair. Alternatively, spliced transcript variants have been observed for this gene, and a pseudogene of this gene is located on the long arm of chromosome 6. [provided by RefSeq, Mar 2012]
BCL2L1	BCL2 like 1	The protein encoded by this gene belongs to the BCL-2 protein family. BCL-2 family members form hetero- or homodimers and act as anti- or pro-apoptotic regulators that are involved in a wide variety of cellular activities. The proteins encoded by this gene are located at the outer mitochondrial membrane and have been shown to regulate outer mitochondrial membrane channel (VDAC) opening. VDAC regulates mitochondrial membrane potential, and thus controls the production of reactive oxygen species and release of cytochrome C by mitochondria, both of which are the potent inducers of cell apoptosis. Alternative splicing results in multiple transcript variants encoding two different isoforms. The longer isoform acts as an apoptotic inhibitor and the shorter isoform acts as an apoptotic activator. [provided by RefSeq, Dec 2015]
ASXL1	ASXL transcriptional regulator 1	This gene is similar to the Drosophila additional sex combs gene, which encodes a chromatin-binding protein required for normal determination of segment identity in the developing embryo. The protein is a member of the Polycomb group of proteins, which are necessary for the maintenance of stable repression of homeotic and other loci. The protein is thought to disrupt chromatin in localized areas, enhancing transcription of certain genes while repressing the transcription of other genes. The protein encoded by this gene functions as a ligand-dependent co-activator for retinoic acid receptor in cooperation with nuclear receptor coactivator 1. Mutations in this gene are associated with myelodysplastic syndromes and chronic myelomonocytic leukemia. Alternative splicing results in multiple transcript variants. [provided by RefSeq, Sep 2009]
SRC	SRC proto-oncogene, non-receptor tyrosine kinase	This gene is highly like the v-src gene of Rous sarcoma virus. This proto-oncogene may play a role in the regulation of embryonic development and cell growth. The protein encoded by this gene is a tyrosine-protein kinase whose activity can be inhibited by phosphorylation by c-SRC kinase. Mutations in this gene could be involved in the malignant progression of colon cancer. Two transcript variants encoding the same protein have been found for this gene. [provided by RefSeq, Jul 2008]
SDC4	syndecan 4	The protein encoded by this gene is a transmembrane (type I) heparan sulfate proteoglycan that functions as a receptor in intracellular signaling. The encoded protein is found as a homodimer and is a member of the syndecan proteoglycan family. This gene is found on chromosome 20, while a pseudogene has been found on chromosome 22. [provided by RefSeq, Jul 2008]
ZNF217	zinc finger protein 217	Binds to the promoters of target genes and functions as repressor. Promotes cell proliferation and antagonizes cell death. Promotes phosphorylation of AKT1 at 'Ser-473'. https://www.uniprot.org/uniprot/O75362
AURKA	aurora kinase A	The protein encoded by this gene is a cell cycle-regulated kinase that appears to be involved in microtubule formation and/or stabilization at the spindle pole during chromosome segregation. The encoded protein is found at the centrosome in interphase cells and at the spindle poles in mitosis. This gene may play a role in tumor development and progression. A processed pseudogene of this gene has been found on chromosome 1, and an unprocessed pseudogene has been found on chromosome 10. Multiple transcript variants encoding the same protein have been found for this gene. [provided by RefSeq, Jul 2008]
GNAS	GNAS complex locus	This locus has a highly complex imprinted expression pattern. It gives rise to maternally, paternally, and biallelically expressed transcripts that are derived from four alternative promoters and 5' exons. Some transcripts contain a differentially methylated region (DMR) at their 5' exons, and this DMR is commonly found in imprinted genes and correlates with transcript expression. An antisense transcript is produced from an overlapping locus on the opposite strand. One of the transcripts produced from this locus, and the antisense transcript, are paternally expressed noncoding RNAs, and may regulate imprinting in this region. In addition, one of the transcripts contains a second overlapping ORF, which encodes a structurally unrelated protein - Alex. Alternative splicing of downstream exons is also observed, which results in different forms of the stimulatory G-protein alpha subunit, a key element of the classical signal transduction pathway linking receptor-ligand interactions with the activation of adenylyl cyclase and a variety of cellular reponses. Multiple transcript variants encoding different isoforms have been found for this gene. Mutations in this gene result in pseudohypoparathyroidism type 1a, pseudohypoparathyroidism type 1b, Albright hereditary osteodystrophy,

		pseudopseudohypoparathyroidism, McCune-Albright syndrome, progressive osseus heteroplasia, polyostotic fibrous dysplasia of bone, and some pituitary tumors. [provided by RefSeq, Aug 2012]
ARFRP1	ADP ribosylation factor related protein 1	The protein encoded by this gene is a membrane-associated GTP-ase which localizes to the plasma membrane and is related to the ADP-ribosylation factor (ARF) and ARF-like (ARL) proteins. This gene plays a role in membrane trafficking between the trans-Golgi network and endosomes. Alternatively, spliced transcript variants encoding different isoforms have been identified. [provided by RefSeq, May 2012]
ERG	ETS transcription factor ERG	This gene encodes a member of the erythroblast transformation specific (ETS) family of transcriptions factors. All members of this family are key regulators of embryonic development, cell proliferation, differentiation, angiogenesis, inflammation, and apoptosis. The protein encoded by this gene is mainly expressed in the nucleus. It contains an ETS DNA-binding domain and a PNT (pointed) domain which is implicated in the self-association of chimeric oncoproteins. This protein is required for platelet adhesion to the subendothelium, inducing vascular cell remodeling. It also regulates hematopoesis, and the differentiation and maturation of megakaryocytic cells. This gene is involved in chromosomal translocations, resulting in different fusion gene products, such as TMPSSR2-ERG and NDRG1-ERG in prostate cancer, EWS-ERG in Ewing's sarcoma and FUS-ERG in acute myeloid leukemia. More than two dozen of transcript variants generated from combinatorial usage of three alternative promoters and multiple alternative splicing events have been reported, but the full-length nature of many of these variants has not been determined. [provided by RefSeq, Apr 2014]
TMPRSS2	transmembrane serine protease 2	This gene encodes a protein that belongs to the serine protease family. The encoded protein contains a type II transmembrane domain, a receptor class A domain, a scavenger receptor cysteine-rich domain and a protease domain. Serine proteases are known to be involved in many physiological and pathological processes. This gene was demonstrated to be up-regulated by androgenic hormones in prostate cancer cells and down-regulated in androgen-independent prostate cancer tissue. The protease domain of this protein is thought to be cleaved and secreted into cell media after autocleavage. This protein also facilitates entry of viruses into host cells by proteolytically cleaving and activating viral envelope glycoproteins. Viruses found to use this protein for cell entry include Influenza virus and the human coronaviruses HCoV-229E, MERS-CoV, SARS-CoV and SARS-CoV-2 (COVID-19 virus). Alternatively, spliced transcript variants encoding different isoforms have been found for this gene. [provided by RefSeq, Apr 2020]
U2AF1	U2 small nuclear RNA auxiliary factor 1	This gene belongs to the splicing factor SR family of genes. U2 auxiliary factor, comprising a large and a small subunit, is a non-snRNP protein required for the binding of U2 snRNP to the pre-mRNA branch site. This gene encodes the small subunit which plays a critical role in both constitutive and enhancer-dependent RNA splicing by directly mediating interactions between the large subunit and proteins bound to the enhancers. Alternatively spliced transcript variants encoding different isoforms have been identified. [provided by RefSeq, Jul 2008]
CRKL	CRK like proto-oncogene, adaptor protein	This gene encodes a protein kinase containing SH2 and SH3 (src homology) domains which has been shown to activate the RAS and JUN kinase signaling pathways and transform fibroblasts in a RAS-dependent fashion. It is a substrate of the BCR-ABL tyrosine kinase, plays a role in fibroblast transformation by BCR-ABL, and may be oncogenic.[provided by RefSeq, Jan 2009]
MAPK1	mitogen-activated protein kinase 1	This gene encodes a member of the MAP kinase family. MAP kinases, also known as extracellular signal-regulated kinases (ERKs), act as an integration point for multiple biochemical signals, and are involved in a wide variety of cellular processes such as proliferation, differentiation, transcription regulation and development. The activation of this kinase requires its phosphorylation by upstream kinases. Upon activation, this kinase translocates to the nucleus of the stimulated cells, where it phosphorylates nuclear targets. One study also suggests that this protein acts as a transcriptional repressor independent of its kinase activity. The encoded protein has been identified as a moonlighting protein based on its ability to perform mechanistically distinct functions. Two alternatively spliced transcript variants encoding the same protein, but differing in the UTRs, have been reported for this gene. [provided by RefSeq, Jan 2014]
BCR	BCR activator of RhoGEF and GTPase	A reciprocal translocation between chromosomes 22 and 9 produces the Philadelphia chromosome, which is often found in patients with chronic myelogenous leukemia. The chromosome 22 breakpoint for this translocation is located within the BCR gene. The translocation produces a fusion protein which is encoded by sequence from both BCR and ABL, the gene at the chromosome 9 breakpoint. Although the BCR-ABL fusion protein has been extensively studied, the function of the normal BCR gene product is not clear. The unregulated tyrosine kinase activity of BCR-ABL1 contributes to the immortality of leukaemic cells. The BCR protein has serine/threonine kinase activity and is a GTPase-activating protein for p21rac and other kinases. Two transcript variants encoding different isoforms have been found for this gene.[provided by RefSeq, Jan 2020]
SMARCB1	SWI/SNF related, matrix associated, actin dependent regulator of chromatin, subfamily b, member 1	The protein encoded by this gene is part of a complex that relieves repressive chromatin structures, allowing the transcriptional machinery to access its targets more effectively. The encoded nuclear protein may also bind to and enhance the DNA joining activity of HIV-1 integrase. This gene has been found to be a tumor suppressor, and mutations in it have been associated with malignant rhabdoid tumors. Alternatively, spliced transcript variants have been found for this gene. [provided by RefSeq, Dec 2015]
CHEK2	checkpoint kinase 2	In response to DNA damage and replication blocks, cell cycle progression is halted through the control of critical cell cycle regulators. The protein encoded by this gene is a cell cycle checkpoint regulator and putative tumor suppressor. It contains a forkhead-associated protein interaction domain essential for activation in response to DNA damage and is rapidly phosphorylated in response to replication blocks and DNA damage. When activated, the encoded protein is known to inhibit CDC25C phosphatase, preventing entry into mitosis, and has been shown to stabilize the tumor suppressor protein p53, leading to cell cycle arrest in G1. In addition, this protein interacts with and phosphorylates BRCA1, allowing BRCA1 to restore survival after DNA damage. Mutations in this gene have been linked with Li-Fraumeni syndrome, a highly penetrant familial cancer phenotype usually associated with inherited mutations in TP53. Also, mutations in this gene are thought to confer a predisposition to sarcomas, breast cancer, and brain tumors. This nuclear protein is a member of the CDS1 subfamily of serine/threonine protein kinases. Several transcript variants encoding different isoforms have been found for this gene. [provided by RefSeq, Apr 2012]

EWSR1	EWS RNA binding protein 1	This gene encodes a multifunctional protein that is involved in various cellular processes, including gene expression, cell signaling, and RNA processing and transport. The protein includes an N-terminal transcriptional activation domain and a C-terminal RNA-binding domain. Chromosomal translocations between this gene and various genes encoding transcription factors result in the production of chimeric proteins that are involved in tumorigenesis. These chimeric proteins usually consist of the N-terminal transcriptional activation domain of this protein fused to the C-terminal DNA-binding domain of the transcription factor protein. Mutations in this gene, specifically a t(11;22)(q24;q12) translocation, are known to cause Ewing sarcoma as well as neuroectodermal and various other tumors. Alternative splicing of this gene results in multiple transcript variants. Related pseudogenes have been identified on chromosomes 1 and 14. [provided by RefSeq, Jul 2009]
NF2	neurofibromin 2	This gene encodes a protein that is similar to some members of the ERM (ezrin, radixin, moesin) family of proteins that are thought to link cytoskeletal components with proteins in the cell membrane. This gene product has been shown to interact with cell-surface proteins, proteins involved in cytoskeletal dynamics and proteins involved in regulating ion transport. This gene is expressed at high levels during embryonic development; in adults, significant expression is found in Schwann cells, meningeal cells, lens and nerve. Mutations in this gene are associated with neurofibromatosis type II which is characterized by nervous system and skin tumors and ocular abnormalities. Two predominant isoforms and a number of minor isoforms are produced by alternatively spliced transcripts. [provided by RefSeq, Jul 2008]
EP300	E1A binding protein p300	This gene encodes the adenovirus E1A-associated cellular p300 transcriptional co-activator protein. It functions as histone acetyltransferase that regulates transcription via chromatin remodeling and is important in the processes of cell proliferation and differentiation. It mediates cAMP-gene regulation by binding specifically to phosphorylated CREB protein. This gene has also been identified as a co-activator of HIF1A (hypoxia-inducible factor 1 alpha), and thus plays a role in the stimulation of hypoxia-induced genes such as VEGF. Defects in this gene are a cause of Rubinstein-Taybi syndrome and may also play a role in epithelial cancer. [provided by RefSeq, Jul 2008]

Supplementary Table S2. Patient Characteristics.

	CRC STAGE 2 *n=212*	CRC STAGE 3 *n=166*	P-value
**DFS in Months (mean)**	*29.08*	*23.56*	0.038
**Patient Status (%)**			<0.001
Recurrent	61 (28.8)	51 (30.5)	
Non-Recurrent	151 (71.2)	115 (69.5)	
**NTE (%)**			0.428
Absent/Treatment	139 (65.6)	103 (62.0)	
Present/Treatment	28 (13.2)	30 (18.1)	
Treatment Not Indicated	45 (21.2)	33 (19.9)	
**AJCC T Stage (%)**			0.001
T1	1 (0.5)	2 (1.2)	
T2	1 (0.5)	12 (7.2)	
T3	196 (92.5)	133 (80.1)	
T4	14 (6.6)	19 (11.4)	
**AJCC N Stage (%)**			<0.001
N0	210 (99.1)	0 (0.0)	
N1	0 (0.0)	106 (63.5)	
N2	0 (0.0)	60 (35.9)	
NX	2 (0.9)	1 (0.6)	
**AJCC M Stage (%)**			0.016
M0	198 (94.3)	139 (85.8)	
M1	0 (0.0)	1 (0.6)	
MX	12 (5.7)	22 (13.6)	
**Age at Diagnosis (%)**			0.011
0 to 49	16 (7.5)	27 (16.3)	
50 to 64	52 (24.5)	47 (28.3)	
Over 65	144 (67.9)	92 (55.4)	
**Sex at Birth (%)**			0.343
Female	97 (45.8)	86 (51.2)	
Male	115 (54.2)	81 (48.8)	
**Microsatellite Status (%)**			0.002
MSI-High	46 (21.7)	15 (9.0)	
MSI-Low	34 (16.0)	22 (13.3)	
MSS (stable)	132 (62.3)	129 (77.7)	
**Consensus Molecular Subtype (%)**			0.01
CMS1	44 (20.8)	16 (9.6)	
CMS2	79 (37.3)	72 (43.4)	
CMS3	32 (15.1)	19 (11.4)	
CMS4	57 (26.9)	59 (35.5)	
**Primary Site of Tumor (%)**			0.083
Ascending Colon	45 (21.1)	23 (13.8)	
Cecum	29 (13.6)	34 (20.4)	
Descending Colon	6 (2.8)	7 (4.2)	
Hepatic Flexure	15 (7.0)	8 (4.8)	
Rectosigmoid Junction	12 (5.6)	19 (11.4)	
Rectum	38 (17.8)	30 (18.0)	
Sigmoid Colon	51 (23.9)	38 (22.8)	
Splenic Flexure	4 (1.9)	0 (0.0)	
Transverse Colon	13 (6.1)	8 (4.8)	

**Vascular Invasion (%)**			0.093
Vascular Invasion	42 (19.8)	46 (27.7)	
None	170 (80.2)	121 (72.3)	
**Lymphovascular Invasion (%)**			<0.001
Lymphovascular Invasion	49 (23.1)	104 (62.7)	
None	162 (76.9)	63 (37.3)	
**Perineural Invasion (%)**			0.682
Perineural Invasion	95 (44.8)	70 (42.2)	
None	117 (55.2)	97 (57.8)	
**KRAS Abnormality (%)**			0.986
Abnormal	9 (4.2)	8 (4.8)	
Normal	203 (95.8)	159 (95.2)	
**Body Mass Index (%)**			0.173
≥18.5 BMI <25.0	73 (34.4)	43 (25.9)	
≥25.0 BMI <30.0	80 (37.7)	75 (45.2)	
BMI Over 30	59 (27.8)	48 (28.9)	
**MGS02PS04 Mutation-Negative (%)**			0.079
CLASS02=NORMAL	28 (13.2)	34 (20.5)	
ABSENT	184 (86.8)	133 (79.5)	
**MGS02PS03 Mutation-Negative (%)**			0.062
CLASS02=NORMAL	24 (11.3)	31 (18.7)	
ABSENT	188 (88.7)	136 (81.3)	
**MGS02PS04 Mutation-Positive (%)**			0.346
CLASS01=MUTATION	56 (26.4)	36 (21.7)	
ABSENT	156 (73.6)	131 (78.3)	
**MGS02PS05 Mutation-Positive (%)**			0.644
CLASS01=MUTATION	22 (10.4)	14 (8.4)	
ABSENT	190 (89.6)	153 (91.6)	
**MG03PS18 Mutation-Negative (%)**			0.573
CLASS02=NORMAL	177 (83.5)	134 (80.7)	
ABSENT	35 (16.5)	33 (19.3)	
**MG03PS19 Mutation-Negative (%)**			0.895
CLASS02=NORMAL	174 (82.1)	138 (83.1)	
ABSENT	38 (17.9)	29 (16.9)	
**MG03PS19 Mutation-Positive (%)**			0.992
CLASS01=MUTATION	5 (2.4)	3 (1.8)	
ABSENT	207 (97.6)	134 (98.2)	
**MG03PS20 Mutation-Positive (%)**			0.649
CLASS01=MUTATION	8 (3.8)	4 (2.4)	
ABSENT	204 (96.2)	133 (97.6)	
**MG23PS18 Mutation-Negative (%)**			0.004
CLASS02=NORMAL	50 (23.6)	19 (11.4)	
ABSENT	162 (76.4)	148 (88.6)	
**MG23PS19 Mutation-Negative (%)**			0.009
CLASS02=NORMAL	44 (20.8)	17 (10.2)	
ABSENT	168 (79.2)	150 (79.8)	
**MG23PS18 Mutation-Positive (%)**			0.044
CLASS01=MUTATION	61 (28.8)	65 (39.2)	
ABSENT	151 (71.2)	102 (60.8)	
**MG23PS19 Mutation-Positive (%)**			0.142
CLASS01=MUTATION	13 (6.1)	18 (10.8)	
ABSENT	199 (95.9)	149 (89.2)	
**MG23PS20 Mutation-Positive (%)**			0.257
CLASS01=MUTATION	17 (8.0)	20 (12.0)	
ABSENT	195 (92.0)	147 (88.0)	

Supplementary Table S3. Top Genomic Signatures collected from CRC Population Subgroups.

Subgroup	*ID*	*Class*	*Number of Genes*	*Clinical Associations*	*J-value Score*
**CRC Stage02**	MG02PS04	*Mutation-Positive*	56	CMS1 Subtype<br>Disease-Free	140.36
		Mutation-Negative	30	Any Except CMS1<br>Recurrence Event	
**CRC Stage03**	MG03PS19	*Mutation-Positive*	11	CMS1 Subtype<br>Disease-Free	113.65
		Mutation-Negative	5	Any Except CMS1<br>Recurrence Event	
**CRC Stages 02 & 03**	MG23PS19	*Mutation-Positive*	61	Any Except CMS1<br>Disease-Free	127.51
		Mutation-Negative	33	CMS1 Subtype<br>Recurrence Event	

Supplementary Table S4. Marker lists for each genomic signature.

Provided as separated Excel file.

Supplementary Table S5. Genomic Signatures from CRC Subgroups for Survival Analysis.

	ID	Class	Number of Genes	Clinical Indications
CRC STAGE 02	MG02PS03	*Mutation-Negative*	40	Any Except CMS1/Primary Tumor *(Colon)*
	MG02PS04	*Mutation-Negative*	30	Any Except CMS1/Recurrence Event
	MG02PS05	*Mutation-Positive*	6	Disease-Free
CRC STAGE 03	MG03PS18	*Mutation-Negative*	4	30-60 months – Recurrence Event
	MG03PS19	*Mutation-Positive*	11	CMS1 Subtype/Recurrence Event
	MG03PS19	*Mutation-Negative*	5	Any Except CMS1/Recurrence Event
CRC STAGES 02 & 03	MG23PS19	*Mutation-Positive*	61	CMS1 Subtype/Recurrence Event
	MG23PS19	*Mutation-Negative*	33	Any Except CMS1/Recurrence Event
	MG23PS20	*Mutation-Positive*	7	Disease-Free

Supplementary Table S6. Hazard Ratios from Survival Analysis.

Variable	*Coefficient*	*SE*	*HR*	*95% CI*	*p-value*
CMS2	-1.2	0.325	0.3	0.16, 0.57	<0.001
CMS3	-1.03	0.422	0.36	0.16, 0.82	0.015
CMS4	-0.31	0.332	0.73	0.38, 1.41	0.35
MG02PS03.Class02	10.4	0.891	31,566	5,502, 181,100	<0.001
MG02PS04.Class02	-10.1	0.821	0	0.00, 0.00	<0.001
MG02PS05.Class01	0.218	0.461	1.24	0.50, 3.07	0.64
CMS2	-0.016	0.455	0.98	0.40, 2.40	0.97
CMS3	-0.126	0.647	0.88	0.25, 3.13	0.85
CMS4	-0.126	0.478	0.88	0.35, 2.25	0.79
MG03PS18.Class02	0.159	0.367	1.17	0.57, 2.41	0.66
MG03PS19.Class02	0.005	0.362	1	0.49, 2.04	0.99
MG03PS19.Class01	-9.99	0.697	0	0.00, 0.00	<0.001
CMS2	0.018	0.543	1.02	0.35, 2.95	0.97
CMS3	-0.262	0.663	0.77	0.21, 2.82	0.69
CMS4	-0.156	0.544	0.86	0.29, 2.48	0.77
MG23PS19.Class02	-0.113	0.595	0.89	0.28, 2.86	0.85
MG23PS19.Class01	-0.997	0.575	0.37	0.12, 1.14	0.083
MG23PS20.Class01	-0.492	0.43	0.61	0.26, 1.42	0.25

SE: Standard Error
HR: Hazard Ratio
95% CI: 95% Confidence Interval

Supplementary Table S7. Mutation frequency in studied samples.

Provided as a separated Excel file.

Supplementary Table S8. Feature Descriptions for variables used in exploratory analysis.

FEATURES	*DESCRIPTION*
F1CDx GENE PANEL	Foundation One FDA-approved genes
MICROSATELLITE	Microsatellite instability classification status
CMS CLASS	Consensus molecular subtype classification
DFS STATUS	Disease free status since initial treatment
DFS MONTHS	Disease free (months) since initial treatment
AJCC T STAGE	Describes the original primary tumor
AJCC M STAGE	Metastatic indications of primary tumor
AJCC N STAGE	Involvement of regional lymph nodes
LYMPHOVASCULAR INVASION	Invasion of the lymph-nodes by cancer
PERINEURAL INVASION	Invasion of cancer to perineural nerve space
VASCULAR INVASION	Vascular invasion of cancer indicating metastasis
KRAS MUTATION	*KRAS* gene mutation: Present or Absent
SITE OF TUMOR	Location of the Primary Tumor
ETHNICITY	Ethnic background of the patient
RACE	Demographic background of patient
AGE	Age cancer is first diagnosed [years]
SEX	Sex at birth of the patient
BMI	Body Mass Index [kg/m^2]

29. Cerami E, Gao J, Dogrusoz U, et al. The cBio cancer genomics portal: an open platform for exploring multidimensional cancer genomics data. Cancer Discov. May 2012;2(5):401-4. doi:10.1158/2159-8290.CD-12-0095

30. Gao J, Aksoy BA, Dogrusoz U, et al. Integrative analysis of complex cancer genomics and clinical profiles using the cBioPortal. Sci Signal. Apr 2 2013;6(269):pl1. doi:10.1126/scisignal.2004088

31. Cancer Genome Atlas N. Comprehensive molecular characterization of human colon and rectal cancer. Nature. Jul 18 2012;487(7407):330-7. doi:10.1038/nature11252

32. Wickham H, Averick M, Bryan J, et al. Welcome to the Tidyverse. Journal of Open Source Software. 2023;4(43):1686. doi:10.21105/joss.01686

33. Buuren Sv, Groothuis-Oudshoorn K. mice: Multivariate Imputation by Chained Equations in R. Articles. https://wwwjstatsoftorg/indexphp/jss. 2011-12-12

34. Borgan Ø. Modeling Survival Data: Extending the Cox Model. Terry M. Therneau and Patricia M. Grambsch, Springer-Verlag, New York, 2000. No. of pages: xiii + 350. Price: $69.95. ISBN 0-387-98784-3. Statistics in Medicine. 2001;20(13):2053-2054. doi:https://doi.org/10.1002/sim.956

35. Grothendieck G. sqldf: Manipulate R Data Frames Using SQL. Comprehensive R Archive Network (CRAN). https://cran.r-project.org/web/packages/sqldf/index.html

36. Therneau TM LT, Atkinson E, Crowson C. A Package for Survival Analysis in R. R package version 3.5-3. Comprehensive R Archive Network (CRAN). https://cran.r-project.org/web/packages/survival/index.html

37. Kassambara A KM, Biecek P, Fabian S. survminer: Drawing Survival Curves using 'ggplot2'. R package version 0.4.9. Comprehensive R Archive Network (CRAN). https://cran.r-project.org/web/packages/survminer/index.html
38. Sjoberg DD BM, Fruechtenicht C, Haesendonckx S, Treis T. ggsurvfit: Flexible Time-to-Event Figures. R package version 0.2.1. Comprehensive R Archive Network (CRAN). https://cran.r-project.org/web/packages/ggsurvfit/index.html
39. CRAN - Package ROCit. Comprehensive R Archive Network (CRAN); 2023. https://cran.r-project.org/web/packages/ROCit/index.html
40. Pardo MC, Franco-Pereira AM. Non Parametric ROC Summary Statistics. REVSTAT-Statistical Journal. 01/14 2022;15(4):583–600. doi:10.57805/revstat.v15i4.228
41. Guo K. VennDetail. http://bioconductor.org/packages/VennDetail/
42. Ishwaran H, Gerds TA, Kogalur UB, Moore RD, Gange SJ, Lau BM. Random survival forests for competing risks. Biostatistics. Oct 2014;15(4):757-73. doi:10.1093/biostatistics/kxu010
43. Ishwaran HUBK. Fast Unified Random Forests for Survival, Regression, and Classification (RF-SRC). 2023;doi:https://CRAN.R-project.org/package=randomForestSRC
44. Ishwaran H, Kogalur UB, Blackstone EH, Lauer MS. Random survival forests. Text. https://doiorg/101214/08-AOAS169. 2008-09-01 2008;doi:10.1214/08-AOAS169
